# Characterization of early markers of disease in the mouse model of mucopolysaccharidosis IIIB

**DOI:** 10.1186/s11689-024-09534-z

**Published:** 2024-04-17

**Authors:** Katherine B. McCullough, Amanda Titus, Kate Reardon, Sara Conyers, Joseph D. Dougherty, Xia Ge, Joel R. Garbow, Patricia Dickson, Carla M. Yuede, Susan E. Maloney

**Affiliations:** 1 Department of Psychiatry, Washington University School of Medicine, St. Louis, MO 63110 USA; 2 Department of Genetics, Washington University School of Medicine, St. Louis, MO 63110 USA; 3 Intellectual and Developmental Disabilities Research Center, Washington University School of Medicine, St. Louis, MO 63110 USA; 4 Department of Radiology, Washington University School of Medicine, St. Louis, MO 63110 USA; 5 Department of Pediatrics, Washington University School of Medicine, St. Louis, MO 63110 USA; 6 Department of Neurology, Washington University School of Medicine, St. Louis, MO 63110 USA; 7 Department of Neuroscience, Washington University School of Medicine, St. Louis, MO 63110 USA

**Keywords:** Sanfilippo B, Mucopolysaccharidosis IIIB, Lysosomal storage disorder, Ultrasonic vocalization, Gait, Fear conditioning, Startle response, MRI/DTI, Dominance, Aggression

## Abstract

**Background:**

Mucopolysaccharidosis (MPS) IIIB, also known as Sanfilippo Syndrome B, is a devastating childhood disease. Unfortunately, there are currently no available treatments for MPS IIIB patients. Yet, animal models of lysosomal storage diseases have been valuable tools in identifying promising avenues of treatment. Enzyme replacement therapy, gene therapy, and bone marrow transplant have all shown efficacy in the MPS IIIB model systems. A ubiquitous finding across rodent models of lysosomal storage diseases is that the best treatment outcomes resulted from intervention prior to symptom onset. Therefore, the aim of the current study was to identify early markers of disease in the MPS IIIB mouse model as well as examine clinically-relevant behavioral domains not yet explored in this model.

**Methods:**

Using the MPS IIIB mouse model, we explored early developmental trajectories of communication and gait, and later social behavior, fear-related startle and conditioning, and visual capabilities. In addition, we examined brain structure and function via magnetic resonance imaging and diffusion tensor imaging.

**Results:**

We observed reduced maternal isolation-induced ultrasonic vocalizations in MPS IIIB mice relative to controls, as well as disruption in a number of the spectrotemporal features. MPS IIIB also exhibited disrupted thermoregulation during the first two postnatal weeks without any differences in body weight. The developmental trajectories of gait were largely normal. In early adulthood, we observed intact visual acuity and sociability yet a more submissive phenotype, increased aggressive behavior, and decreased social sniffing relative to controls. MPS IIIB mice showed greater inhibition of startle in response to a pretone with a decrease in overall startle response and reduced cued fear memory. MPS IIIB also weighed significantly more than controls throughout adulthood and showed larger whole brain volumes and normalized regional volumes with intact tissue integrity as measured with magnetic resonance and diffusion tensor imaging, respectively.

**Conclusions:**

Together, these results indicate disease markers are present as early as the first two weeks postnatal in this model. Further, this model recapitulates social, sensory and fear-related clinical features. Our study using a mouse model of MPS IIIB provides essential baseline information that will be useful in future evaluations of potential treatments.

**Supplementary Information:**

The online version contains supplementary material available at 10.1186/s11689-024-09534-z.

## Background

Mucopolysaccharidosis (MPS) IIIB, also known as Sanfilippo Syndrome B, is a devastating childhood disease caused by a known lysosomal enzyme deficiency [1, 2]. Specifically, a complete loss of the lysosomal enzyme alpha-N-acetylglucosaminidase (NAGLU) results in the accumulation of heparin sulfate and serious neurological issues [3]. Disease onset occurs around age five with developmental and speech delays, and the rise of somatic signs like hearing loss [1–3]. Throughout the disease progression, patients experience cognitive deterioration, sleep disturbances, and behavioral difficulties including hyperactivity and aggression, which are among the most distressing symptoms, as reported by parents [4, 5]. In addition, these children show autistic-like social and emotional abnormalities. Imaging studies revealed cerebellar atrophy and white matter thinning accompany the behavioral symptomatology yet do not track with disease severity [6, 7]. As the disease progresses, these children develop a lack of fear, motor decline, and eventual onset of severe dementia and early death.

Regrettably, there are currently no available treatments for MPS IIIB patients. Yet, animal models of lysosomal storage diseases, including for MPS IIIB, have been valuable tools in identifying promising avenues of treatment, including gene therapy, bone marrow transplant, and enzyme replacement therapy [8–12]. While not FDA approved yet, both gene therapy and enzyme replacement therapy have been ongoing clinical trials [13, 14]. The MPS IIIB murine model recapitulates many aspects of the human disease, including altered circadian rhythms, juvenile-onset hearing loss, later-age onset of balance issues and phenotypes suggestive of reduced fear [15–19]. However, many important markers of MPS IIIB disease remain unexplored in the mouse model, such as aggression, visual decline, sensorimotor gating, and fear conditioning. Thus, the emerging therapeutics have not yet been evaluated against these phenotypes.

In addition, a ubiquitous finding across rodent models of lysosomal storage diseases is that the best treatment outcomes resulted from intervention prior to symptom onset [20–24]. Therefore, identifying the earliest age at onset of behavioral abnormalities is necessary to determine the ideal age for treatment. Specifically, the MPS IIIB murine model has not been evaluated for neurobehavioral phenotypes prior to four weeks of age, missing possible early markers of developmental delay [15–19]. In addition, this model has not been well-characterized for behaviors related to motor function such as gait, and social and emotional abnormalities, which would be key markers of disease for evaluation of therapy effectiveness.

In the current study we assessed phenotypes in early postnatal development and expanded our assays to include clinically-relevant behavioral domains not yet explored in this model. Histopathological and biochemical analyses of disease require tissue collection and euthanasia of the animal, and therefore only a single time point can be examined. Neurobehavioral analyses are non-invasive and allow for the coupling of multiple areas of assessment in a single animal. Thus, in addition to early communicative behaviors and developmental motor trajectories, we assessed social and agonistic behaviors, visual acuity, sensorimotor gating, and fear conditioning in MPS IIIB mice. We coupled our behavioral assessment with neuroimaging, including magnetic resonance imaging to examine whole brain volumes, cerebellar structure and diffusion tensor imaging to interrogate structural and white matter integrity, and correlated these data with behavioral phenotypes. The findings from our study provide baseline phenotypic information for the MPS IIIB model that will be useful in future evaluations of potential treatments.

## Methods

### Animals

All experimental protocols were approved by and performed in accordance with the relevant guidelines and regulations of the Institutional Animal Care and Use Committee of Washington University in St. Louis and were in compliance with US National Research Council's Guide for the Care and Use of Laboratory Animals, the US Public Health Service's Policy on Humane Care and Use of Laboratory Animals, and Guide for the Care and Use of Laboratory Animals.

All mice used in this study were maintained and bred in the vivarium at Washington University in St. Louis from an existing colony at Washington University. For all experiments, adequate measures were taken to minimize any pain or discomfort. The colony room lighting was on a 12:12 h light/dark cycle (lights on 6am to 6 pm); room temperature (~ 20–22 °C) and relative humidity (50%) controlled automatically. Standard lab diet and water were freely available. Upon weaning at postnatal day (P)21, mice for behavioral testing were group-housed according to sex and genotype.

The MPS IIIB mouse model, maintained on a C57BL/6J background, containing a neomycin cassette disruption of exon 6 in the *Naglu* gene was used for this study. Generation of this model was described previously [19]. *Naglu*^+/-^ (Control) mice were crossed with *Naglu*^−/−^ (MPS IIIB) mice to generate male and female Control and MPS IIIB littermates for the study. MPS IIIB is an autosomal recessive disorder, therefore heterozygous mutants are reportedly normal [17] and serve as the appropriate littermate control. We examined the phenotypes of three independent cohorts. The first cohort included 10 litters (4 from heterozygous control sire x MPS IIIB dam pairings and 6 from heterozygous control sire x dam pairs) comprising 27 Control (13 female and 14 male) and 17 MPS IIIB (4 female and 13 male) mice. The second cohort included 8 litters (3 from MPS IIIB sire x heterozygous control dam pairings and 5 from heterozygous control sire x dam pairs) comprising 30 Control (10 female and 20 male) and 16 MPS IIIB (8 female and 8 male) mice. The third cohort included 7 litters (3 from MPS IIIB sire x heterozygous control dam pairings, 2 from heterozygous control sire x MPS IIIB dam pairings, and 2 from heterozygous control sire x dam pairs) comprising 20 Control (10 female and 10 male) and 19 MPS IIIB (9 female and 10 male) mice.

### Behavioral tasks

**Fig. 1:**
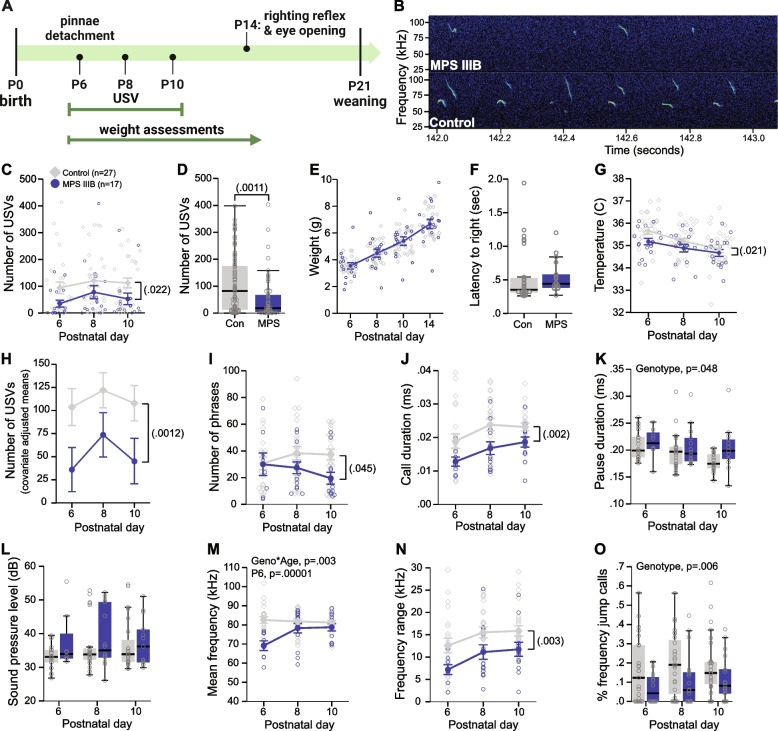
MPS IIIB postnatal pups exhibit reduced body temperature and USVs with altered spectrotemporal features. **A** Timeline of developmental assessments. **B** Representative spectrogram segment from a Control mouse and a MPS IIIB mouse. **C-D** Number of ultrasonic vocalizations (USVs) during maternal isolation-induced USV assay across ages (**C**) and collapsed for age (**D**). **E** Body weight across postnatal assessment ages.** F** Latency to exhibit righting reflex at P14. **G** Body temperatures recorded immediately prior to USV recordings at P6-10. **H** Covariate-adjusted means of number of USVs accounting for differences in body temperature. **I** Number of phrases of USV calls. **J** Average USV duration. **K** Average pause duration between USVs within a phrase. **L** Sound pressure level or volume of USV calls. **M** Mean USV frequency. **N** Frequency range of USV calls.** O** Percent of calls that included a frequency jump greater than 10 kHz. **C**,** E-J**,** M**, and** N** data are presented as means ± SEM. **D**,** K, L**, and** O** data are presented as boxplots with thick horizontal lines respective group medians, boxes 25th – 75th percentiles, and whiskers 1.5 × IQR. Individual data points are presented as open circles

**Fig. 2:**
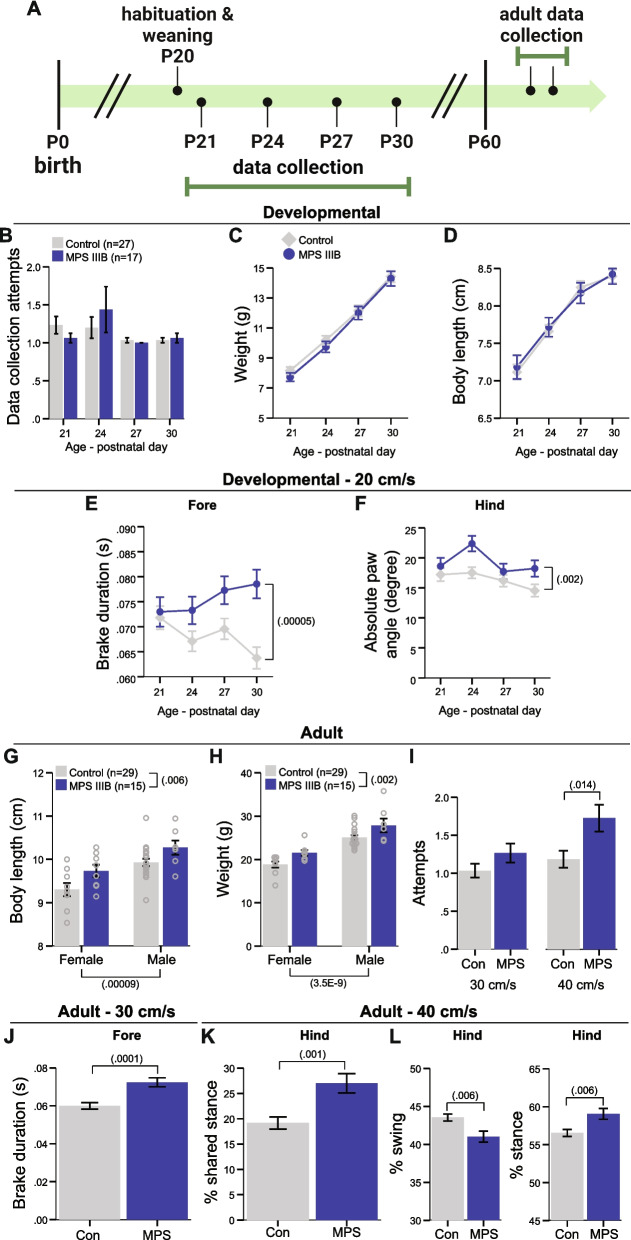
MPS IIIB mice exhibit subtle yet persistent alterations in gait. **A** Schematic of gait data collection timeline. **B** Number of data collection attempts needed across developmental timepoints. **C-D** Weight (**C**) and body length (**D**) across developmental gait collection ages. **E** Duration of braking for forelimbs during developmental gait assessment. **F** Hindpaw angle across developmental gait assessment timepoints. **G-H** Adult body length and weight for both females and males. **I** Number of data collection attempts needed for adult mice at 30 cm/s and 40 cm/s belt speeds. **J** Duration of braking for forelimbs at 30 cm/s belt speed for adult mice. **K** Percent stride in hindlimb shared stance at 40 cm/s belt speed for adult mice. **L** Percent of stride in swing (left panel) and stance (right panel) for hindlimbs at 40 cm/s belt speed for adult mice. Observed *p* values reported in figures. **B-D**,** G**, and** H** Grouped data presented as means ± SEM with individual data points presented as open circles. **E**,** F**, and **I-L** Covariate-adjusted means ± SEM accounting for changes in body length presented

**Fig. 3:**
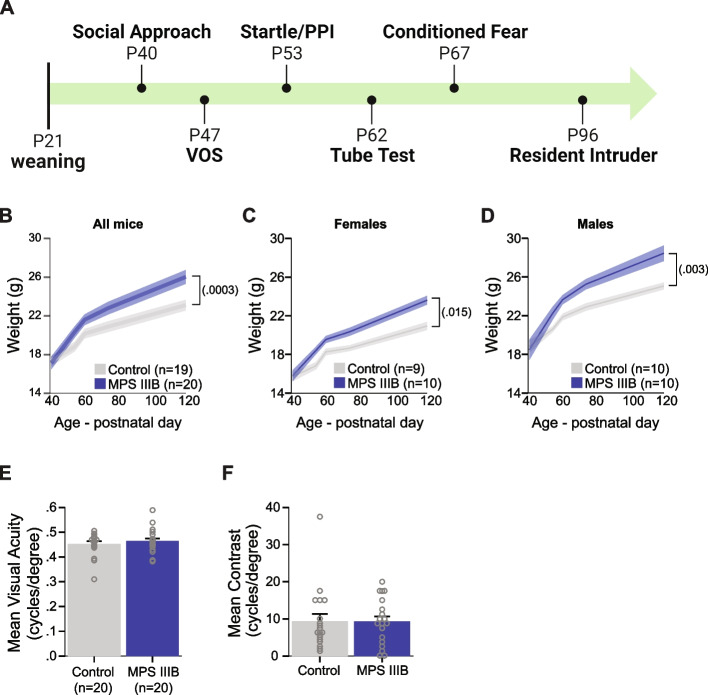
MPS IIIB mice weigh significantly more than controls with unaltered vision in adolescence. **A** Schematic of behavioral testing timeline for cohort 3. **B-D** Body weights as measured from adolescence through four months of age for all mice (left panel), females only (middle panel), and male mice only (right panel). **E–F** VOS measurements of mean visual acuity levels (**E**) and mean contrast levels (**F**). **B-F** Grouped data presented as means ± SEM with individual data points presented as open circles

All behavioral data were collected in the Animal Behavior Subunit of the Intellectual and Developmental Disabilities Research Center at Washington University in St. Louis. Cohorts 1 and 2 were assessed for developmental trajectories from early postnatal ages through young adulthood (Table 1; Figs. 1A, 2A). Cohort 3 was assessed for additional, clinically-relevant behavioral phenotypes beginning in adolescence through early adulthood (Table 1; Fig. 3A). For all tasks, the mice were acclimated to the testing room at least 30 min prior to the start of testing. All assays were conducted by female experimenters blinded to experimental group designations during testing, and all testing occurred during the light phase. Order of tests (Table 1) was chosen to minimize effects of stress.

**Table 1:** Order of and age at behavioral testing per cohort with related patient phenotype and specific assessment in mice

Cohort	Related Patient Phenotype	Specific Assessment	Behavioral Test	Age
1	Developmental delay	Developmental growth assessment	Physical milestone inspection, reflex achievement, weight collection	P6-14
1	Speech delay	Developmental vocalization levels	Maternal isolation-induced USV recordings	P6-10
2	Motor impairment	Developmental gait trajectories	DigiGait assay	P21-30, P60
3	Altered body size trajectory	Bi-weekly weight measurements	–	–
	Health decline	Physical features and posture	Physical exam	P35
	Autistic-like social abnormalities	Sociability and social novelty	Social approach assay	P40
	Vision decline	Visual acuity and contrast	Virtual optomotor system assessment	P47
	Sensory deficits	Sensorimotor gating	Acoustic startle response/PPI task	P53
	Autistic-like social abnormalities	Social hierarchy behaviors	Social dominance tube test	P62
	Reduction in fear	Associative anxiety and conditioning	Fear conditioning test	P67
	Aggression	Aggressive and agonistic behaviors	Resident intruder assay	P96

### Maternal isolation-induced ultrasonic vocalizations

During the first two weeks postnatal, we assessed the control and MPS IIIB littermates in cohort 1 for signs of gross developmental delay, communicative delay or motor delay (Fig. 1A; Table 1), following our previously published methods [25]. To evaluate gross development, the mice were weighed daily from P6—P14, and evaluated for physical milestones of development including pinna detachment by P6 and eye opening by P14. While human language cannot be explored in mice, vocal communication behavior is conserved across taxa [26]. Ultrasonic vocalizations (USVs) produced by the mouse pup is one of the earliest forms of social communication we can examine in the mouse, and these isolation calls elicit maternal care from the dam [27]. This behavior also has a developmental trajectory, beginning just after birth, peaking during the first week postnatal and disappearing around P14, making it useful for examining delay in early social circuits. Tissue was collected from all pups on P5 for genotyping. USVs were recorded on P6, P8, and P10 following our previously published methods [25, 28]. Briefly, the dam was removed from the nest and the litter placed in a warming cabinet to maintain body temperature while away from the dam. The surface temperature of each pup was recorded (HDE Infrared Thermometer) prior to placement in an empty polycarbonate cage (28.5 cm × 17.5 cm x 12 cm) in a sound-attenuating chamber. USVs were recorded for three minutes using an Avisoft UltraSoundGate CM16 microphone, Avisoft UltraSoundGate 116H amplifier, and Avisoft Recorder software (gain = 8 dB, 16 bits, sampling rate = 250 kHz). The pup was then weighed and returned to the nest. The dam was returned to the nest following recording of the last pup in the litter. Frequency sonograms were prepared from recordings using MATLAB (frequency range = 25 kHz to 120 kHz, FFT size = 512, overlap = 50%, time resolution = 1.024 ms, frequency resolution = 488.2 Hz). Individual syllables and other spectral features were identified and counted from the sonograms as previously described [29, 30]. To assess achievement of motor milestones, the surface righting reflex was assessed for each pup on P14 [25]. Each pup was placed on its back in an empty cage lined with a plastic bench pad and the time to return to a prone position was recorded up to 60 s. Three trials were averaged for analysis.

### DigiGait

Developmental gait trajectories were assessed using the DigiGait Gait Analysis System (Mouse Specifics, Inc) following our previously published methods [31, 32]. Briefly, for all mice in cohort 2, gait metrics were assessed on P21, 24, 27, 30 at 20 cm/s and again at one time point after P60 (adulthood) at both 30 cm/s and 40 cm/s (Fig. 2A; Table 1). Prior to P21 testing, mice were habituated to the DigiGait on P20 by placing the mouse on the dark, motionless apparatus belt for two minutes, followed by two minutes in the light. Then, the belt was turned on starting at 5 cm/s and increased slowly to 20 cm/s until the mouse had about 30 s of uninterrupted running. The litters were weaned from the dam and sire prior to testing on P21. On test days, a ‘failure to run’ criterion of five attempts was implemented. If the animal failed to run on the belt after five attempts, it was returned to its homecage. Video processing and body length assessments were conducted as previously described [31, 32].

### Social approach

Three chamber social approach task was used to assess sociability and preference for social novelty at P40 (Fig. 5A) using procedures adapted from our previous work [25]. Briefly, stimulus partner mice were sex-, age- and strain-matched. The full apparatus measured 60 cm x 39 cm x 22 cm with the center chamber (9.75 cm x 39 cm x 22 cm) and outer chambers (24.2 cm x 39 cm x 22 cm) separated by opaque walls. Stimulus cups were placed 2 cm from the external wall. Testing was completed in white lighting at 115 lx. Behavior of the animals was tracked using Any-maze tracking software (Stoelting, Co). Between animals, the stimulus cups were cleaned with 70% ethanol and the acrylic apparatus was cleaned with 0.02% unscented Chlorhexidine.

### Virtual Optomotor System

Visual acuity and contrast testing was completed using the Virtual Optomotor System (VOS; Cerebral Mechanics Inc.) adapted from previous methods [33, 34]. Testing assessed the sharpness of vision and the ability to distinguish between the foreground and background at P47. The VOS system apparatus consisted of a chamber (52.5 cm × 52.2 cm x 95 cm) with a centered platform (5.5 cm diameter) raised 19 cm surrounded by 4 monitors. To measure acuity, vertical gratings rotated across the screens and varied sizes across testing until a threshold was met at which mice could no longer distinguish the moving lines. A response was measured by the reflexive head movement (optomotor reflex) in concert with the rotation, scored visually by experimenter. Visual contrast is the ability to distinguish the foreground from the background. Contrast assessment used a constant grating size (0.128 cycles/degree), with gratings varying in shade until the mouse could no longer distinguish movement and elicit the optomotor reflex. The direction of movement in the VOS system allowed for each eye to be tested independently. Threshold values of clockwise (CW) and counterclockwise (CCW) were analyzed and averaged to create variables for mean acuity and mean contrast. Testing was completed between 6am and 12 pm when mice are less active and thus the response more readily distinguished. Mice were habituated to the apparatus 1 day prior to testing for 10 min, with the screens on but no stimulus present. Acuity testing occurred on day 2 and contrast testing on day 3. Females and males were tested on alternating days. Fifty trials were completed per eye using a staircase method to determine threshold. Animals with extreme outlier values were retested at the end of the day for accurate assessment.

### Acoustic startle response/prepulse inhibition

Sensorimotor gating and startle reflex was evaluated at P53 using the Acoustic Startle/Prepulse Inhibition (PPI) assay following our previously published methods [25]. Startle response to a 120 dB auditory stimulus pulse (40 ms broadband burst) and PPI (response to a prepulse plus the startle pulse) were measured concurrently in the mice. Beginning at stimulus onset, 1 ms force readings were averaged to obtain an animal's startle amplitude. A total of 20 startle trials were presented over a 20 min test period during which the first 5 min served as an acclimation period when no stimuli above the 65 dB white noise background were presented. The session began and ended by presenting 5 consecutive startle (120 db pulse alone) trials unaccompanied by other trial types. The middle 10 startle trials were interspersed with PPI trials (consisting of an additional 30 presentations of 120 dB startle stimuli preceded by pre-pulse stimuli of either 4, 8, or 16 dB above background (10 trials for each PPI trial type). A percent PPI score for each trial was calculated using the following equation: %PPI = 100*(ASRstartle pulse alone—ASRprepulse + startle pulse)/ASRstartle pulse alone. Startle responses to increasing decibel levels was used as an auditory function screen at the end of the PPI trial session, and a paired t-test for response to 10 120 dB startle trials vs non-startle trials was used to determine deafness.

### Social dominance tube test

Laboratory mice acquire dominance behaviors to create social hierarchy ranks within their cage environments, or social groups, between six to eight weeks of age, which can be leveraged to examine normal social dominance behavior. Thus, we used the Tube Test to assess social dominance behavior at P62 between sex-matched control and MPS IIIB animal pairs following our previously published methods (Fig. 5J) [25].

### Conditioned fear

**Fig. 4:**
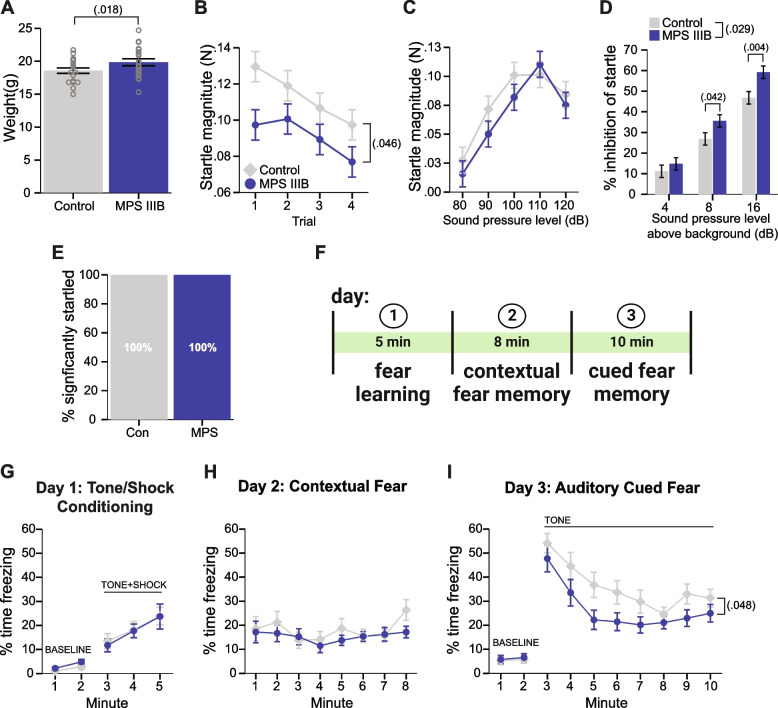
MPS IIIB mice exhibit altered responses to negative stimuli suggestive of reduced fear by early adulthood. **A** Weight measurements recorded during Acoustic Startle/PPI task. **B** Startle response to 120 dB acoustic stimulus across 4 trials. **C** Startle response to consecutive trials of acoustic stimuli presentations of increasing dB level. **D** Inhibition of startle response to 120 dB stimuli following prepulse tone 4, 8, or 16 dB above background. **E** Percent of mice per group that exhibited a significant difference in response between startle and non-startle trials. **F** Schematic of fear conditioning paradigm. **G** Percent time freezing during baseline and tone/shock conditioning on day one. **H** Percent time freezing in response to fear context on day two. **I** Percent time freezing on day three during baseline in a new context and during tone presentation. **A**, and **G-I** Grouped data are presented as means ± SEM with individual data points presented as open circles. **B-D** Grouped data are presented as covariate adjusted means ± SEM accounting for differences in body weight. **E** Data are presented as counts

Associative anxiety-related memory was assessed at P67 in the conditioned fear task (Fig. 4F). The procedure details were as we previously published [25]. Briefly, the animals were exposed to a 1.0 mA current paired with a 80 dB white noise tone three times on day 1. Contextual fear conditioning was assessed on day 2 by placing the animal back into the chambers from day 1 without shock or tone presentation. On day 3, the animals were placed into a contextually altered chamber and assessed for cued fear conditioning in the presence of the 80 dB tone from day 1. On all three days, freezing was quantified by FreezeFrame software (Actimetrics). Sensitivity to foot shocks was evaluated one week following the start of conditioned fear testing to confirm differences in freezing behavior were not confounded by differences in reactivity to the shock current, based on our previously published methods [35]. Mice were placed in the fear conditioning chambers and exposed to 3 s footshock every 20–30 s starting at 0.05 mA and increasing in intensity by 0.05 mA until a flinch, vocalization and escape behavior were elicited. The current at which each behavior occurred was recorded.

### Resident intruder assay

To evaluate aggressive and agonistic behaviors, we tested P96 male mice in the resident intruder paradigm based on our previous study [36]. Male mice were single housed and cages remained unchanged for ten days prior to testing to establish a territory. On day 6, a sexually naive strain- and age-matched female was added to each male cage and removed 24 h prior to testing to potentiate territorial behaviors. Intruders used were strain- and age-matched novel male mice bred in the same colony space. On the test day, under white light, the intruder mouse was added directly into the resident home cage in a sound-attenuating box, and allowed to freely interact for 10 min. Interactions were filmed with a digital camera (Sony HandyCam). Upon completion, the intruder was removed and placed in a clean holding cage until all cage-mates complete testing. This was repeated over two additional days with a novel intruder mouse. We monitored interactions for any attacks drawing blood, which would necessitate prematurely ending the assay. However, we did not observe any attacks at this level.

To process the videos, we used pose estimation software (DeepLabCut, version 2.2.1) to track body parts in space and time [37, 38], building upon a previously built model [39]. Specifically, we labeled 50 frames taken from 60 videos plus an additional 1700 frames of closely interacting animals, and 95% were used for training. We used a dlcrnet_ms5-based neural network with default parameters for 200,000 training iterations. We validated with two shuffles, and found the test error was 7.95 pixels, with the train error at 5.58 pixels (image size was 1920 by 1080). We then used a p-cutoff of 0.6 to condition the X,Y coordinates for future analysis. This network was then used to analyze all videos in the experiment.

The pose estimation was then fed through the Simple Behavior Analysis (SimBA) random forest classifier [40] to extract attack behavior. Training videos were annotated for attack behavior using the SimBA event-logger using 180 annotated behavior files, downloaded from https://osf.io/tmu6y/ in addition to four in-house annotated videos. All training files were annotated according to definitions found in the simBA preprint [39, 40]. Random forest classifiers were trained using default hyperparameters, and classifier performances were evaluated. We set the discrimination threshold for scramble attacks to 0.65. The minimum bout length was 200 ms. Once classifiers were validated, predictive classifiers were applied to all videos in the dataset. Statistics regarding outcome variables of total time, the number of frames, total number of instances, mean instance interval, and mean interval between behavior instances were generated.

### Magnetic resonance imaging

All in vivo magnetic resonance imaging (MRI) experiments were performed on a 9.4 T Bruker BioSpec 94/20 MR system (Bruker, Ettlingen, Germany) equipped with an 11.4-cm inner-diameter gradient insert. Data were collected with a 4-channel-array receive-only mouse-brain CryoProbe in combination with a quadrature transmit-only coil. Mice were anesthetized using 1.0–1.5% isoflurane/O_2_ and were secured in the prone position. Respiratory rate and rectal temperature were monitored using a small-animal physiological monitoring system (SA Instruments; Stony Brook, NY) and mouse body temperature was maintained at 37 °C using a circulating warm water pad.

### Diffusion tensor imaging

Diffusion tensor imaging (DTI) data were collected using a spin-echo diffusion-weighted sequence with 31 b-values, max b-value = 1000 s/mm^2^. Other MR acquisition parameters include repetition time (TR) = 1.5 s; echo time (TE) = 30 ms; diffusion-encoding times, Δ = 18 ms, δ = 6 ms; field of view = 16 mm × 16 mm; matrix size = 128 × 128; 31 axial slices of thickness 0.5 mm. The total acquisition time was approximately one hour.

### DTI data processing

DTI datasets were analyzed via non-linear least squares modeling of the diffusion-weighted signal using MATLAB (The MathWorks, Natick MA). Firstly, the data were zero-padded to matrix size 256 × 256, resulting in an in-plane resolution of 62.5 mm × 62.5 mm. The Gibbs-ringing artifact was reduced using a previously published method [41] followed by Gaussian filtering (Sigma 0.75) to denoise and smooth images. Tensor eigenvalues and parametric maps of apparent diffusion coefficient (ADC) and fractional anisotropy (FA) were calculated according to standard equations [42, 43].

All parametric maps were converted to NIfTI format for subsequent analysis. Whole brain (15.5 mm in anterior–posterior direction) and brain-substructure regions of interest (ROI) were segmented manually using ITK-SNAP [44]. Total brain volumes were calculated by segmenting T2-weighted (b = 0) images. The cerebellum and corpus callosum (CC) were segmented based on T2-weighted images and maps of ADC and FA. Volumes and DTI parameters were extracted for subsequent statistical analysis. Cortical thickness measurements were conducted using ITK-SNAP based on previously published methods [45]. A ventral to dorsal measurement was taken from the peak of the cingulum bundle to the dorsal surface of the cortex. This was repeated in both hemispheres across two sections for each brain, which were averaged for analysis.

### Statistical analyses

Statistical analyses and data visualization were conducted using IBM SPSS Statistics (v.28). Prior to analyses, data were screened for missing values and fit of distributions with assumptions underlying univariate analysis. This included the Shapiro–Wilk test on z-score-transformed data and qqplot investigations for normality, Levene’s test for homogeneity of variance, and boxplot and z-score (± 3.29) investigation for identification of influential outliers. Means and standard errors were computed for each measure. Linear modeling including mixed designs and analysis of variance (ANOVA), including repeated measures and covariate designs, were used to analyze data where appropriate, and simple main effects were used to dissect significant interactions. Sex was included as a biological variable in all analyses across all experiments. Where appropriate, the Greenhouse–Geisser or Huynh–Feldt adjustment was used to protect against violations of sphericity for repeated measures designs. Multiple pairwise comparisons were subjected to Bonferroni correction or the Benjamini–Hochberg method for false discovery rate, where appropriate. For data that did not fit univariate assumptions, non-parametric tests were used or data transformations were applied. Sex*genotype effects are reported where significant, otherwise data are discussed and visualized collapsed for sex, with details of all statistical tests and results reported in Table 2. Non-significant predictors were removed from final models to achieve the most parsimonious models. The critical alpha value for all analyses was *p* < 0.05 unless otherwise stated. Pearson’s correlation was used to assess relationships between continuous outcomes and one-sample t-tests were used to assess differences from chance levels. Figure illustrations were generated using BioRender. The datasets generated and analyzed during the current study are available from the corresponding author upon reasonable request.

**Table 2:** Statistical analysis results

Figure	Outcome	Model	Predictor	Output	*p* value
1	Number of USVs	rmANOVA	Genotype	*F*(1,42) = 5.669	.022
			Sex	*F*(1,40) = 3.352	.075
			Age	*F*(2,84) = 1.894	.157
			Genotype* Age	*F*(2,84) = 0.863	.863
		Mann–Whitney	Genotype	*U*(132) = 1373	.0011
	Weight	rmANOVA	Genotype	*F*(1,42) = .016	.900
			Sex	*F*(1,40) = .001	.977
			Age	*F*(1.5,61) = 871.279	3.0E-44
			Genotype* Age	*F*(1.5,61) = .347	.640
	Latency to right	Mann–Whitney	Genotype	*U*(44) = 289.5	.148
			Sex	*U*(44) = 249.5	.629
	Temperature	rmANOVA	Genotype	*F*(1,42) = 5.706	.021
			Sex	*F*(1,40) = .386	.538
			Age	*F*(2,84) = 7.969	.001
			Genotype* Age	*F*(2,84) = .411	.664
	Number of USVs with temperature covariate	ANCOVA	Genotype	*F*(1,125) = 11.021	.001
			Age	*F*(2,125) = .911	.405
			Genotype* Age	*F*(2,125) = .111	.895
	Number of phrases	Linear mixed model	Genotype	*F*(1,42.3) = 4.264	.045
			Sex	*F*(1,39.9) = 2.935	.094
			Age	*F*(2,52.6) = .686	.508
			Genotype*Age	*F*(2,52.6) = 1.265	.291
	Call duration	Linear mixed model	Genotype	*F*(1,28) = 11.198	.002
			Sex	*F*(1,24.6) = 2.055	.164
			Age	*F*(2,41.3) = 3.679	.034
			Genotype*Age	*F*(2,41.3) = .282	.756
	Pause duration	Mann–Whitney	Genotype	*U*(102) = 892	.048
	Sound pressure level	Mann–Whitney	Genotype	*U*(102) = 972	.159
	Mean frequency	Linear mixed model w/* simple main effects*	Genotype	*F*(1,77.1) = 8.973	.004
			Sex	*F*(1,68.7) = .156	.694
			Age	*F*(2,96) = 4.364	.015
			Genotype*Age	*F*(2,96) = 6.274	.003
			*P6, Genotype*	*F*(1,96) = 20.942	.00001
	Frequency range	Linear mixed model	Genotype	*F*(1,66.7) = 9.557	.003
			Sex	*F*(1,76) = 3.623	.061
			Age	*F*(1,96) = 5.084	.008
			Genotype*Age	*F*(1,96) = .280	.757
	% calls with frequency jump	Mann–Whitney	Genotype	*U*(127) = 1348.5	.006
2	Developmental data collection attempts	rmANOVA	Genotype	*F*(1,42) = .144	.706
			Sex	*F*(1,42) = .144	.706
			Age	*F*(1.5,68) = 2.545	.096
			Genotype*Age	*F*(1.5,68) = .992	.361
	Developmental body weight	rmANOVA	Genotype	*F*(1,42) = .007	.932
			Sex	*F*(1,42) = 12.837	.0009
			Age	*F*(1.9,80.3) = 1294.4	1.9E-94
			Genotype*Age	*F*(1.9,80.3) = 3.599	.034^
	Developmental body length	rmANOVA	Genotype	*F*(1,42) = .504	.481
			Sex	*F*(1,42) = 16.008	.0003
			Age	*F*(3,126) = 89.130	5.3E-31
			Genotype*Age	*F*(3,126) = .214	.887
	Developmental forelimb brake duration	Hierarchical mixed covariate model	Genotype	*F*(1,71.4) = 18.499	.00005 (.002*)
			Sex	*F*(1,82.9) = .785	.378
			Age	*F*(3,127.9) = .731	.536
			Genotype*Age	*F*(3,130.6) = 2.734	.046 (.510*)
	Developmental hindlimb absolute paw angle	Hierarchical mixed covariate model	Genotype	*F*(1,65.1) = 10.979	.002 (.033*)
			Sex	*F*(1,79.5) = 2.581	.112
			Age	*F*(3,124.8) = 3.604	.015 (.045*)
			Genotype*Age	*F*(3,128.6) = 1.384	.251
	Adult body length	ANOVA	Genotype	*F*(1,40) = 8.278	.006
			Sex	*F*(1,40) = 18.846	.00009
			Genotype*Sex	*F*(1,40) = .107	.745
	Adult body weight	ANOVA	Genotype	*F*(1,40) = 10.809	.002
			Sex	*F*(1,40) = 59.719	3.5E-9
			Genotype*Sex	*F*(1,40) = .001	.972
	30 cm/s data collection attempts	ANOVA	Genotype	*F*(1,42) = 2.261	.140
			Sex	*F*(1,40) = 1.222	.276
			Genotype*Sex	*F*(1,40) = .629	.433
	40 cm/s data collection attempts	ANOVA	Genotype	*F*(1,36) = 6.746	.014
			Sex	*F*(1,34) = .089	.767
			Genotype*Sex	*F*(1,34) = .831	.368
	Adult 30 cm/s forelimb brake duration	ANCOVA	Genotype	*F*(1,41) = 17.812	.0001 (.0006*)
			Sex	*F*(1,39) = 2.067	.308
			Genotype*Sex	*F*(1,39) = 1.473	.232
	Adult 40 cm/s hindlimb % shared stance	ANCOVA	Genotype	*F*(1,35) = 12.092	.001 (.06*)
			Sex	*F*(1,33) = .114	.738
			Genotype*Sex	*F*(1,33) = .042	.838
	Adult 40 cm/s hindlimb % swing	ANCOVA	Genotype	*F*(1,35) = 8.555	.006 (.088*)
			Sex	*F*(1,33) = .814	.374
			Genotype*Sex	*F*(1,33) = .030	.863
	Adult 40 cm/s hindlimb % stance	ANCOVA	Genotype	*F*(1,35) = 8.555	.006 (.088*)
			Sex	*F*(1,33) = .814	.374
			Genotype*Sex	*F*(1,33) = .030	.863
3	Body weight	rmANOVA w/ *simple main effects*	Genotype	*F*(1,35) = 16.179	.0003
			Sex	*F*(1,35) = 93.839	1.9E-11
			Genotype*Sex	*F*(1,35) = .139	.712
			*Females, Genotype*	*F*(1,35) = 6.484	.015
			*Males, Genotype*	*F*(1,35) = 9.927	.003
			Age	*F*(2,72.9) = 221.81	3.7E-32
			Genotype*Age	*F*(2,72.9) = 9.667	.0002
	Mean visual acuity	ANOVA	Genotype	*F*(1,37) = .573	.454
			Sex	*F*(1,35) = 2.732	.107
			Genotype*Sex	*F*(1,35) = .436	.513
	Mean contrast	ANOVA	Genotype	*F*(1,37) = .003	.960
			Sex	*F*(1,35) = 2.409	.130
			Genotype*Sex	*F*(1,35) = 1.034	.316
4	Body weight during Acoustic Startle/PPI testing	ANOVA	Genotype	*F*(1,36) = 6.187	.018
			Sex	*F*(1,36) = 27.528	7.1E-6
			Genotype*Sex	*F*(1,36) = .136	.714
	Startle magnitude – 120 dB	rmANCOVA	Genotype	*F*(1,37) = 4.282	.046
			Sex	*F*(1,35) = .823	.371
			Trial	*F*(3,111) = .338	.798
			Genotype*Trial	*F*(3,111) = 1.216	.307
	Startle magnitude – 80-120 dB	rmANCOVA	Genotype	*F*(1,37) = .921	.343
			Sex	*F*(1,35) = .042	.839
			dB	*F*(3.8,143.8) = 3.208	.016
			Genotype*dB	*F*(3.8,143.8) = .192	.939
	% inhibition of startle	rmANCOVA w/ *simple main effects*	Genotype	*F*(1,37) = 5.151	.029
			Sex	*F*(1,35) = 2.035	.163
			dB	*F*(2,74) = .046	.955
			Genotype*dB	*F*(2,74) = 1.033	.361
			*4 dB, Genotype*	*F*(1,113) = .712	.401
			*8 dB, Genotype*	*F*(1,113) = 4.221	.042
			*16 dB, Genotype*	*F*(1,113) = 8.444	.004
	% freezing: tone + shock paring	rmANOVA	Genotype	*F*(1,35) = .027	.872
			Sex	*F*(1,35) = 9.960	.003
			Minute	*F*(2,70) = 10.981	.00007
			Genotype*Minute	*F*(2,70) = .053	.949
	% freezing: contextual fear	rmANOVA	Genotype	*F*(1,35) = .592	.447
			Sex	*F*(1,35) = 5.635	.023
			Minute	*F*(7,245) = 2.506	.017
			Genotype*Minute	*F*(7,245) = 1.049	.397
	% freezing: cued fear	rmANOVA	Genotype	*F*(1,35) = 4.210	.048
			Sex	*F*(1,35) = 2.129	.153
			Minute	*F*(7,245) = 18.164	2.1E-19
			Genotype*Minute	*F*(7,245) = .670	.697
5	SA distance traveled	rmANOVA	Genotype	*F*(1,36) = .943	.338
			Sex	*F*(1,36) = 1.674	.204
			Genotype*Sex	*F*(1,36) = .051	.823
	SA habituation investigation time	rmANOVA	Genotype	*F*(1,36) = .538	.468
			Stimulus	*F*(1,36) = .645	.427
			Sex	*F*(1,36) = 6.231	.017
			Genotype*Stimulus	*F*(1,36) = .004	.950
			Genotype*Sex	*F*(1,36) = .018	.893
	SA sociability preference index	ANOVA	Genotype	*F*(1,36) = .886	.353
			Sex	*F*(1,36) = 5.800	.021
			Genotype*Sex	*F*(1,36) = 2.992	.092
	SA sociability investigation time	rmANOVA w/ *simple main effects*	Genotype	*F*(1,36) = .233	.632
			Stimulus	*F*(1,36) = 140.790	5.3E-14
			Sex	*F*(1,36) = 12.039	.001
			Genotype*Sex	*F*(1,36) = .059	.809
			Genotype*Stimulus	*F*(1,36) = .429	.517
			*Controls, stimulus*	*F*(1,36) = 78.384	1.4E-10
			*MPS IIIB, stimulus*	*F*(1,36) = 62.836	2.1E-9
	SA sociability preference index across time	rmANOVA	Genotype	*F*(1,36) = .143	.708
			Genotype*Minute	*F*(8.9,322.9) = 1.867	.056
	SA novelty preference index	ANOVA	Genotype	*F*(1,36) = .000	.991
			Sex	*F*(1,36) = .094	.761
			Genotype*Sex	*F*(1,36) = .301	.586
	SA social novelty investigation time	rmANOVA w/ *simple main effects*	Genotype	*F*(1,36) = 2.784	.104
			Stimulus	*F*(1,36) = 96.732	9.7E-12
			Sex	*F*(1,36) = 12.258	.001
			Genotype*Sex	*F*(1,36) = 2.390	.131
			Genotype*Stimulus	*F*(1,36) = 1.479	.232
			*Controls, stimulus*	*F*(1,36) = 37.144	5.2E-7
			*MPS IIIB, stimulus*	*F*(1,36) = 61.067	2.9E-9
	SA novelty preference index across time	rmANOVA	Genotype	*F*(1,36) = .058	.811
			Genotype*Minute	*F*(9,324) = 1.651	.100
	Tube Test percent wins	One-sample t-test (to 50%)	Female MPS IIIB	*t*(8) = -3.900	.005
			Male MPS IIIB	*t*(9) = -.919	.382
	RI bout with an attack	Fisher’s Exact Test	Genotype	OR = 1.25 [95% CI 1.045,1.495]	.024
	RI anogenital sniff duration	Mann–Whitney	Genotype	*U*(20) = 22	.035
	RI head sniff count	Mann–Whitney	Genotype	*U*(22) = 20	.023
6	Whole brain volume	ANOVA	Genotype	*F*(1,22) = 28.585	.00002
			Sex	*F*(1,22) = 4.137	.054
			Genotype*Sex	*F*(1,22) = .185	.671
	Body weight	ANOVA	Genotype	*F*(1,22) = 61.441	8.3E-8
			Sex	*F*(1,22) = 82.188	7.0E-9
			Genotype*Sex	*F*(1,22) = 3.582	.072
	Cerebellar volumetric ratio	ANOVA	Genotype	*F*(1,22) = 5.693	.026
			Sex	*F*(1,22) = 2.131	.158
			Genotype*Sex	*F*(1,22) = 1.144	.296
	Cerebellar apparent diffusion coefficient	ANOVA	Genotype	*F*(1,22) = 2.883	.104
			Sex	*F*(1,22) = .081	.779
			Genotype*Sex	*F*(1,22) = .782	.386
	Corpus callosal volumetric ratio	ANOVA	Genotype	*F*(1,22) = 10.219	.004
			Sex	*F*(1,22) = .016	.900
			Genotype*Sex	*F*(1,22) = .926	.346
	Corpus callosal fractional anisotropy	ANOVA	Genotype	*F*(1,22) = 1.394	.250
			Sex	*F*(1,22) = .010	.923
			Genotype*Sex	*F*(1,22) = 3.241	.086
	Cortical thickness	ANOVA	Genotype	*F*(1,22) = 1.399	.250
			Sex	*F*(1,22) = .113	.740
			Genotype*Sex	*F*(1,22) = .110	.743

^*^FDR q = *.1 adjusted observed p value*

^*simple main effects not significant*

## Results

### MPS IIIB mice exhibited disrupted early communicative behaviors with subtle yet persistent changes to the developmental gait trajectories

Speech delay is a first symptom of MPS IIIB making it an important phenotype to mark age at disease onset. While human language cannot be explored in mice, vocal communication behavior is conserved across taxa [26]. Mouse pups produce ultrasonic isolation calls as a way to attract the dam for maternal care [27], thus it is one of the earliest forms of social communication we can examine in mice. In addition, developing mice exhibit a trajectory of gait development that establishes ratios of stride components similar to that observed in humans [31], which can be leveraged to identify sensitive markers of motor impairment [32]. Here, we characterize the trajectories of call production (Fig. 1A) and gait in the MPS IIIB mice to identify early markers of disease.

MPS IIIB mice produced fewer calls overall compared to controls during the first two weeks of life (Fig. 1B-D; Table 2). Additional files show the full spectrogram for a representative MPS IIIB and control mouse (see Additional Files 1 and 2). This was not due to gross developmental delay as MPS IIIB mice body weights and latency to exhibit righting reflex at P14 were comparable to controls (Fig. 1E,F), and no differences were observed in number of mice that exhibited pinnae detachment at P6 (Controls, *n* = 27/27; MPS IIIB *n* = 17/17) or eye opening at P14 (Controls, 12.2% closed; MPS IIIB 12.5% closed). MPS IIIB mice also displayed significantly lower body temperatures relative to controls (Fig. 1G). Lower temperature is typically reported to increase the call rate to elicit maternal care and warmth [46]. However, we observe a reduction in call rate in the MPS IIIB mice, not an increase. To further confirm temperature was not modulating call rate (despite the misaligned direction of effect), we conducted a covariate model regressing out temperature and the reduced call rate was maintained (Fig. 1H), indicating the altered body temperature is not driving the call phenotype.

We next examined the spectrotemporal features of the calls to determine if physical anomalies, such as altered physiology of the lungs or larynx, may be heavily driving the reduction in call rate. We observed a reduction in the number of phrases of calls (chunks of call with less than 922.3 ms pauses between; Fig. 1I). In addition, MPS IIIB calls were shorter in average duration (Fig. 1J) with longer pauses within a phrase (Fig. 1K), yet were comparable in sound pressure level (Fig. 1L). Because the difference in number of calls and call duration are in the same direction with pause duration in the opposite direction, we can conclude that the reduced call rate is likely not driven by differences in respiration timing. The MPS IIIB calls are also lower in mean frequency pitch (Fig. 1M) with a narrower frequency range (Fig. 1N) and fewer percent of calls including a jump in frequency > 10 kHz (Fig. 1O). These pitch differences may result from a vulnerability in the laryngeal muscles. It is also possible that there is a higher-level disconnect with patterning of calls. Together, these data reveal an early marker of disease in the form of reduced call rate and altered call features that is independent of gross developmental delay.

In a separate cohort of mice, we examined the trajectories of gait development beginning on P21. Gait was measured across four juvenile time points and again in early adulthood (Fig. 2A). During development, MPS IIIB mice were able to run on forced gait apparatus just as well as the controls and did not require an increased number of attempts to run (Fig. 2B; Table 2). All mice increased body weight (Fig. 2C) and body length (Fig. 2D) across the window of assessment (P21—P30) with no significant differences between groups. Because of this drastic change in body size across time, a covariate model was used to regress out the influence of body size change on gait metrics[31]. All mice were tested at the same belt speed (20 cm/s) across development to allow for appropriate comparisons of gait metrics as speed is a robust influence on gait [47, 48]. After correcting for multiple comparisons across 44 gait metrics (Table 3), two remained significantly affected in the MPS IIIB mice. Specifically, the duration of braking for the forepaws was significantly increased (Fig. 2E) and the absolute paw angle of the hindpaws was significantly wider (Fig. 2F). We examined the mice again during adulthood just after P60 to determine if the developmental gait anomalies persisted or resolved with age. By adulthood, body length and body weight were significantly greater in the MPS IIIB mice compared to controls (Fig. 2G,H). At 30 cm/s, MPS IIIB mice were able to run on the apparatus as well as controls (Fig. 2I). Duration to brake for the forepaws was again increased in the MPS IIIB mice (Fig. 2J). Further gait anomalies were apparent at a higher belt speed of 40 cm/s in the adult mice. MPS IIIB mice took significantly more attempts to successfully run on the apparatus compared to controls (Fig. 2I). In addition, this higher speed induced an increased percent of time that both hindlimbs are in stance phase in the MPS IIIB mice (Fig. 2K) as well as decreased percent of stride in swing phase and increased percent of stride in stance phase for the hindlimbs (Fig. 2L). Together, these data indicate that gait was mildly disrupted in the MPS IIIB mice, which persisted into adulthood and worsened when challenged at a higher speed.

**Table 3:** Gait metrics examined during development and adulthood in MPS IIIB mice compared to control mice

Gait Metric	Observed *p* value			Gait Metric	Observed *p* value		
	Development	Adult 30 cm/s	Adult 40 cm/s		Development	Adult 30 cm/s	Adult 40 cm/s
AbsolutePawAngleFore	0.007	0.635	0.150	StanceFactorFore	0.062	0.014	0.617
AbsolutePawAngleHind	0.002	0.537	0.853	StanceFactorHind	0.122	0.257	0.442
BrakeFore	0.00005	0.0001	0.110	StanceFore	0.061	0.210	0.056
BrakeHind	0.381	0.859	0.043	StanceHind	0.474	0.033	0.018
GaitSymmetry	0.372	0.340	0.738	StanceWidthCVFore	0.197	0.175	0.169
MAXdA_dTFore	0.869	0.186	0.209	StanceWidthCVHind	0.096	0.857	0.587
MAXdA_dTHind	0.523	0.275	0.308	StanceWidthFore	0.292	0.221	0.355
OverlapDistanceHind	0.361	0.191	0.153	StanceWidthHind	0.444	0.084	0.302
PawAngleCVFore	0.168	0.187	0.515	StepAngleCVFore	0.323	0.670	0.644
PawAngleCVHind	0.893	0.780	0.351	StepAngleCVHind	0.128	0.227	0.859
PawPlacement PositioningHind	0.014	0.460	0.776	StepAngleFore	0.164	0.552	0.680
Pct_SharedStanceHind	0.167	0.029	0.001	StepAngleHind	0.498	0.468	0.282
Pct_StanceStrideFore	0.019	0.595	0.038	StrideFrequencyFore	0.600	0.326	0.259
Pct_StanceStrideHind	0.366	0.139	0.006	StrideFrequencyHind	0.903	0.507	0.275
Pct_SwingStrideFore	0.019	0.595	0.038	StrideLengthCVFore	0.506	0.712	0.582
Pct_SwingStrideHind	0.366	0.139	0.006	StrideLengthCVHind	0.676	0.516	0.212
PeakPawAreaCVFore	0.637	0.513	0.551	StrideLengthFore	0.609	0.396	0.234
PeakPawAreaCVHind	0.407	0.708	0.798	StrideLengthHind	0.808	0.343	0.300
PeakPawAreaFore	0.161	0.376	0.272	SwingDurationCVFore	0.393	0.304	0.762
PeakPawAreaHind	0.345	0.256	0.322	SwingDurationCVHind	0.807	0.115	0.634
PropelFore	0.135	0.013	0.917	SwingFore	0.205	0.701	0.713
PropelHind	0.957	0.109	0.079	SwingHind	0.671	0.811	0.285

### MPS IIIB mice showed significant weight gain during early adulthood with intact visual acuity and contrast

As an estimate of overall health and to identify possible early initiation of decline, the MPS IIIB mice in Cohort 3 were weighed throughout the testing period (Fig. 3A). As compared to controls, the MPS IIIB mice weighed significantly more starting in early adulthood (Fig. 3B; Table 2). This weight phenotype was observed in both females and males (Fig. 3C,D). Clinical evidence of visual acuity deficits have been revealed in some MPS IIIB patients [49]. To assess visual acuity and contrast thresholds, MPS IIIB mice and their littermate controls were tested using the Virtual Optomotor System (VOS). VOS allows for each eye to be tested independently through the innate rodent optomotor reflex. MPS IIIB mice showed comparable mean visual acuity (Fig. 3E) and visual contrast (Fig. 3F) to controls during late adolescence.

### MPS IIIB demonstrated reduced startle responses and cued fear responses

To understand if the MPS IIIB mice exhibit normal startle responses and ability to filter irrelevant stimuli through proper sensorimotor gating, we examined these mice in the Acoustic Startle/PPI task. The increased weight phenotype was confirmed by measurement on the force plate used in this apparatus (Fig. 4A), therefore the data were analyzed using an ANCOVA model with weight as a covariate and covariate adjusted means are presented in Fig. 4. MPS IIIB mice demonstrated a decrease in startle response to the 120 dB startle stimulus (Fig. 4B; Table 2), yet show comparable sensitivity to gradually increasing startle stimulus levels (Fig. 4C). A greater inhibition of startle following a much lower dB prepulse tone was also exhibited by the MPS IIIB mice (Fig. 4D). Comparison of responses during non-startle trials to those in startle trials revealed all mice were significantly startled (Fig. 4E). and thus did not exhibit issues hearing the 120 dB full spectrum white noise. These phenotypes were independent of weight and sex, and suggest by late adolescence, the MPS IIIB mice already exhibit features possibly related to reduced fear.

In early adulthood, we examined fear responses directly using the conditioned fear task (Fig. 4F). Fear responses, which have a component of anxiety, are active avoidance behaviors quantified in situations where a threat is imminent and well-defined [50]. We used the fear conditioning task to measure freezing behavior to evaluate associative fear memory in the MPS IIIB mice. MPS IIIB mice showed normal acquisition of fear memory (Fig. 4G), indicating no impaired learning association of a tone with a foot shock. Testing of contextual fear memory indicated MPS IIIB mice had comparable performance relative to control mice (Fig. 4H). Cued fear memory testing revealed a mild impairment in the MPS IIIB mice, which exhibited less freezing on the final day of test (Fig. 4I). Together these results indicate while fear acquisition and contextual memory are similar to controls, cued fear memory was mildly impaired in MPS IIIB mice. Together with the Acoustic Startle/PPI results, these findings may represent early features of reduced fear in this model.

### MPS IIIB exhibited intact social approach behavior with sex-specific differences in hierarchy behavior and aggression behavior

**Fig. 5:**
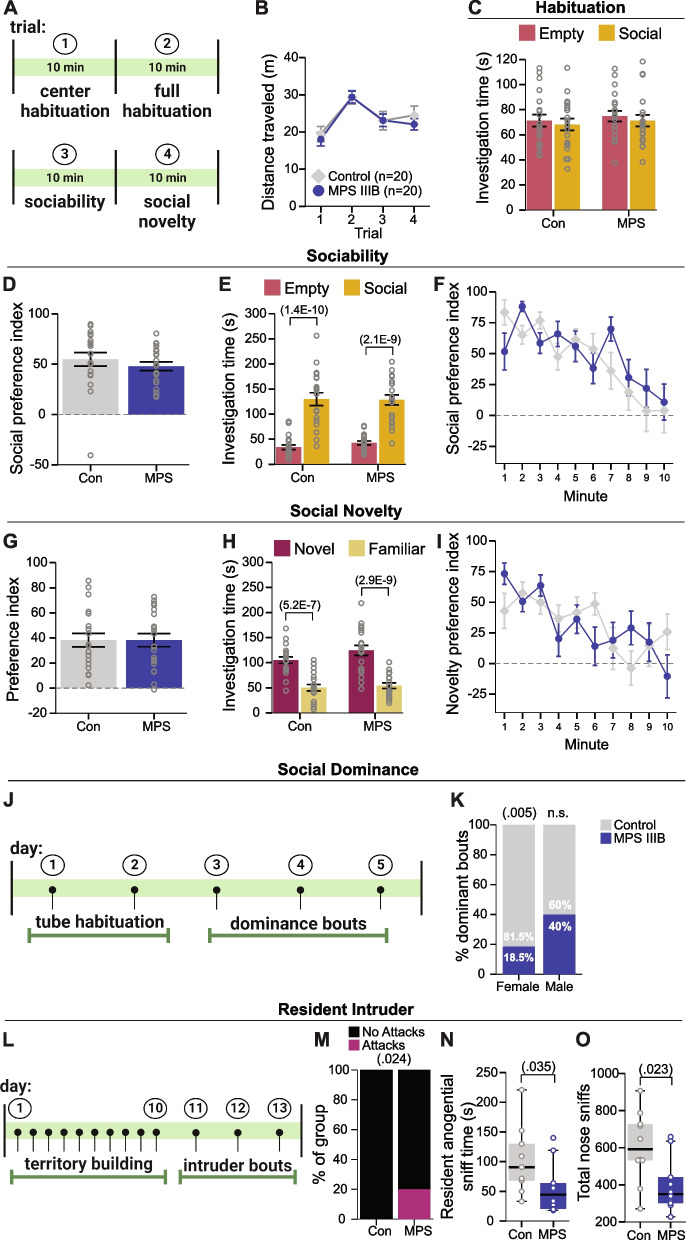
MPS IIIB mice exhibit normal social approach yet sex-specific altered dominance and aggressive behavior. **A** Schematic of social approach paradigm. **B** Distance traveled during all four trials of the social approach task. **C** Stimulus investigation time during full habituation. **D-F** Total social preference index (**D**), stimulus investigation time (**E**) and social preference index across the 10-min sociability trial (**F**). **G-I** Total novelty preference index (**G**), stimulus investigation time (**H**) and novelty preference index across the 10-min social novelty trial (**I**). **J** Schematic of social dominance tube test paradigm. **K** Percent of dominant bouts for controls and MPS IIIB mice split across males and females. **L** Schematic timeline of resident intruder paradigm. **M** Counts of intruder bouts with scramble attacks per group. **N** Duration of anogenital sniffing by the resident. **O** Number of total nose sniffs between residents and intruders. **B-I** Grouped data are presented as means ± SEM with individual data points presented as open circles.** K**,**M** Data are presented as counts. **N,O** Grouped data presented as boxplots with thick horizontal lines respective group medians, boxes 25th – 75th percentiles, and whiskers 1.5 × IQR. Individual data points are presented as open circles

To understand sociability and socially-mediated preference behavior in adolescent MPS IIIB mice, we tested these animals in the 3-chamber social approach task on P40 (Fig. 3A, 5A). No differences were observed between groups in the total distance traveled during all four trials (Fig. 5B; Table 2), nor were any side biases observed during the habituation trial (Fig. 5C). During the sociability trial examining time with a novel social partner or an empty withholding cup, MPS IIIB mice showed comparable social preference (Fig. 5D) as well as total time spent interacting with the social stimulus comparable to controls (Fig. 5E). We also examined the social preference index across each minute of the test to see if a previous finding of increased preference during minute 1 in the MPS IIIB mice was replicated [18]. We found a non-significant decrease in social preference index during minute 1 for the MPS IIIB mice (Fig. 5F). During the social novelty trial examining the innate preference for a novel versus familiar social partner, the MPS IIIB mice exhibited comparable novelty preference (Fig. 5G) as well as time investigating the novel partner mouse during the full 10 min trial (Fig. 5H). We also observed a non-significant increase in novelty preference during the first minute of this trial for the MPS IIIB mice (Fig. 5I).

The tube test of social dominance was used to assess the social hierarchy behavior of MPS IIIB mice when paired with sex-matched controls (Fig. 5J). While males show no significant effect of genotype, female MPS IIIB mice were significantly more submissive to control females, demonstrated by only 18.5% of dominant bouts, which was significantly less than chance (50%; Fig. 5K). Finally, the resident intruder paradigm (Fig. 5L) was used to determine if male MPS IIIB mice exhibit increased aggressive behavior. Control mice failed to engage in any scramble attacks with the intruder mouse. However, MPS IIIB mice engaged in scramble attacks with the intruders during 20% of their bouts (Fig. 5M). Examination of non-aggressive social behaviors during this task revealed that MPS IIIB males spent less time engaged in anogenital sniffing of the intruder (Fig. 5N) and fewer nose to nose sniffing bouts with the intruder (Fig. 5O). Together, these data indicate female MPS IIIB mice may be more submissive while males are more aggressive compared to controls.

### MPS IIIB mice have larger whole-brain and regional volumes compared to controls with intact tissue integrity

**Fig. 6:**
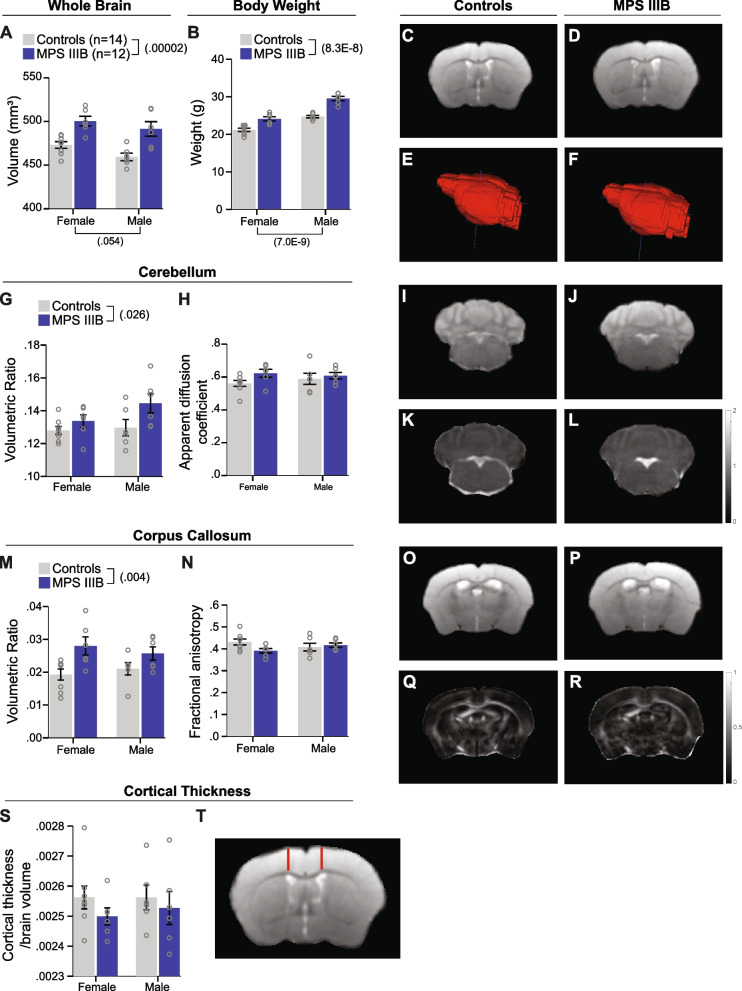
MPS IIIB mice have larger whole brain and regional volumes with intact tissue integrity. **A** Whole brain volumes measured by MRI. **B** Body weight measured prior to MRI. **C-F** T2-weighted image and whole brain volume of representative control (**C**,**E**) and MPS IIIB mouse brains (**D**,**F**). **G** Ratio of cerebellar volume to whole brain volume. **H** Mean apparent diffusion coefficient for the cerebellum. **I**,**K** Representative T2-weighted image (**I**) and ADC map (**K**) of control mouse cerebellum (ADC unit, µm^2^/ms). **J**,**L** Representative T2-weighted image (**J**) and ADC map (**L**) of MPS IIIB mouse cerebellum (ADC unit, µm^2^/ms). **M** Ratio of corpus callosal volume to whole brain volume. **N** Fractional anisotropy of the corpus callosum as measured by DTI.** O**,**Q** Representative T2-weighted image (**O**) and FA map (**Q**) of control mouse ­­corpus callosum. **P**,**R** Representative T2-weighted image (**P**) and FA map (**R**) of MPS IIIB mouse ­­corpus callosum. **S** Ratio of cortical thickness measurement to whole brain volume. **T** Representative schematic of cortical thickness measurements indicated by the red line. Data are presented as means ± SEM with individual data points presented as open circles

To determine if the MPS IIIB model recapitulates cerebellar atrophy and white-matter thinning reported in patients [4, 5], we performed both structural MR imaging and DTI experiments in cohort 3 animals following completion of behavioral testing at approximately four months of age. The MPS IIIB mice had significantly larger whole-brain volumes at this age compared to their control littermates (Fig. 6A,C-F; Table 2). While the MPS IIIB mice also exhibited larger body weights compared to controls (Fig. 6B), we observed differences in the magnitudes of volumetric differences (females = 5.8%, males = 7.0%) and body-weight difference (females = 13.8%, males = 19.4%) between genotypes. This difference in magnitude of effects suggests the brain size and body size differences may be independent of each other. However, a longitudinal assessment starting from a young age would be needed to confirm this.

Structural regional assessments were conducted by comparing ratios of regional gray matter volumes to whole brain volumes. In the cerebellum, this volumetric ratio was higher in the MPS IIIB mice compared with controls (Fig. 6G,I,J). The structural volumetric ratio also was higher in MPS IIIB mice for corpus callosal volumes (Fig. 6 M,O,P) Next, examining tissue integrity by DTI, the apparent diffusion coefficient for the cerebellum was unchanged between genotypes (Fig. 6H,K,L), as was the fractional anisotropy for the corpus callosum white-matter tract (Fig. 6N,Q,R). Finally, we examined cortical thickness using the T2-weighted images normalized to brain volume. We found comparable cortical thickness ratios for MPS IIIB and control mice (Fig. 6S-U).

To determine if the regional volume differences could be related to behavioral phenotypes, we conducted correlations for acoustic startle/PPI, contextual fear conditioning, and social dominance performance for the subset of cohort 3 mice that received both behavioral phenotyping and imaging. We found a low, negative correlation between the volumetric ratio for the corpus callosum and percent of dominance bouts (Table 4). No other significant relationships between behavioral performance and regional volumetric ratios were detected.

**Table 4:** Correlations between regional volumetric ratios and behavioral metrics with differential phenotypes across genotypes

Behavioral Outcome	Metric	Cerebellar Ratio	Callosal Ratio
Average startle response normalized for weight	Pearson Correlation	.195	-.224
	Sig. (2-tailed)	.341	.272
	N	26	26
Startle inhibition to 16 dB prepulse normalized for weight	Pearson Correlation	-.187	.114
	Sig. (2-tailed)	.359	.580
	N	26	26
Average % freezing during cued fear memory testing	Pearson Correlation	.197	-.099
	Sig. (2-tailed)	.336	.630
	N	26	26
Percent bouts won during social dominance tube test	Pearson Correlation	-.121	-.467^*^
	Sig. (2-tailed)	.563	.019
	N	25	25

## Discussion

Characterization of early-onset and clinically-relevant novel phenotypes in the MPS IIIB mouse model will help to identify more sensitive markers to use in evaluation of potential therapies for Sanfilippo Syndrome B. Previous early markers include enhanced degradation of synaptophysin and priming of microglia by postnatal day 10 [51, 52], which require histopathological or biochemical analysis of collected tissue. In this report, we provide evidence of neurobehavioral features in this model suggestive of disease during the first two weeks postnatal as well as evidence of phenotypes during late adolescence and early adulthood.

To our knowledge, this is the first study to examine the trajectory of early communication in the preclinical MPS IIIB model to better understand if this model recapitulates aspects of clinical speech delay [2]. While human language cannot be explored in mice, vocal communication behavior is conserved across taxa [26]. USVs produced by the mouse pup is one of the earliest forms of social communication we can examine in the mouse, and these isolation calls elicit maternal care from the dam [27]. Late in the first week of life and into the second, MPS IIIB mice produced significantly fewer USVs compared to controls as well as alterations to temporal and spectral USV features. It is unknown if the rate of infant crying is diminished in the Sanfillippo population, which would be an interesting future clinical investigation. However, our findings suggest that communication circuits are already perturbed early in development, identifying a very early marker of disease. These very early phenotypes might be leveraged in future treatment studies as markers of in utero interventions, such as enzyme replacement therapy [53, 54].

Along with USV reductions, we also noted a significant decrease in body temperature in MPS IIIB pups compared to controls. Temperature can be a significant modulator of USV rate with colder body temperatures increasing USV rates to elicit maternal care and warmth [46]. However, the temperature effects noted here are in the wrong direction, thus, temperature differences are not driving our USV phenotype. By late adolescence and early adulthood through four months of age, MPS IIIB mice exhibited significantly higher body weights compared to controls, which fall within the expected variability as reported by the Jackson Laboratory (https://www.jax.org/news-and-insights/jax-blog/2020/march/variability-in-aged-mice). However, our results are in contrast to a recent study reporting no differences in weight in MPS IIIB mice compared to controls during the same time period [55]. Hyperactivity has been repeatedly demonstrated in this mouse model [16, 17, 56]. Further, MPS IIIB do not show substantial physical decline and movement impairments until seven to nine months of life, immediately preceding death [57]. Thus it is unlikely the weight gain is a result of inactivity, however, it may reflect other metabolic or energy regulation issues. Previous work on multiple preclinical lysosomal storage disease models, including MPS IIIB, found adipose deficiencies that correlated with reduced leptin levels indicative of altered energy balance [58]. In addition, a recent report found neuronal damage in MPS IIIB mouse brains at five months of age was most notable in thalamic and hypothalamic regions [55]. It is also possible this weight gain is a result of storage material build up. Together, these findings suggest a possible hypothalamic dysfunction or energy regulation disruption, possibly due to the build up of storage material, that is present at very early ages.

In addition, when MR imaged at four months of age, MPS IIIB whole brain volumes as well as cerebellar and callosal volumetric ratios were significantly larger than controls. These were surprising results due to the reports of cerebellar atrophy and white matter abnormalities that accompany behavioral symptomatology [6, 7]. We performed imaging at an age prior to substantial physical decline and movement impairments, which occur around seven to nine months of life, immediately preceding death [57]. MR imaging at a later age nearer this period of decline may reveal features more representative of those observed clinically. Macrocephaly has been reported in a subset of Sanfilippo B patients, although this was coupled with MRI evidence of cortical atrophy in a higher portion of patients [59], which we did not observe here. Our imaging data did not correlate well with behavioral findings. This is consistent with previous study that found the rate of MRI changes were unrelated to the severity of clinical features [6]. However, more work is needed to understand if this is a recapitulation of clinical presentation. Further, it is unclear how larger brain volumes might relate to body size, as we were unable to obtain physical measurements such as femur length in cohort 3. Build up of storage materials occurs in several organ systems including the heart, spinal cord, and brain in both the murine and canine models [10, 60–62]. Thus, the larger brain volumes may reflect storage buildup. Further studies coupling structural MRI with quantification of disease-specific heparan sulfate and other storage materials is needed to test this hypothesis.

Loss of acquired functions, including motor functions, is a hallmark of MPS IIIB [63]. While speech decline presents early and frequently, loss of motor skills, including gait, occurs less severely and less frequently. Our data are consistent with these clinical findings. Specifically, we found largely intact developmental trajectories of gait in MPS IIIB mice with abnormalities not present until early adulthood, with the exception of brake duration in the forepaws and absolute hindpaw angle. These two features indicate that the MPS IIIB mice do not increase their speed with which they brake during the stance phase of a stride, a feature of gait maturation we observed previously [31]. We also observed a similar difference from controls for brake duration in two other forms of developmental disabilities genetic risk, specifically a mouse model of Neurofibromatosis Type 1 mutation and the complete deletion mouse model of WIlliams Syndrome [32]. In adulthood, the more robust differences in gait performance were not apparent unless the mice were challenged with a higher belt speed. Previously, we found MPS IIIB mice at 16 weeks of age performed comparably to controls in a standard accelerating rotarod procedure [18], while longitudinal work using a more challenging rocking rotarod observed motor performance declines in the MPS IIIB mice after 200 days of age [16, 17]. Together with our data here, these findings suggest more challenging procedures may be necessary to demonstrate the MPS IIIB motor phenotypes. In addition, gait may be a more sensitive motor domain than rotarod coordination measures to discern earlier motor decline in this model. Joint stiffness and bone abnormalities have been reported clinically [64, 65]. Thus it will be important for future work to investigate possible contributions of joint pathology and bone abnormalities to gait abilities in this mouse model.

Reduced fear is a later onset feature of MPS IIIB [15–19]. We observed mild impairment to cued fear memory in the fear conditioning task in young adult mice (P67). Previous work by others using this model demonstrated intact cued memory around the age studied here (2.5 months) with a deficit presenting by 4.5 months [19]. Li et al., presented the tone once for 10 s accompanied by an 0.5 mA aversive foot shock for the final 2 s, while we presented the tone three times for 20 s with a 1.0 mA aversive foot shock during the final 2 s. Thus, it may be the differences in our procedure allowed us to observe the mild cued fear impairment at this earlier age.

Startle responses, such as those measured in our Acoustic Startle/PPI task, can be potentiated by fear stimuli, such that increasing the fear response increases the amplitude of the acoustic startle response [66]. In addition, startle responses have been positively correlated with anxiety levels [67]. Here, we observed reduced acoustic startle responses in the MPS IIIB mice at P53. This may be an early marker of the clinically-relevant reduced fear phenotype in this model. Further, this may reflect previously described cholinergic disruptions in this model; specifically, decreased acetylcholinesterase activity [18]. Cholinergic neurons of the pedunculopontine tegmentum modulate startle responses [68], thus the reduced startle observed here may be driven by underlying emerging cholinergic pathway abnormalities. We also observed an accompanying increase in inhibition of startle following a prepulse in MPS IIIB mice. Increases in this type of inhibition have been associated with reduced glutamatergic and dopaminergic transmission and increased serotonergic transmission [69–71]. Thus, it is plausible these changes in startle inhibition reflect underlying changes to neural activity in the MPS IIIB brain at this early age. Future work in this area will be important for identifying the aberrant circuitry mediating this phenotype.

To determine if this model recapitulates social disruptions reported in clinical populations, we examined social approach, social hierarchy ranks, and interactions in a resident intruder paradigm. Consistent with previous work conducted at 16 weeks of age, we did not see robust changes to sociability in the social approach task [18]. In contrast to that work, MPS IIIB mice here failed to show an initial dip in social preference index. We tested the mice at P40, an age within the peak social reward learning critical period [72] when mice find social interaction highly rewarding. Thus, the increased rewarding value of social interactions at this age may be overcoming any effects of MPS IIIB pathology on social neural circuitry. This hypothesis is supported by observations in another IDD-related genetic risk model, *Myt1l* haploinsufficiency. Social behavior disruptions in the *Myt1l* model were observed during adulthood (2–3 months of age) but not during an age in the peak social reward period (P23) [73]. During early adulthood, the MPS IIIB mice showed sex-specific dominance effects. Specifically, female MPS IIIB mice were submissive to controls while the males' social hierarchy behavior was unchanged. Finally, when tested later in adulthood for direct social interactive behaviors, the male MPS IIIB mice demonstrated more aggressive behavior towards the intruders compared to control males, with less non-aggressive social sniffing, thus recapitulating the aggression reported in Sanfilippo B patients.

## Conclusions

In the MPS IIIB mouse model, we observed neurobehavioral abnormalities as early as end of first week postnatal, as well as novel phenotypes recapitulating clinically-relevant features including reduced fear, gait disruption, and social behavior changes by late adolescence and early adulthood, respectively. In addition, physical changes and larger brain volumes were observed, potentially reflecting hypothalamic or metabolic dysfunctions and buildup of storage material, respectively, that necessitate further examination. Thus, we identified novel and early physiological and behavioral markers of disease in the MPS IIIB model that will serve as treatment outcome targets for future preclinical evaluations of potential therapeutics, such as gene or enzyme replacement therapies through possible in utero or very early postnatal life interventions [53, 54].

### Supplementary Information

**Additional file 1. **Full spectrogram of ultrasonic vocalization recording for a representative MPS IIIB mouse.

**Additional file 2. **Full spectrogram of ultrasonic vocalization recording for a representative control mouse.

## Data Availability

All data and detailed protocols are available upon reasonable request.

## Abbreviations

MPS: Mucopolysaccharidosis
USV: Ultrasonic vocalization
NAGLU: Alpha-N-acetylglucosaminidase
VOS: Virtual optomotor system
CW: Clockwise
CCW: Counterclockwise
PPI: Prepulse inhibition
MRI: Magnetic resonance imaging
DTI: Diffusion tensor imaging
