# Supplementary figures and images for: Characterization of early markers of disease in the mouse model of mucopolysaccharidosis IIIB
Source: J Neurodev Disord. 2024 Apr 17;16:16. doi: 10.1186/s11689-024-09534-z (PMC11022360; doi:10.1186/s11689-024-09534-z)

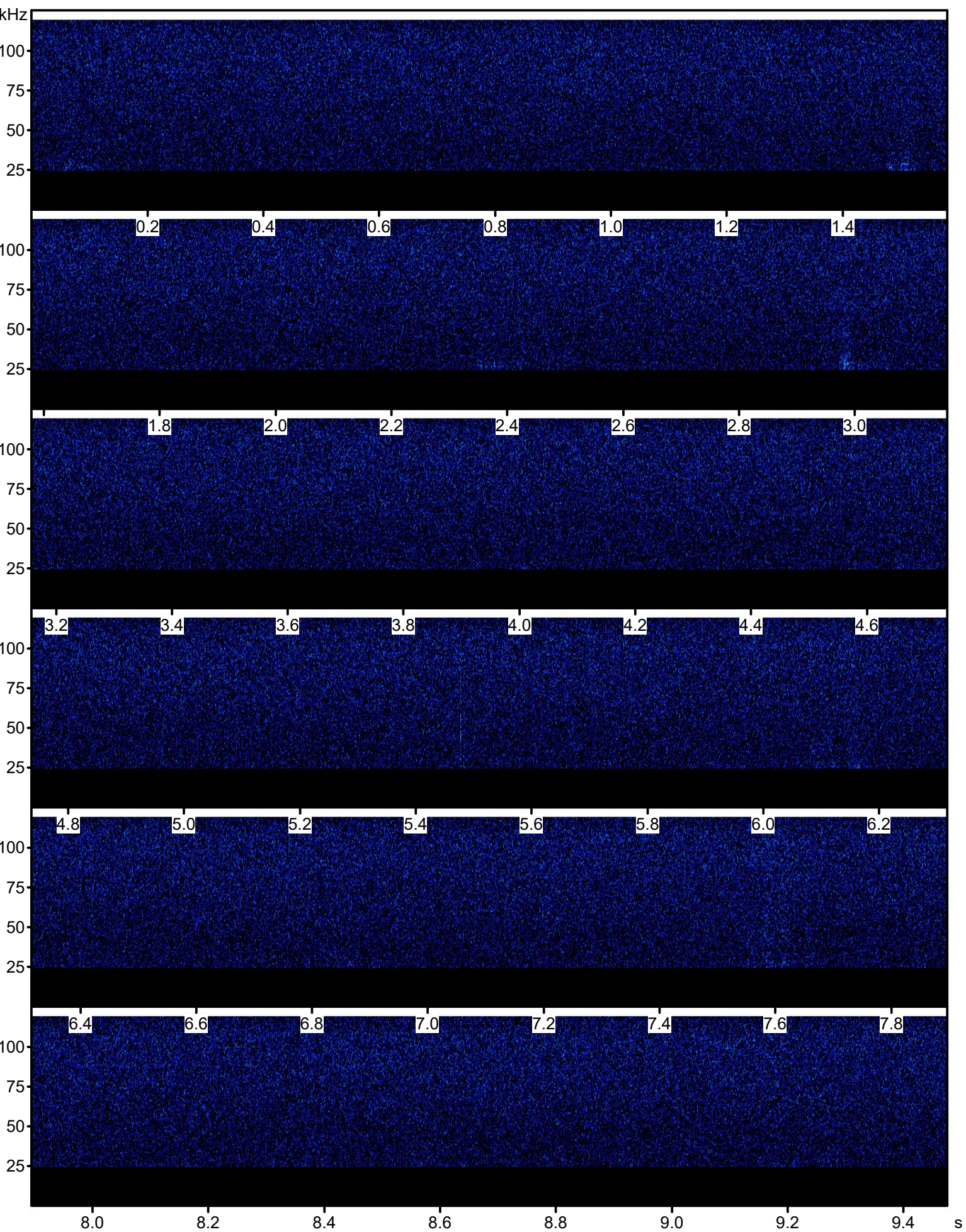

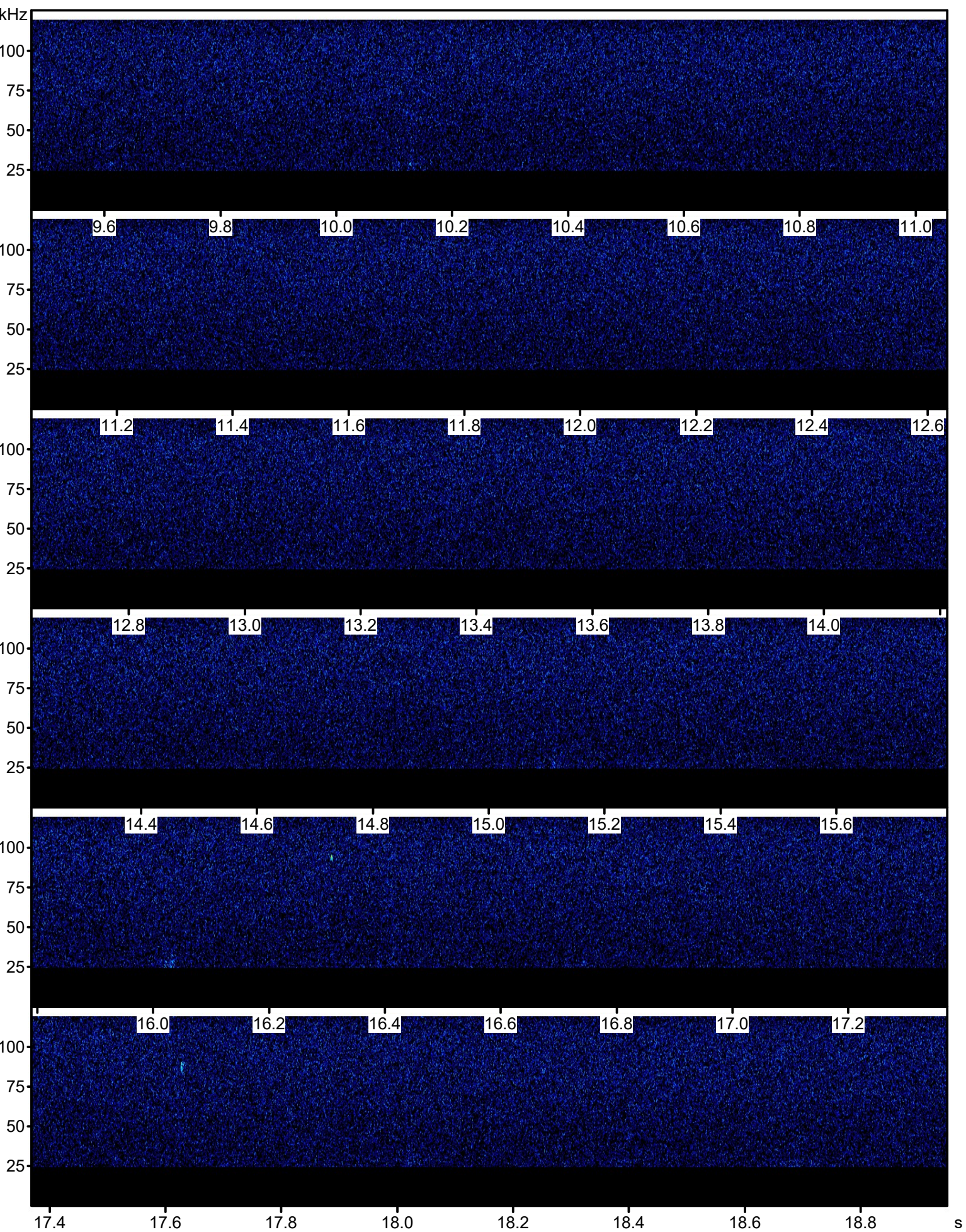

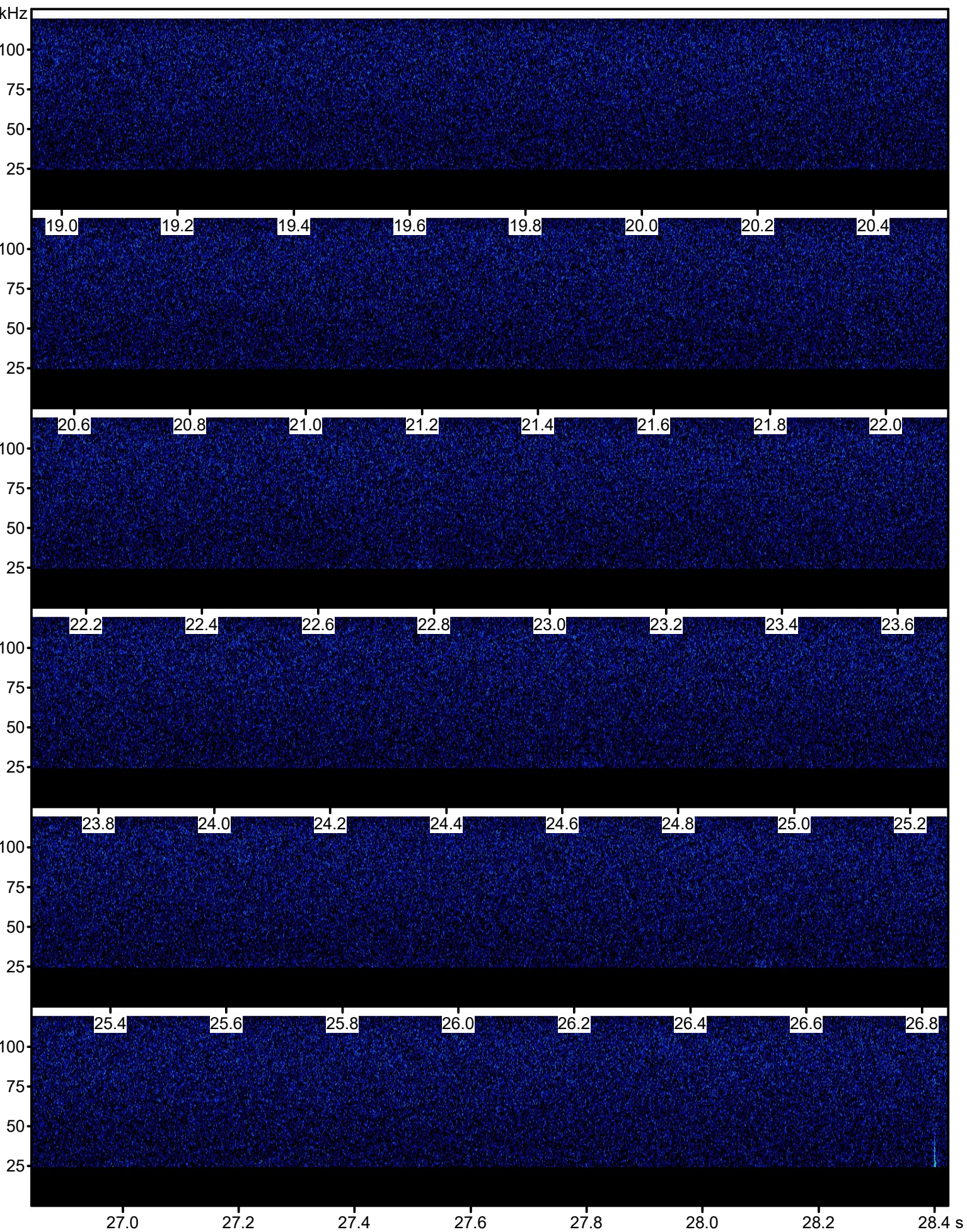

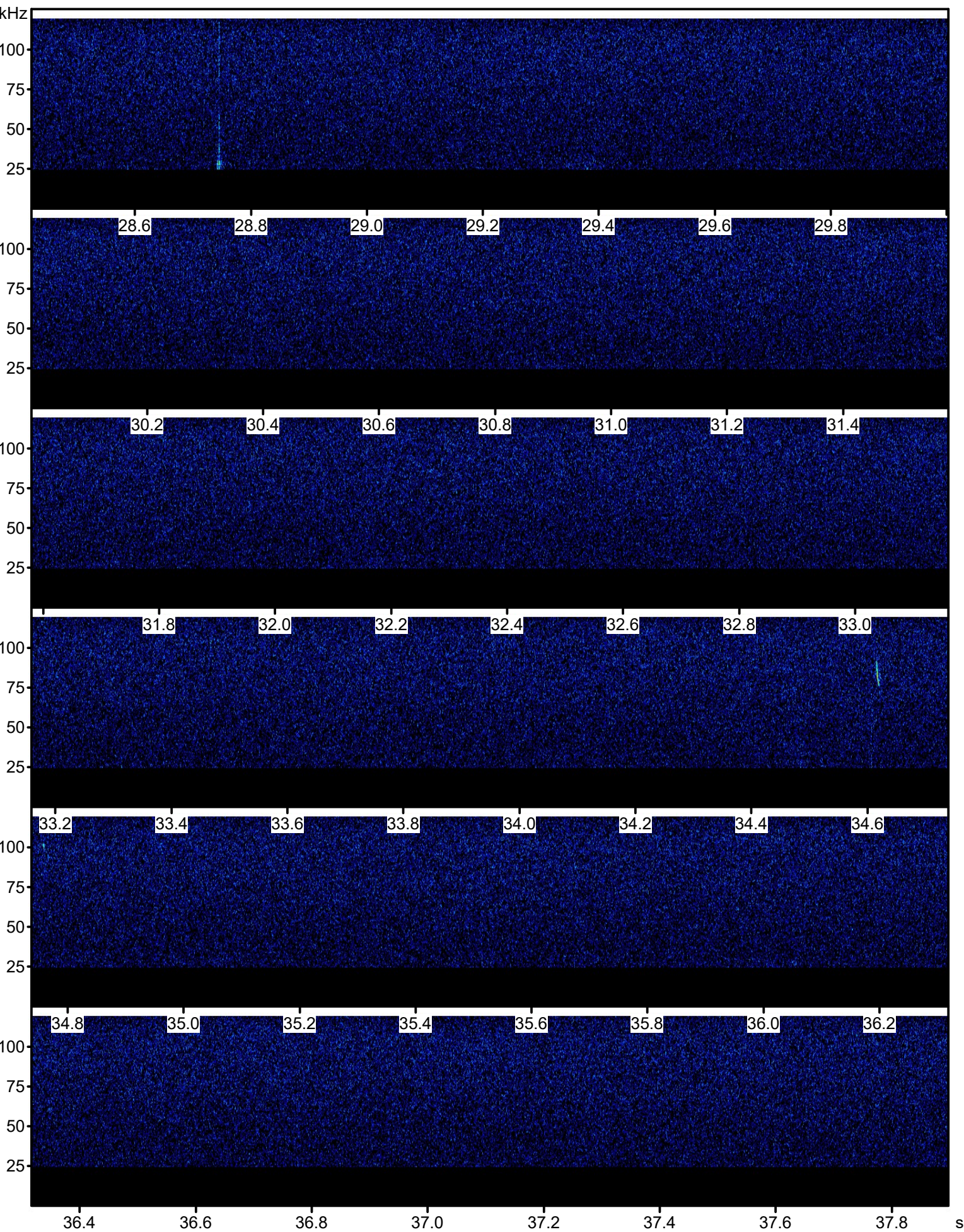

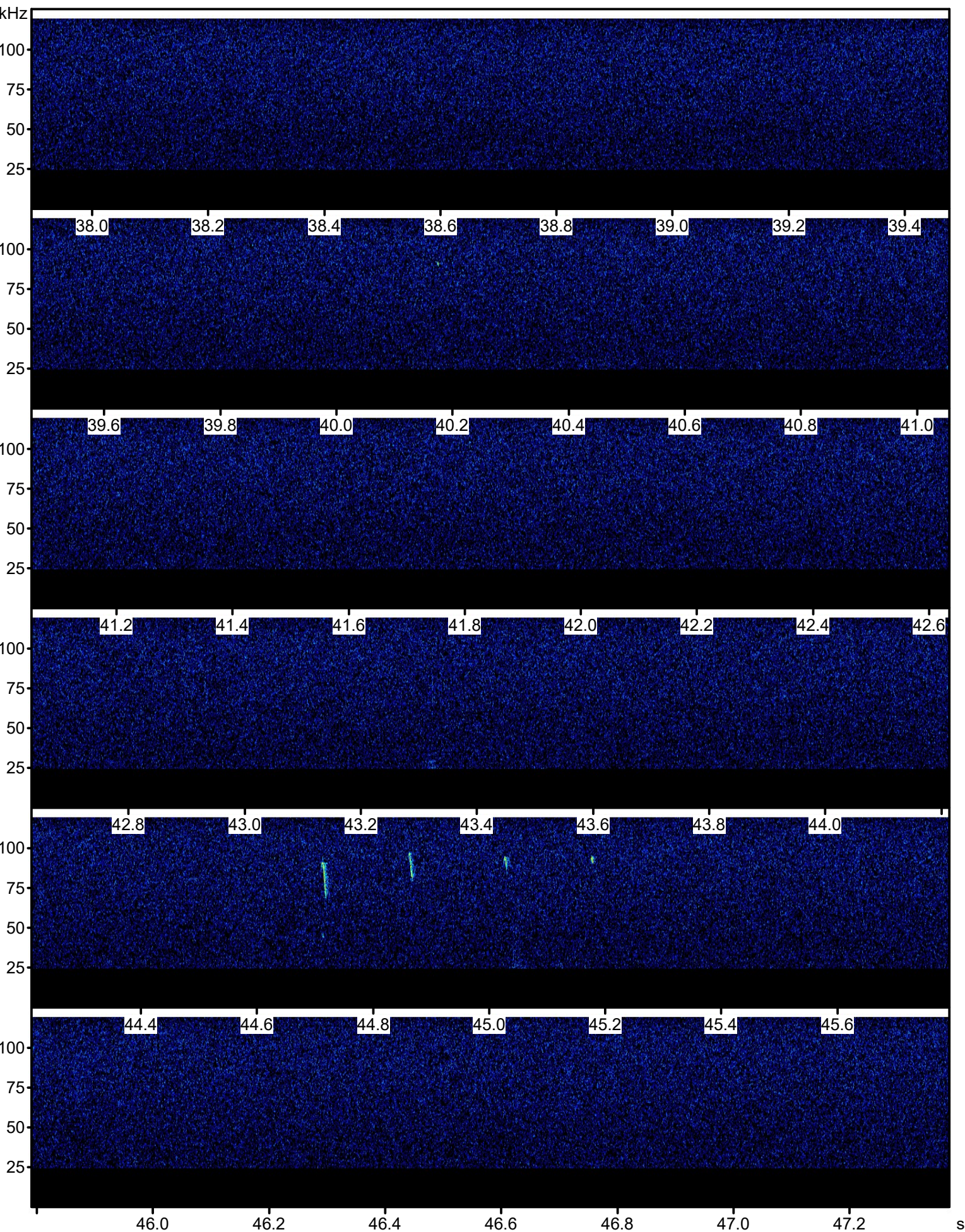

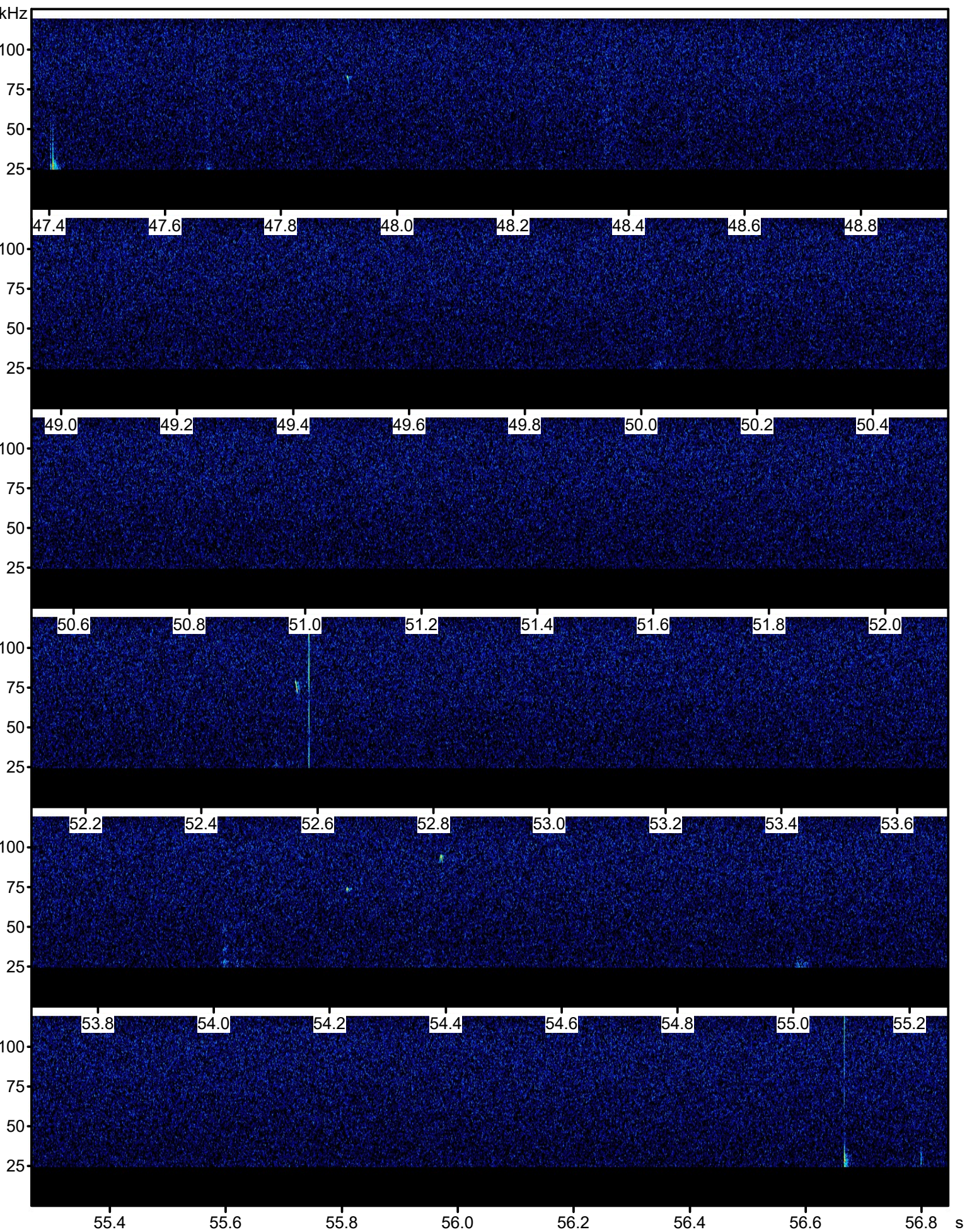

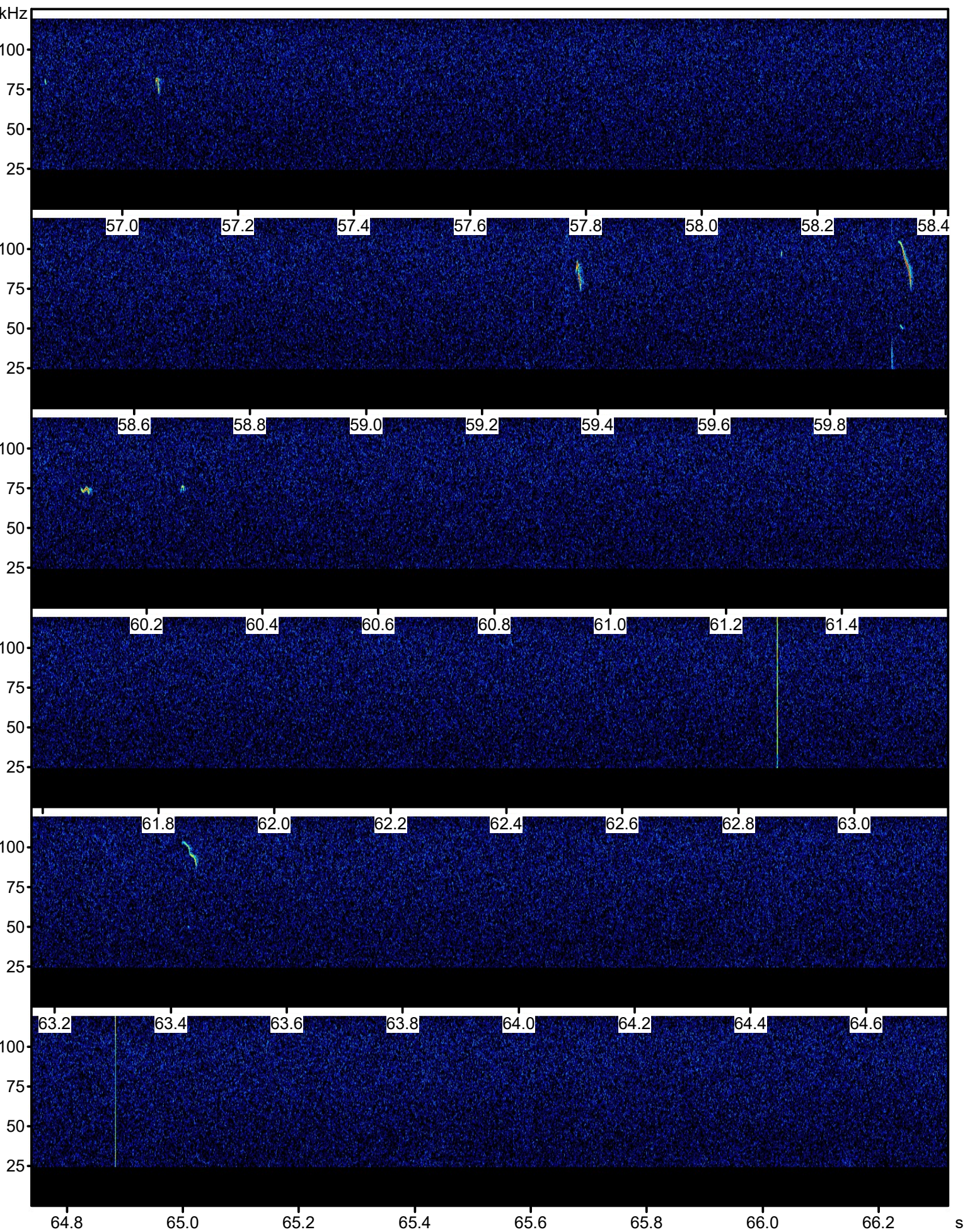

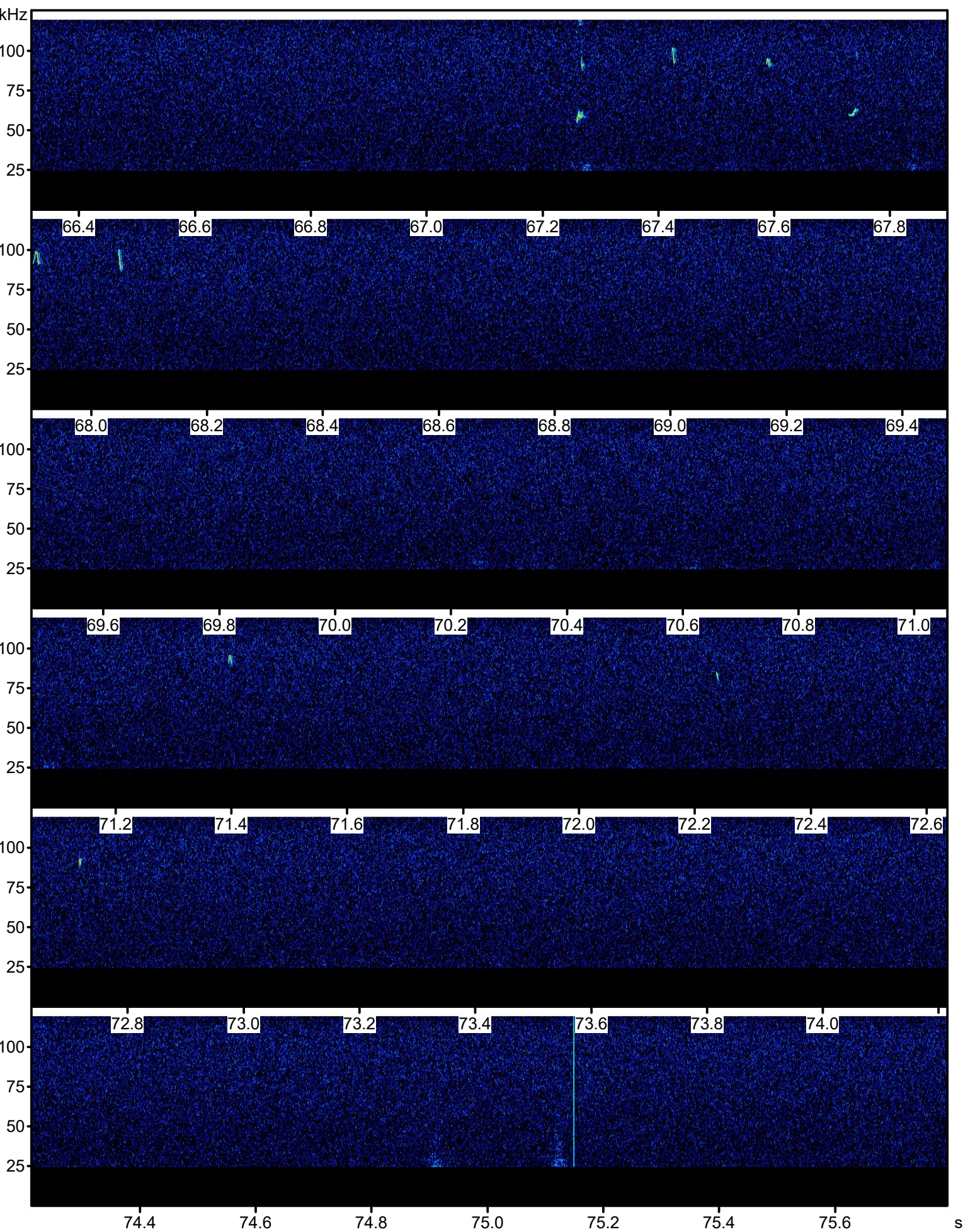

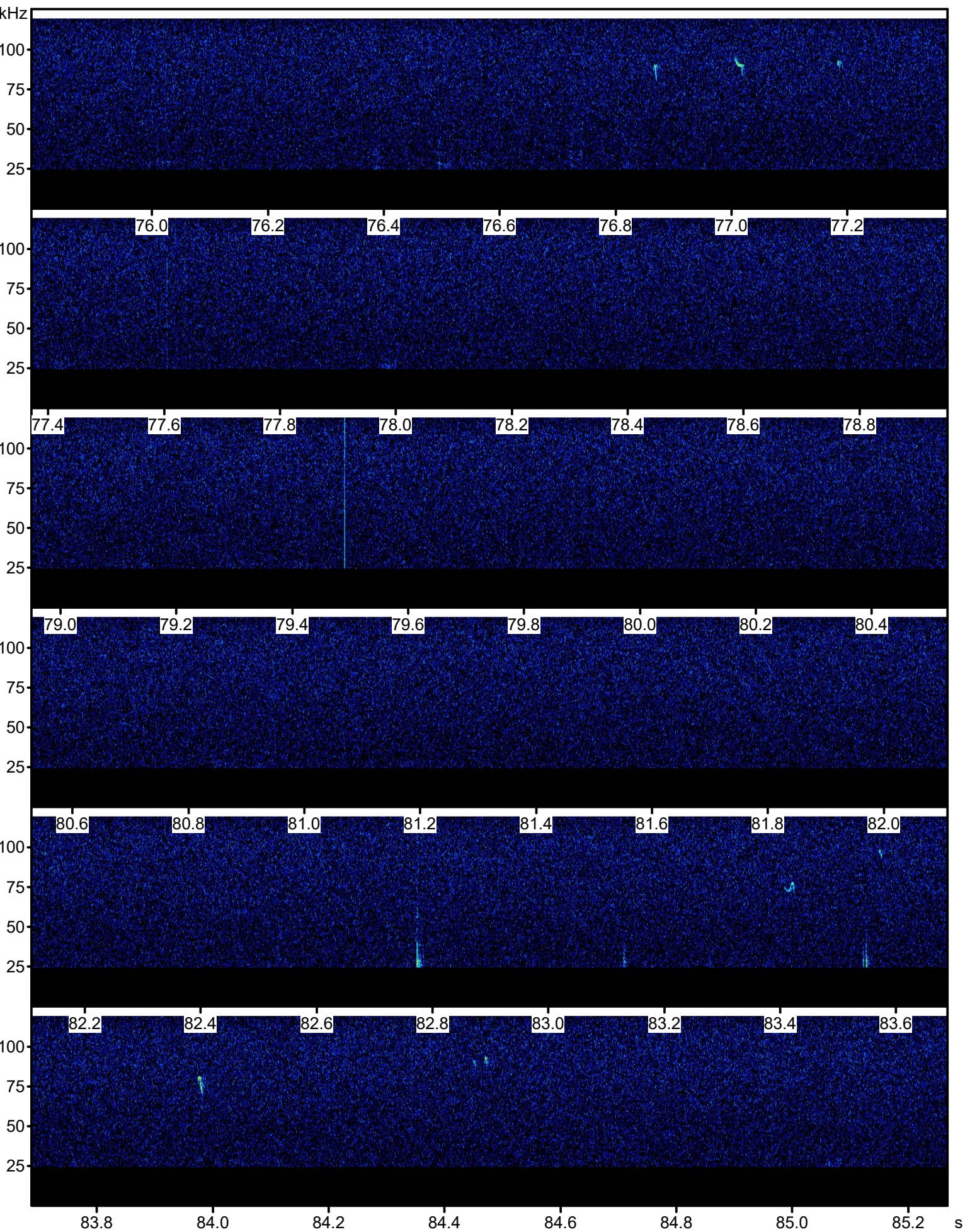

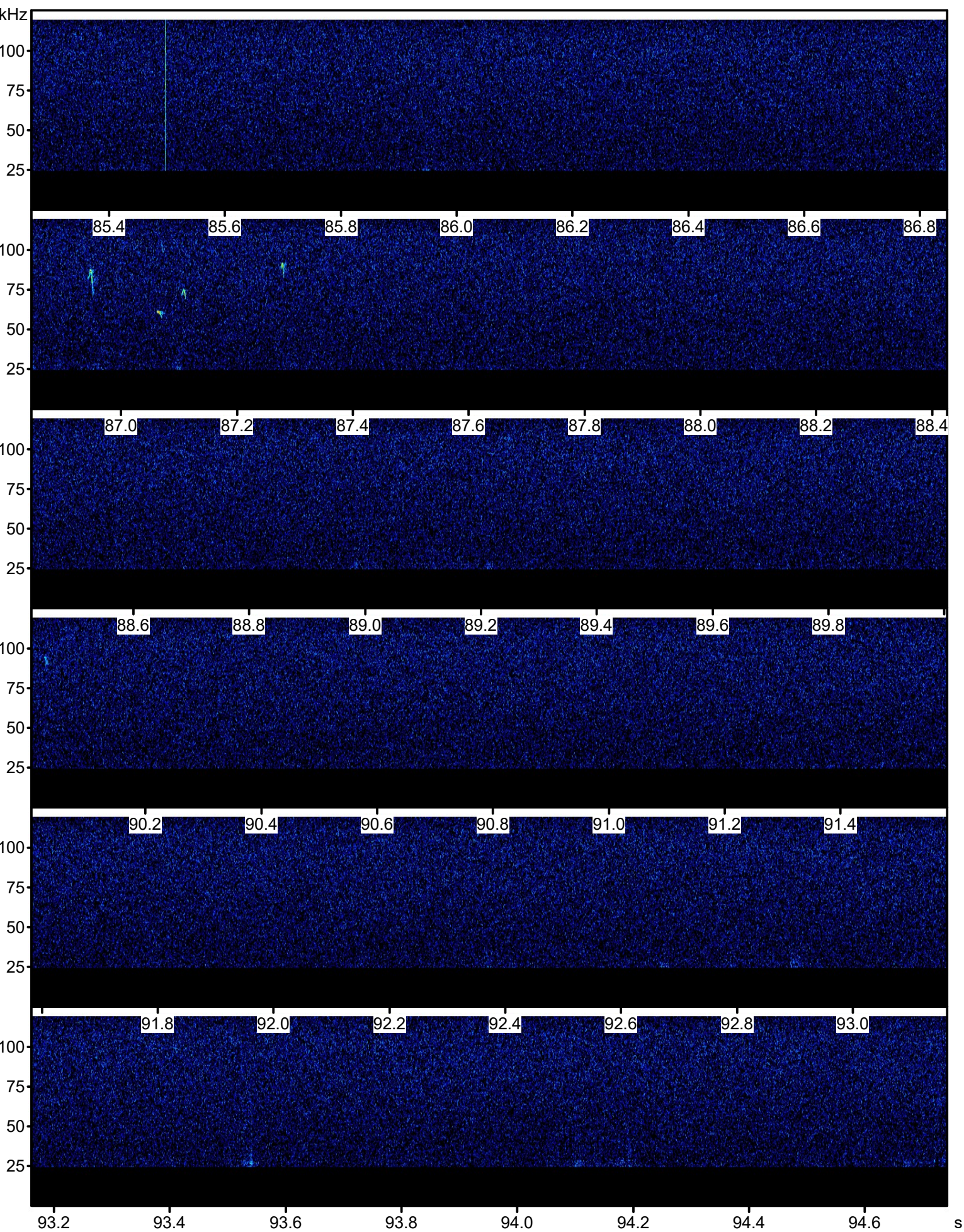

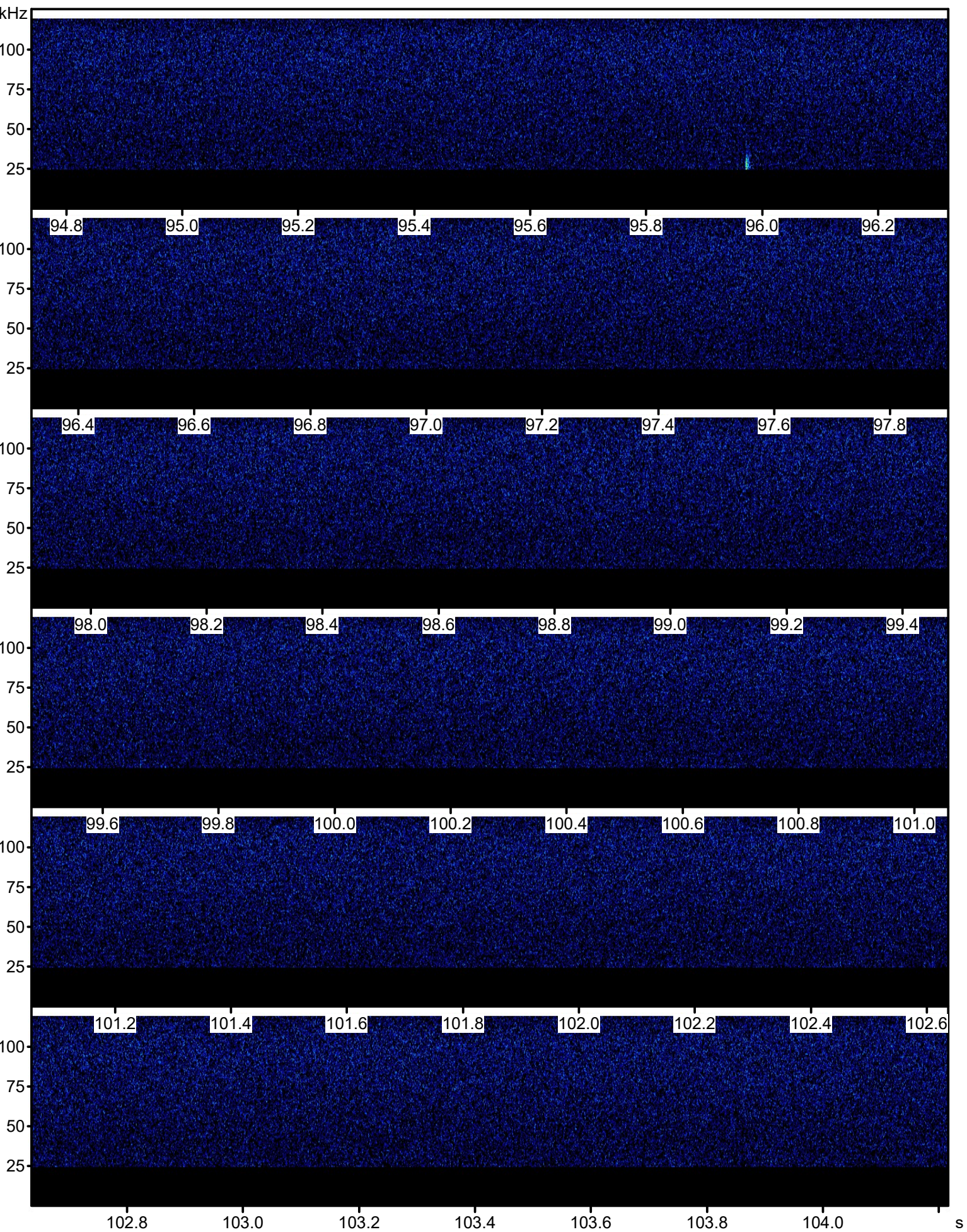

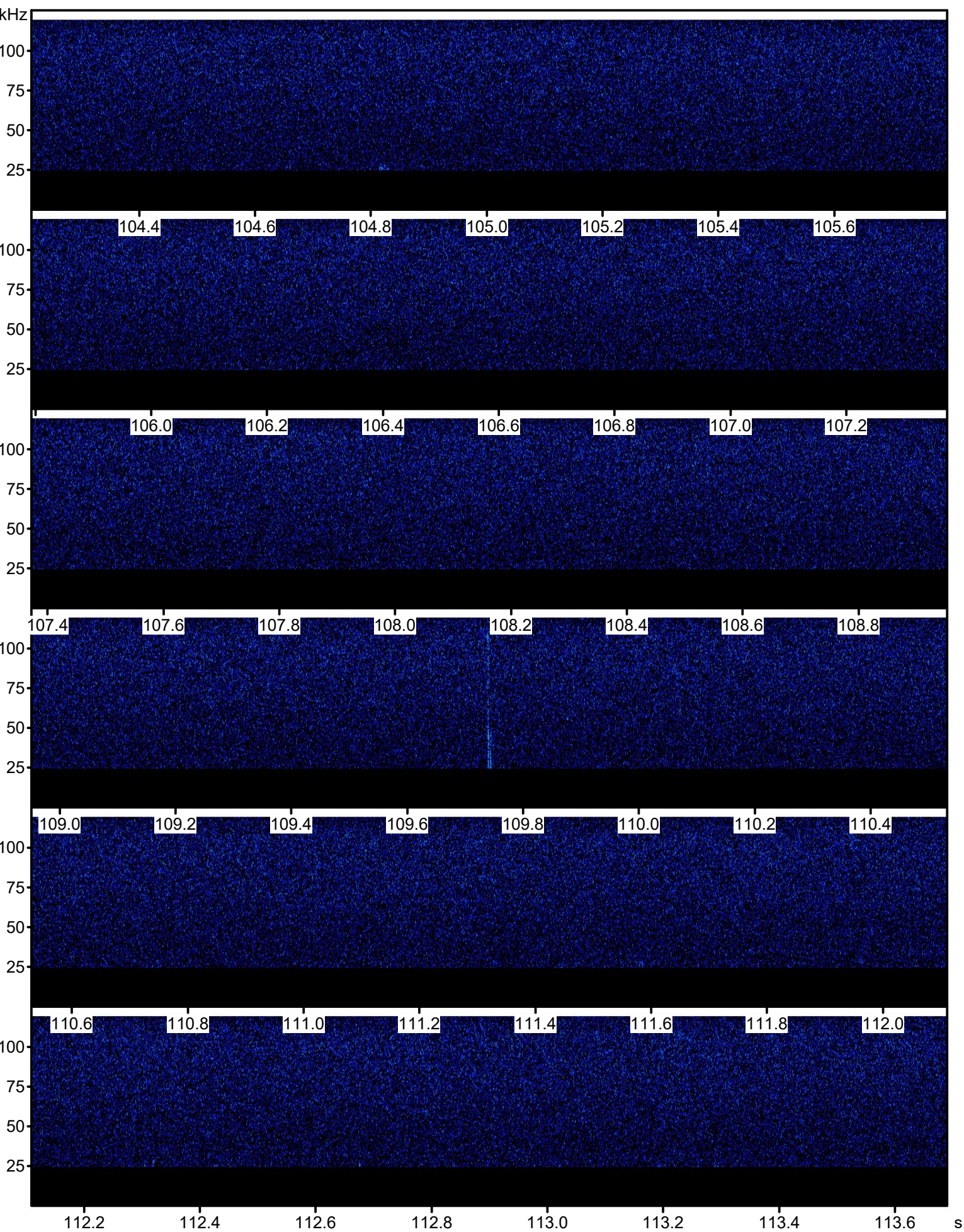

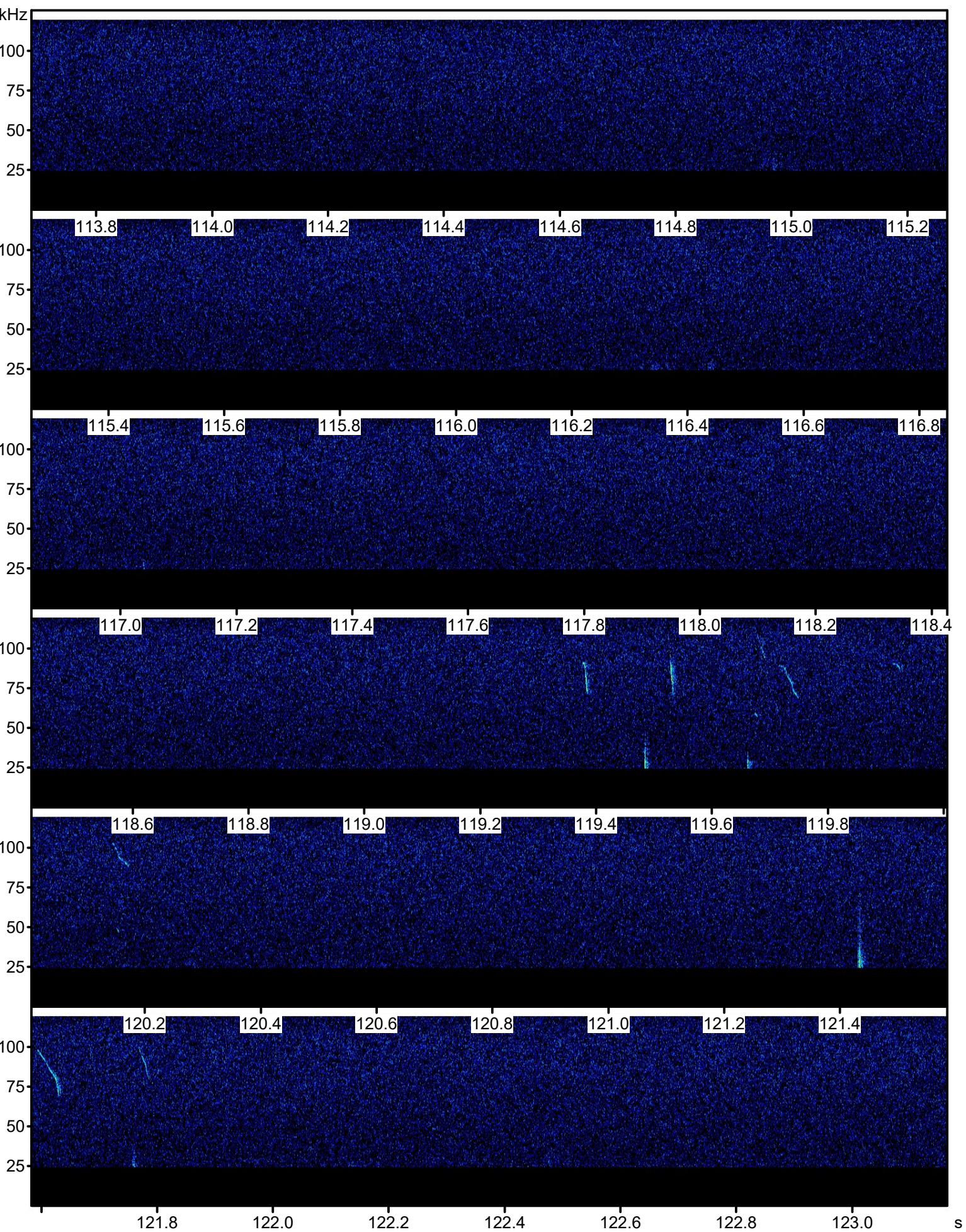

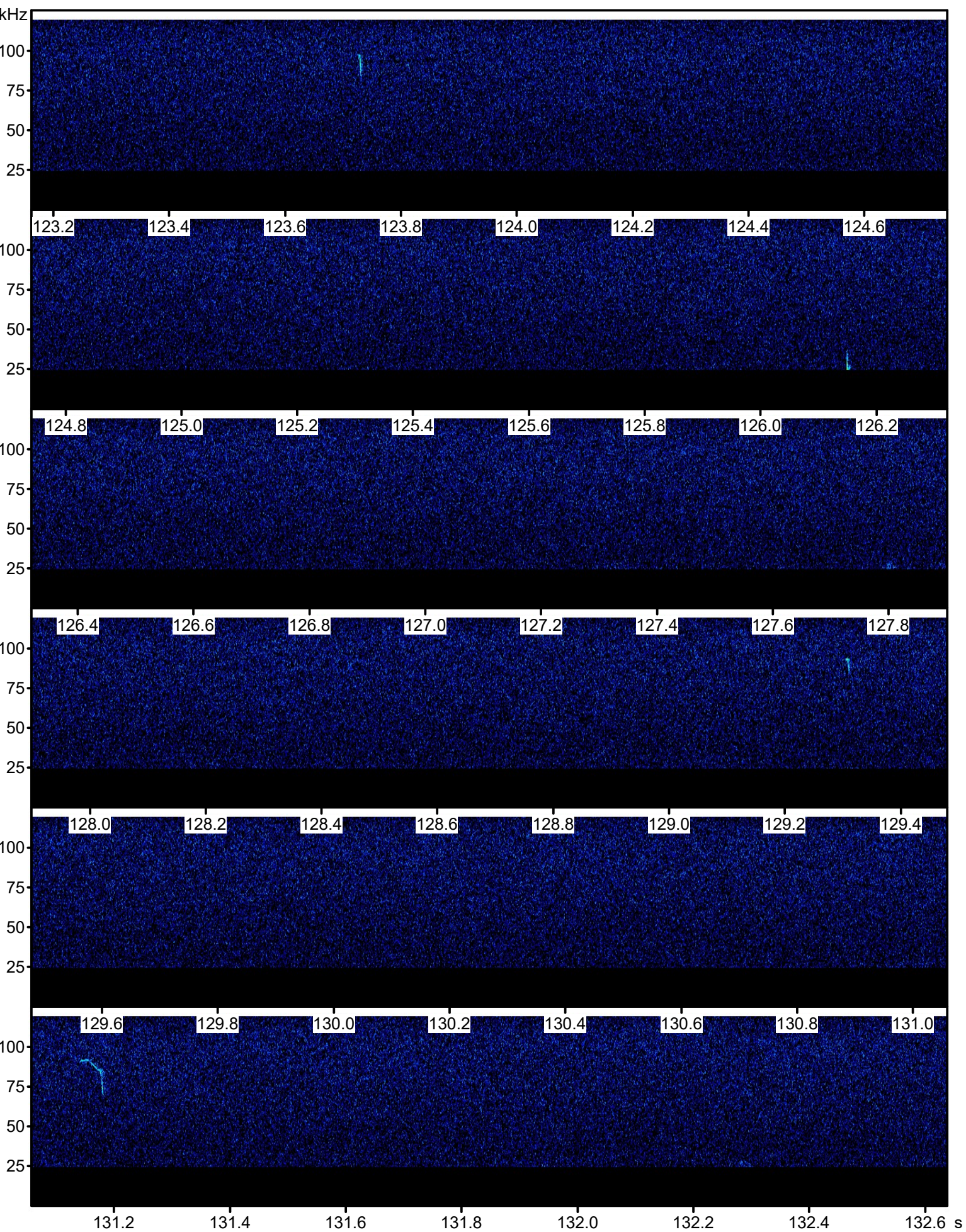

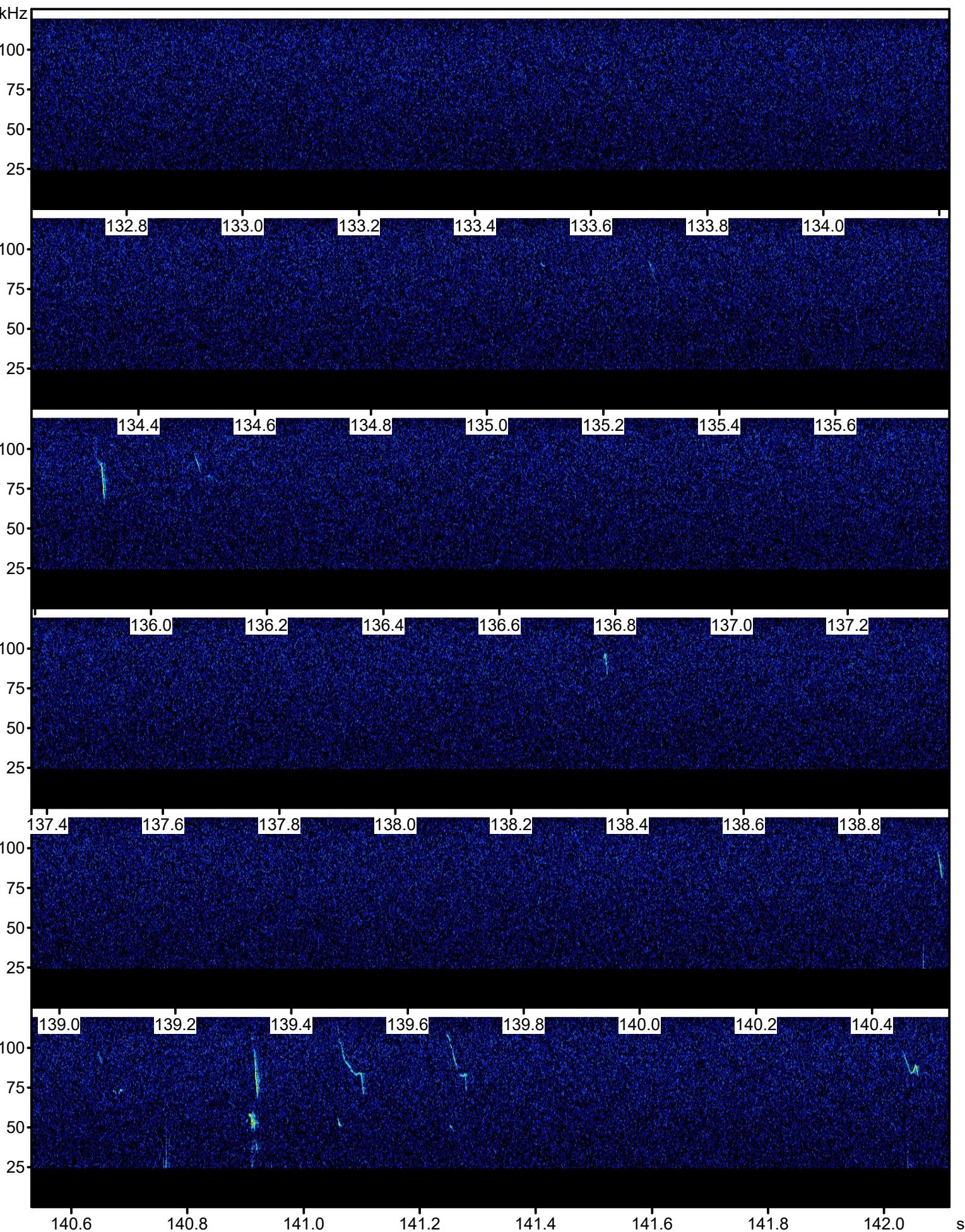

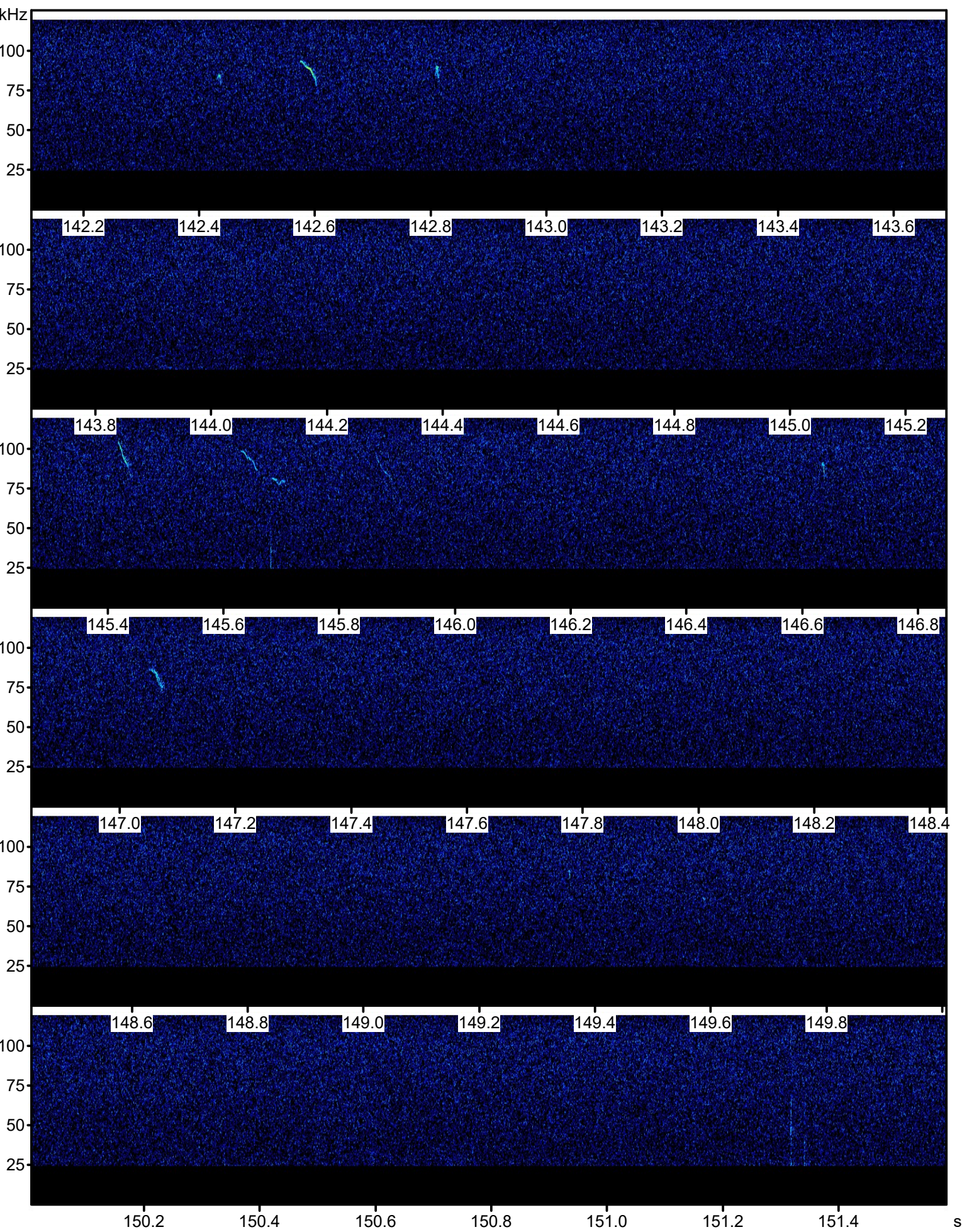

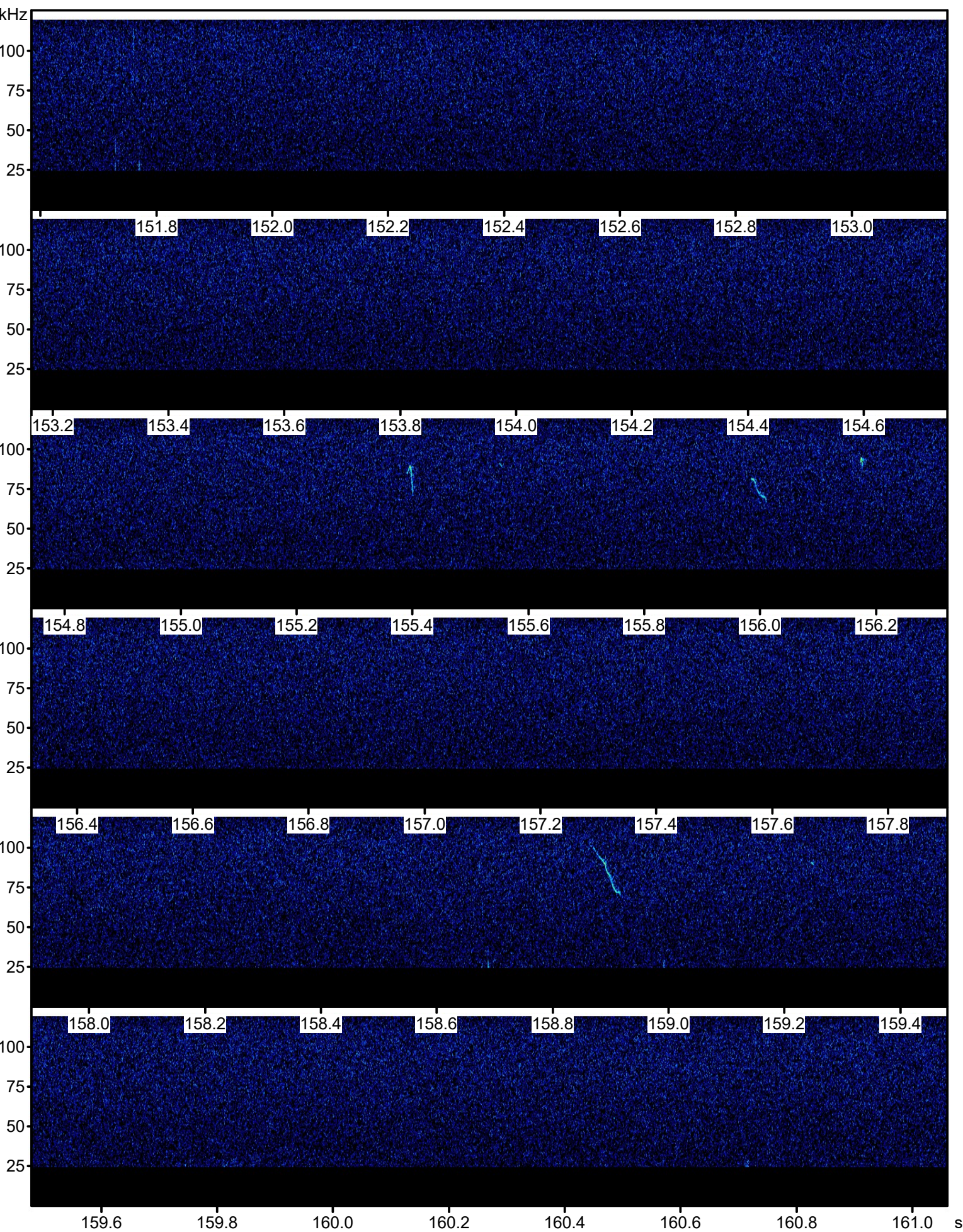

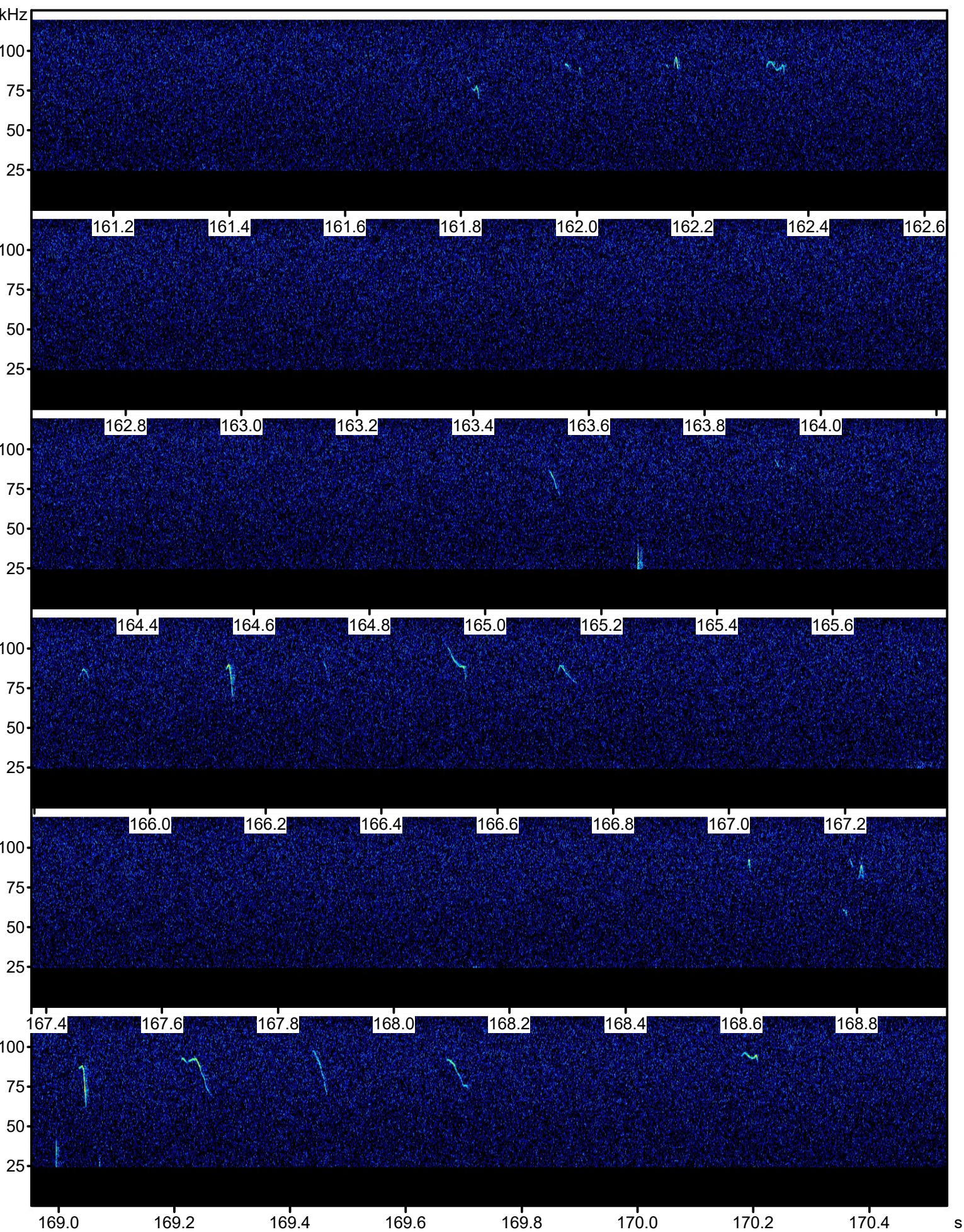

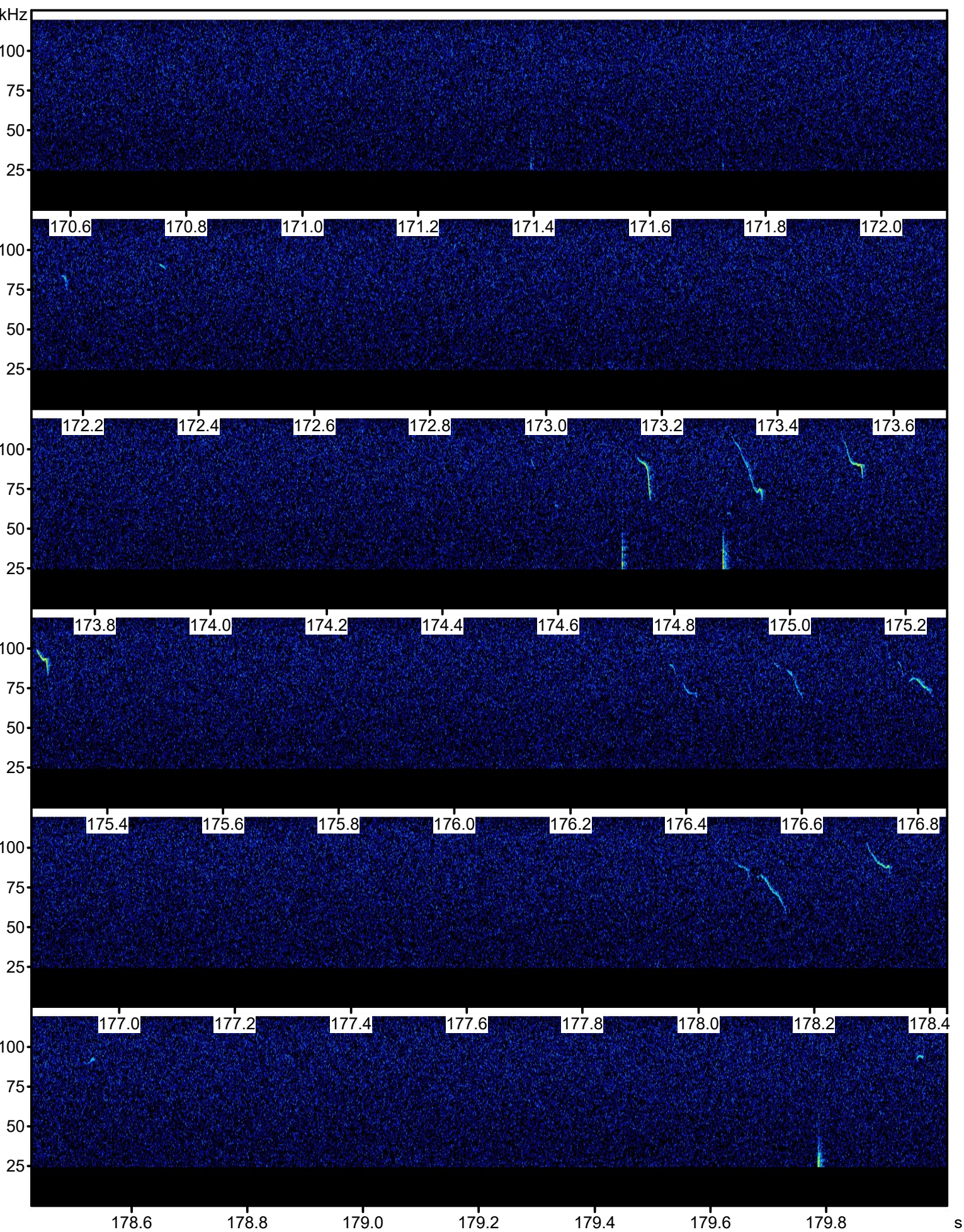

Supplement: Supplementary file 1 — Additional file 1. Full spectrogram of ultrasonic vocalization recording for a representative MPS IIIB mouse. [file 11689_2024_9534_MOESM1_ESM.pdf]

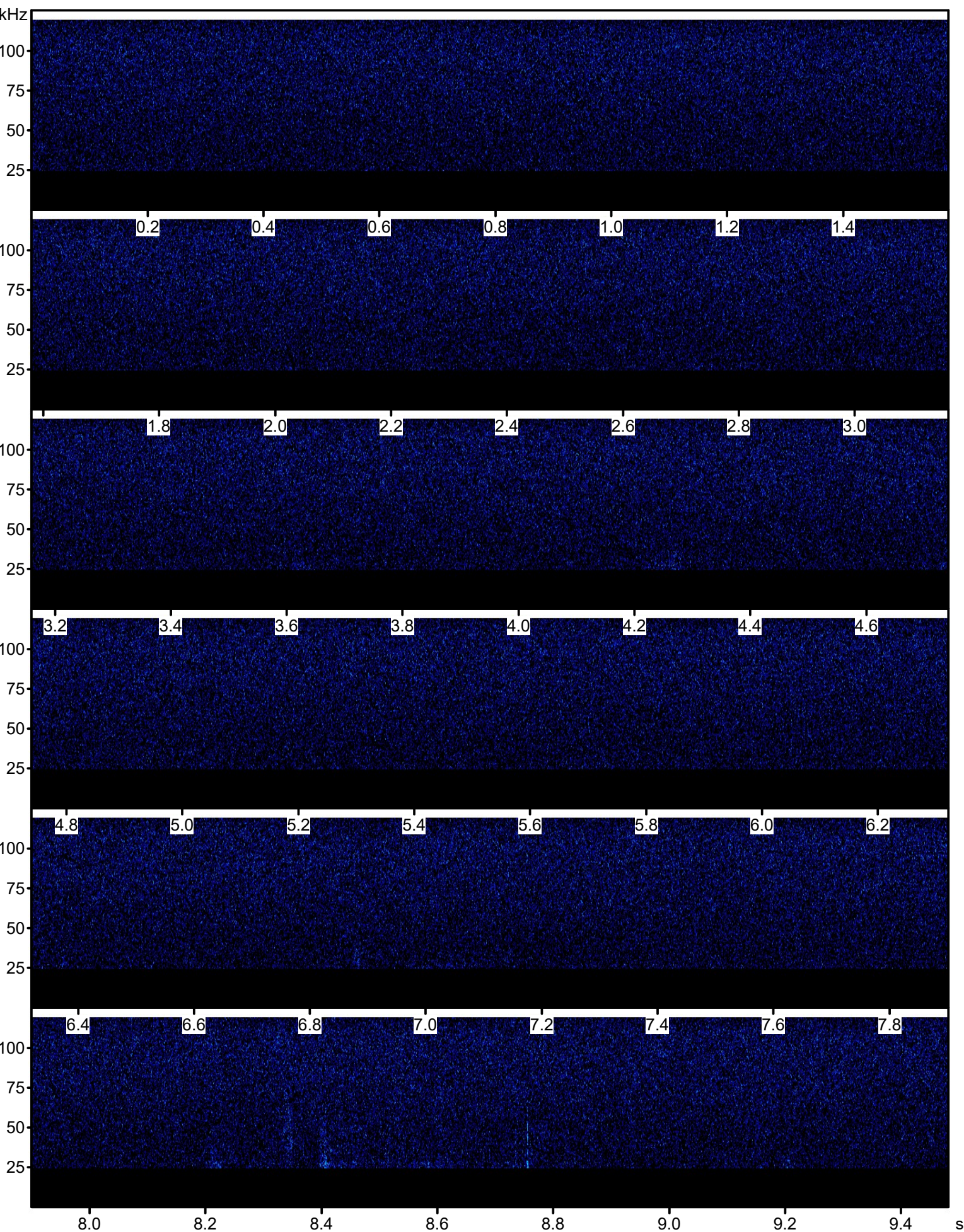

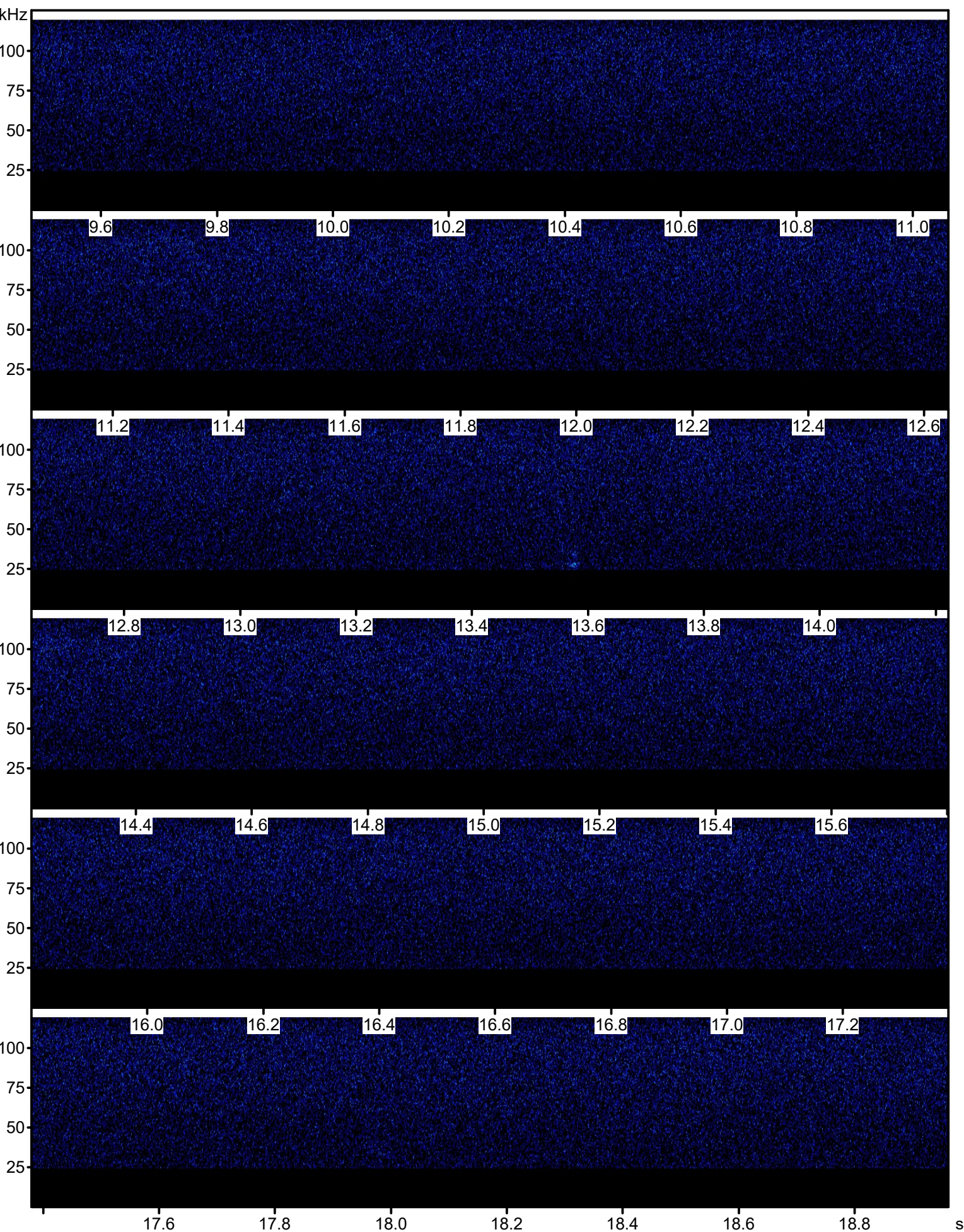

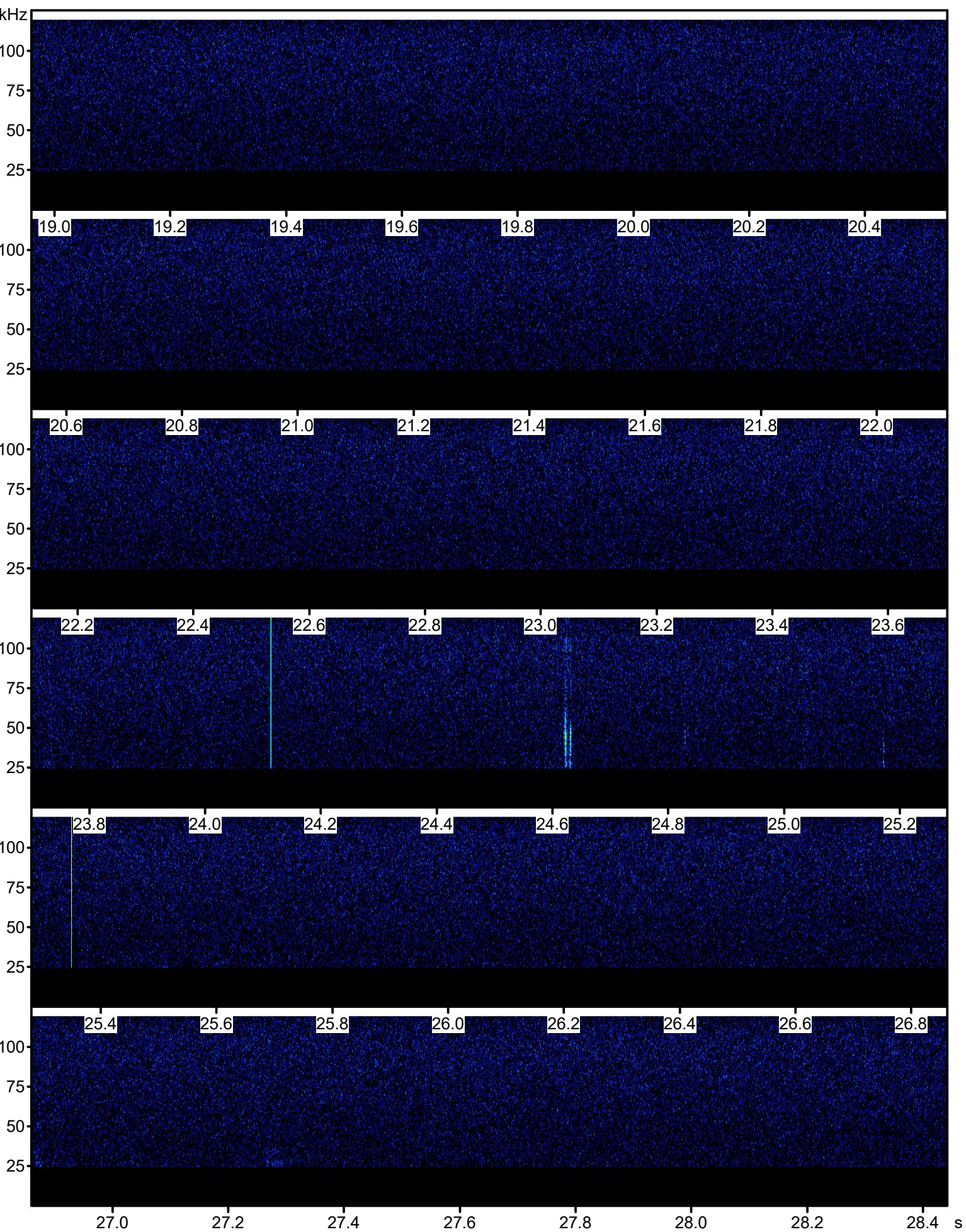

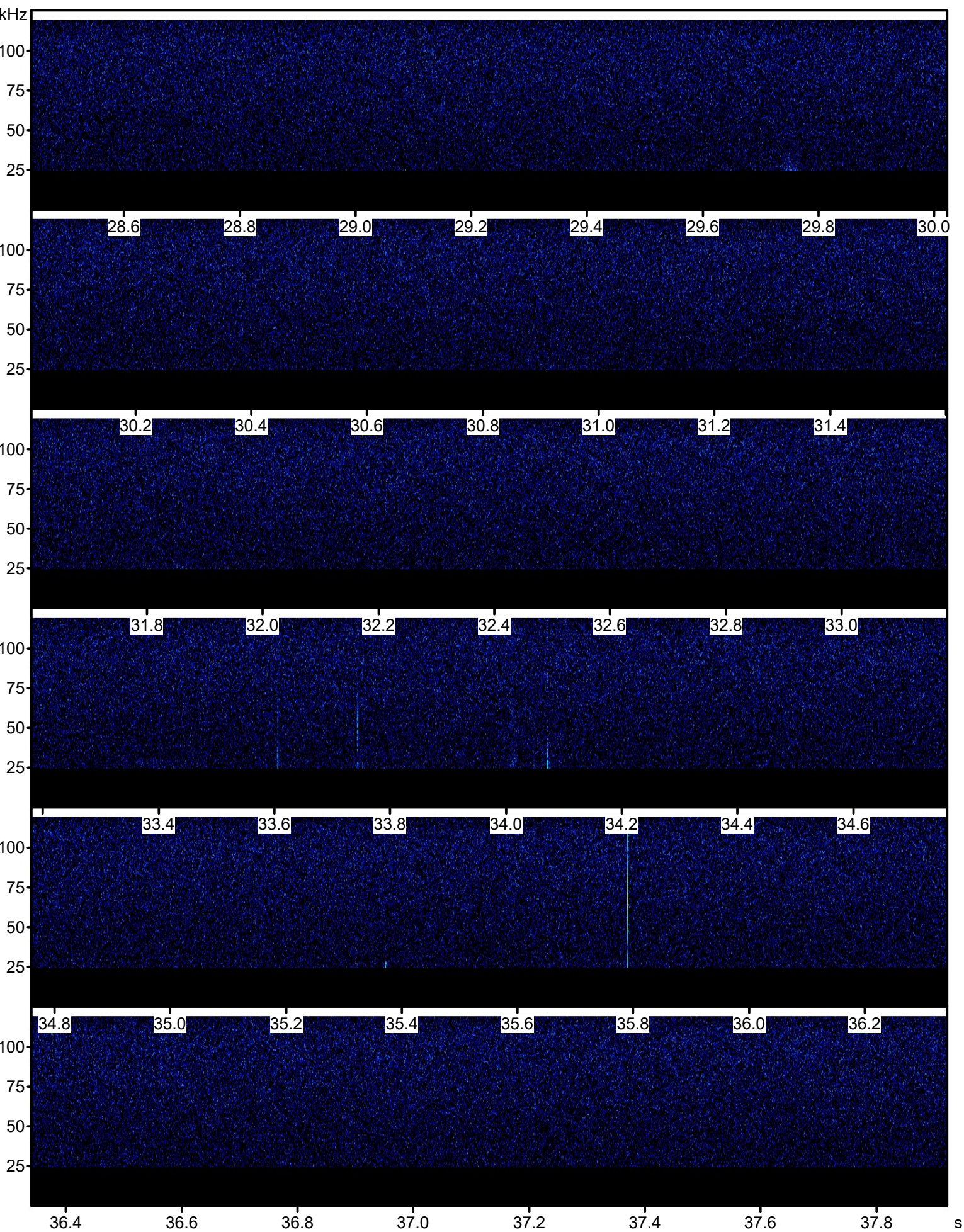

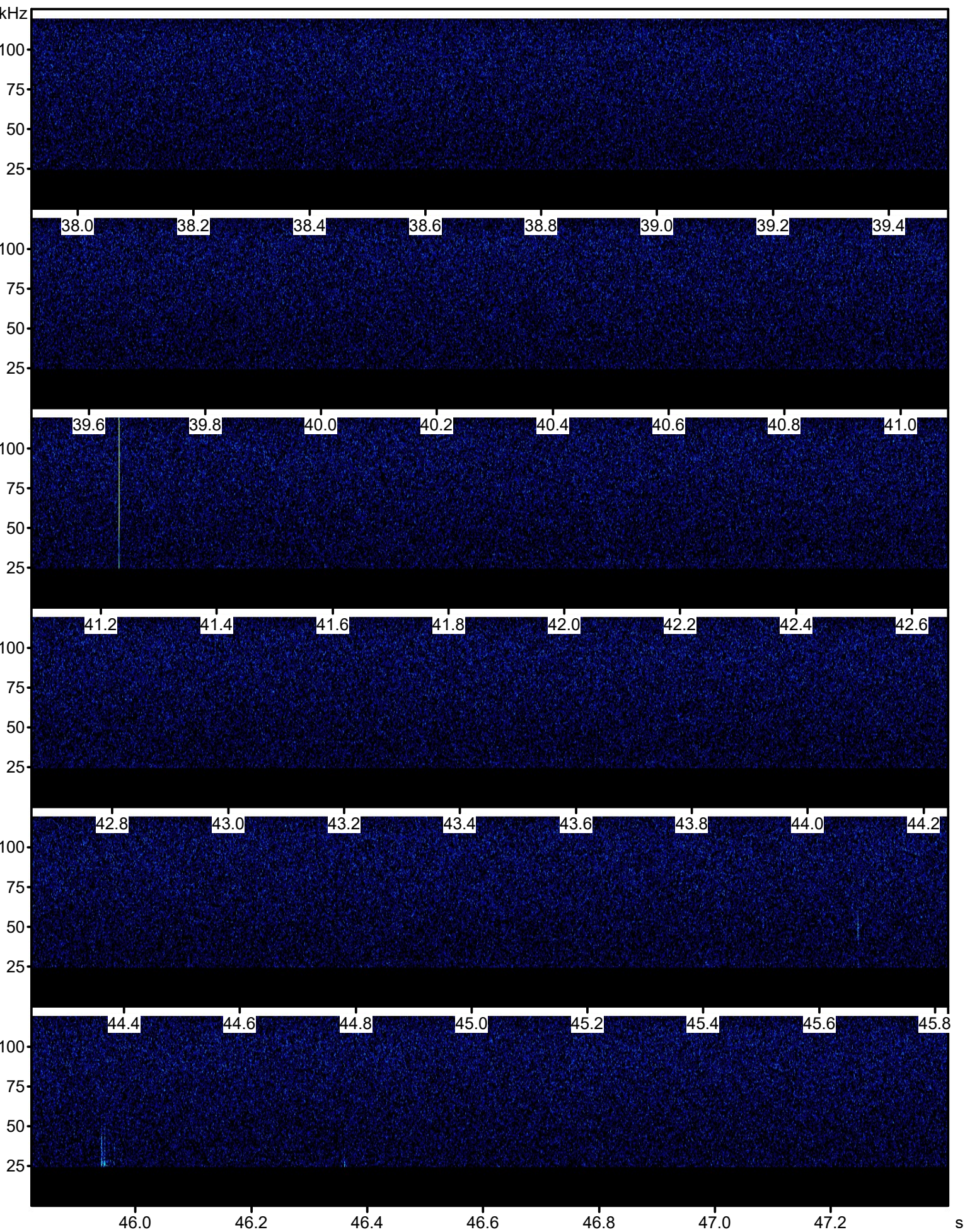

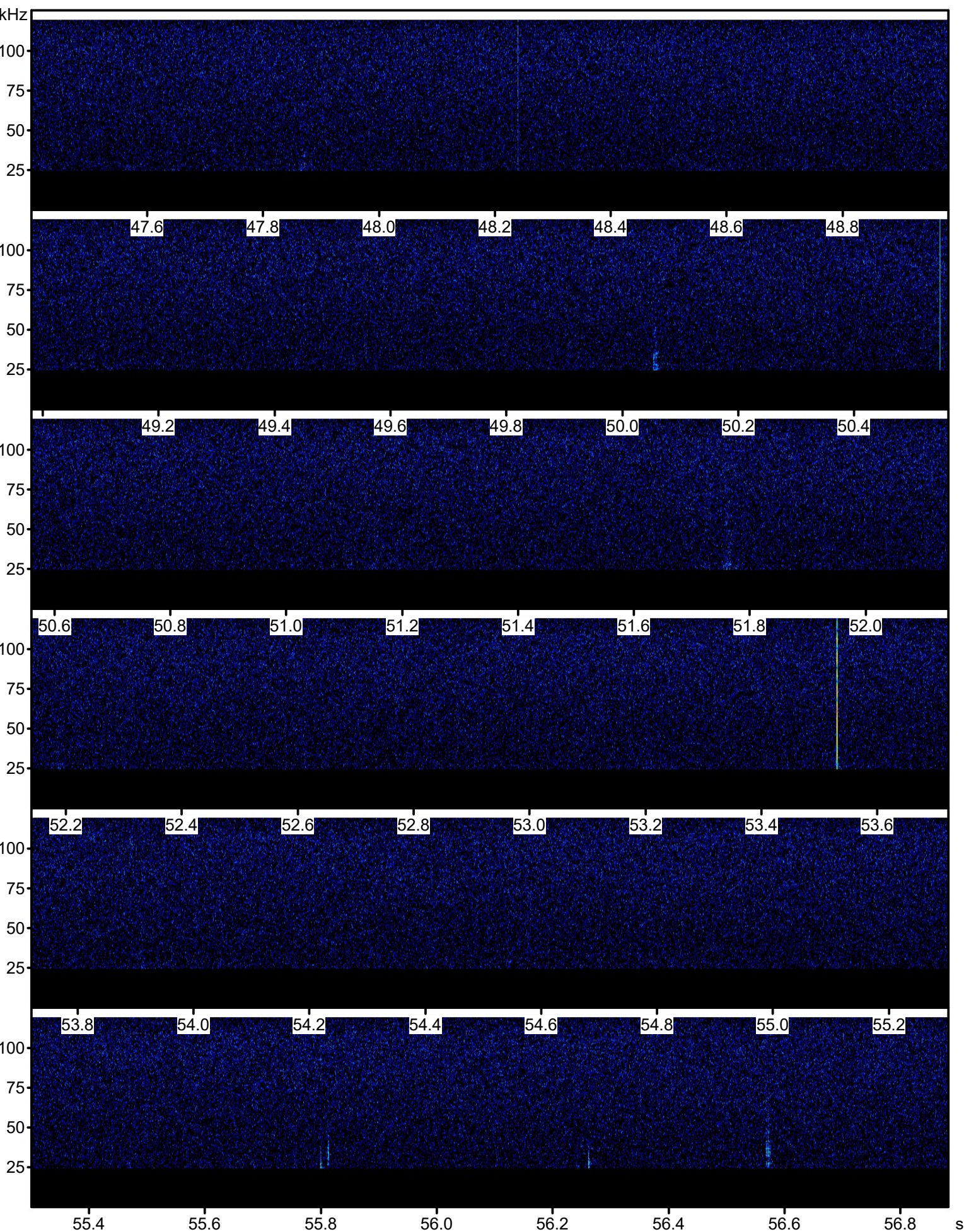

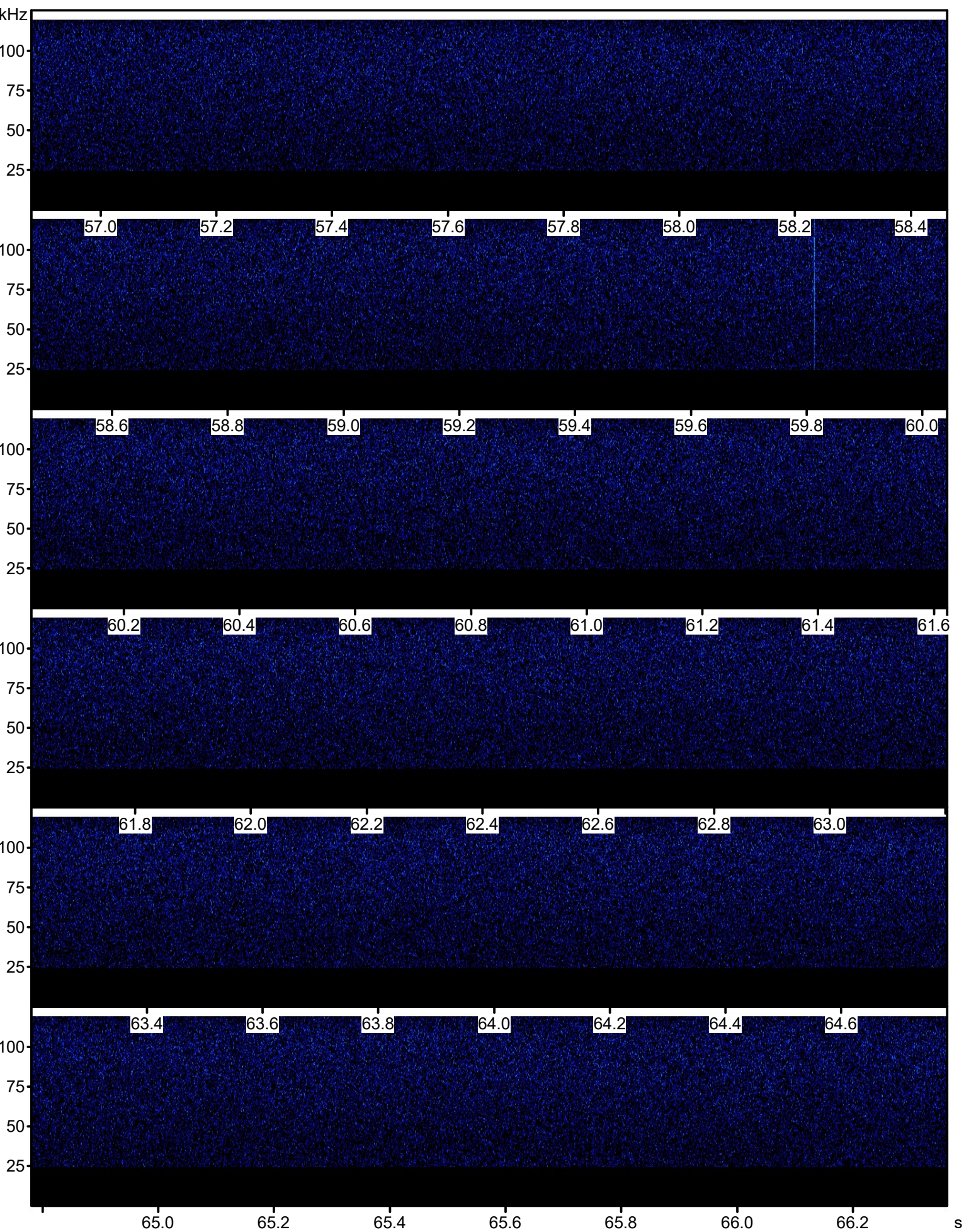

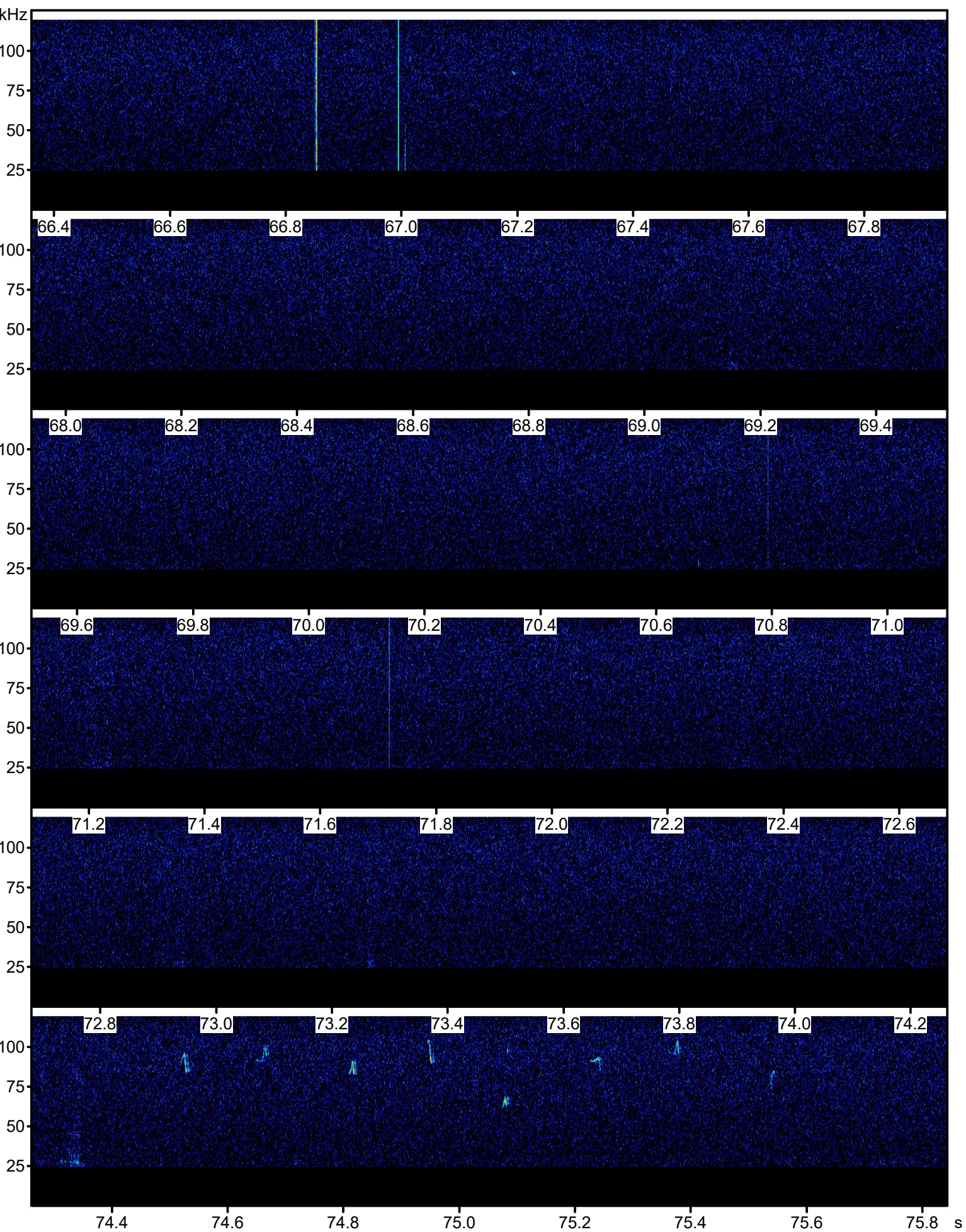

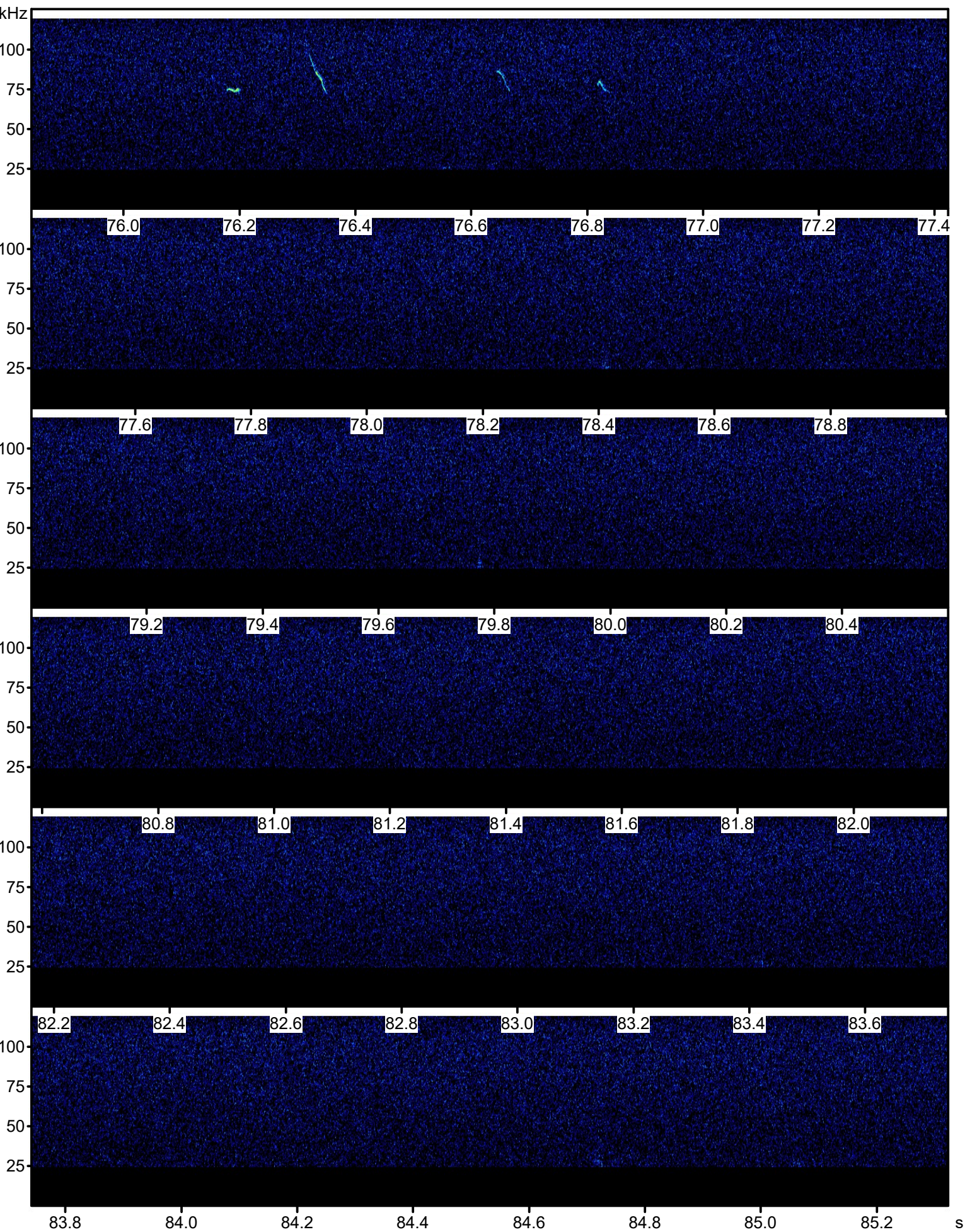

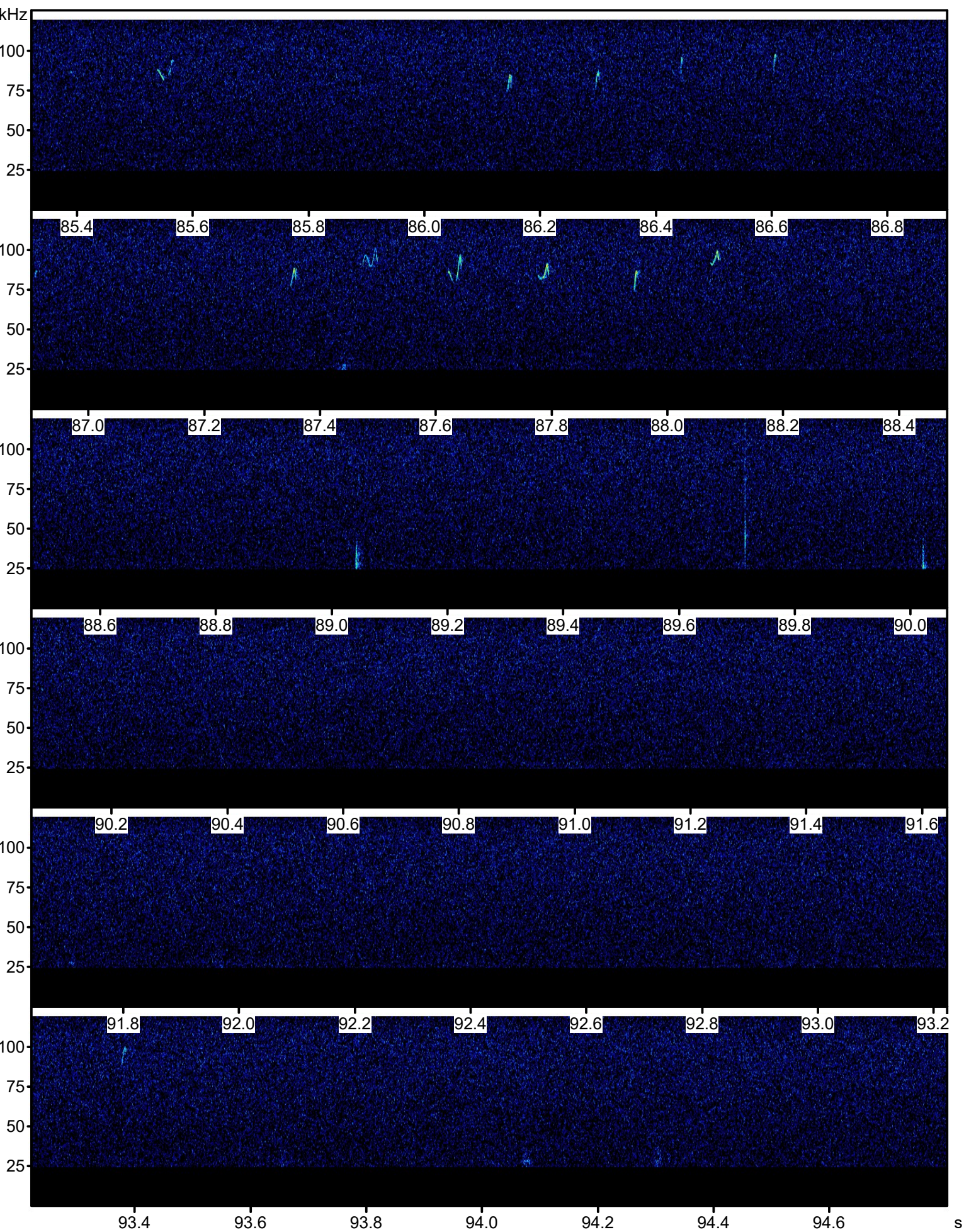

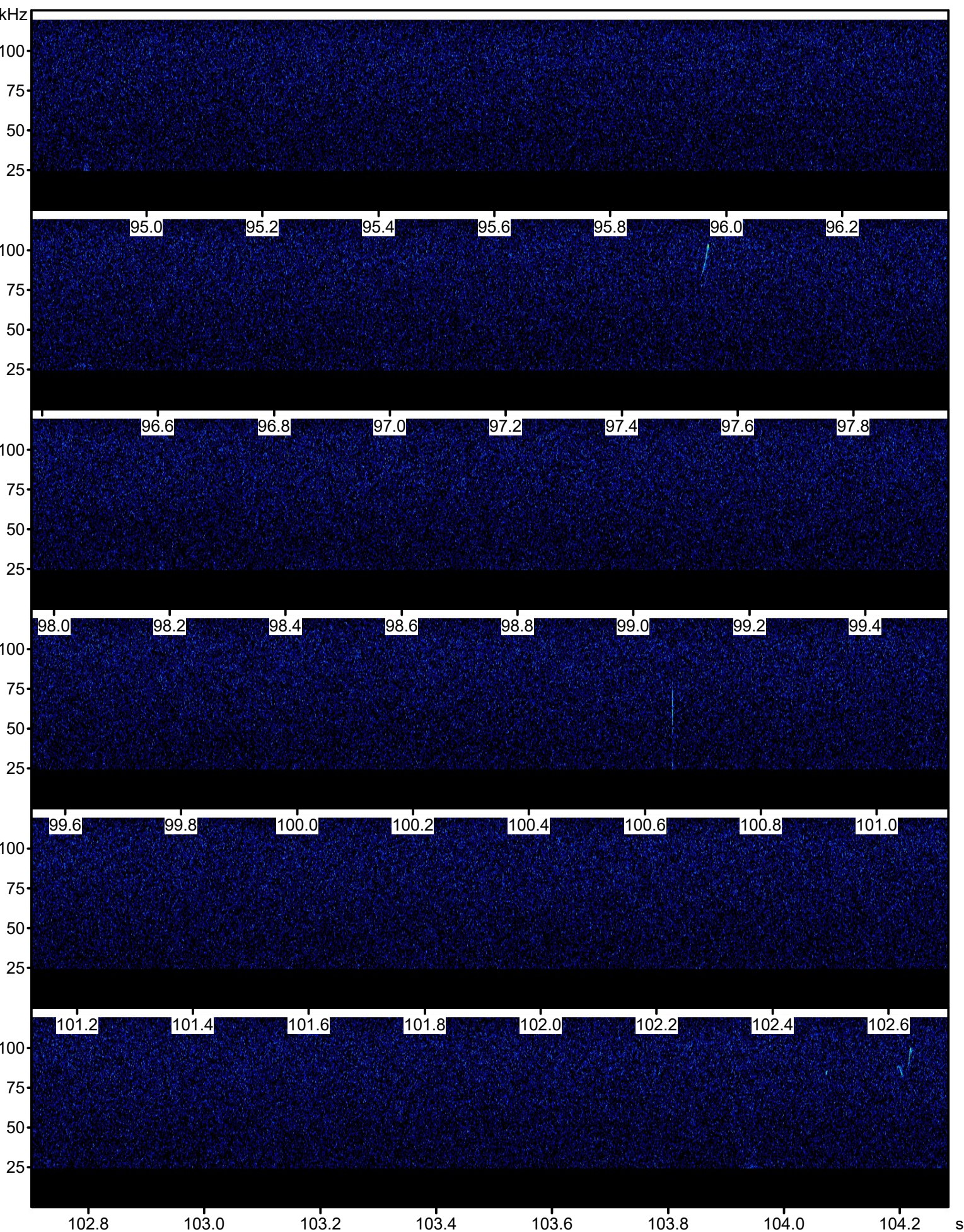

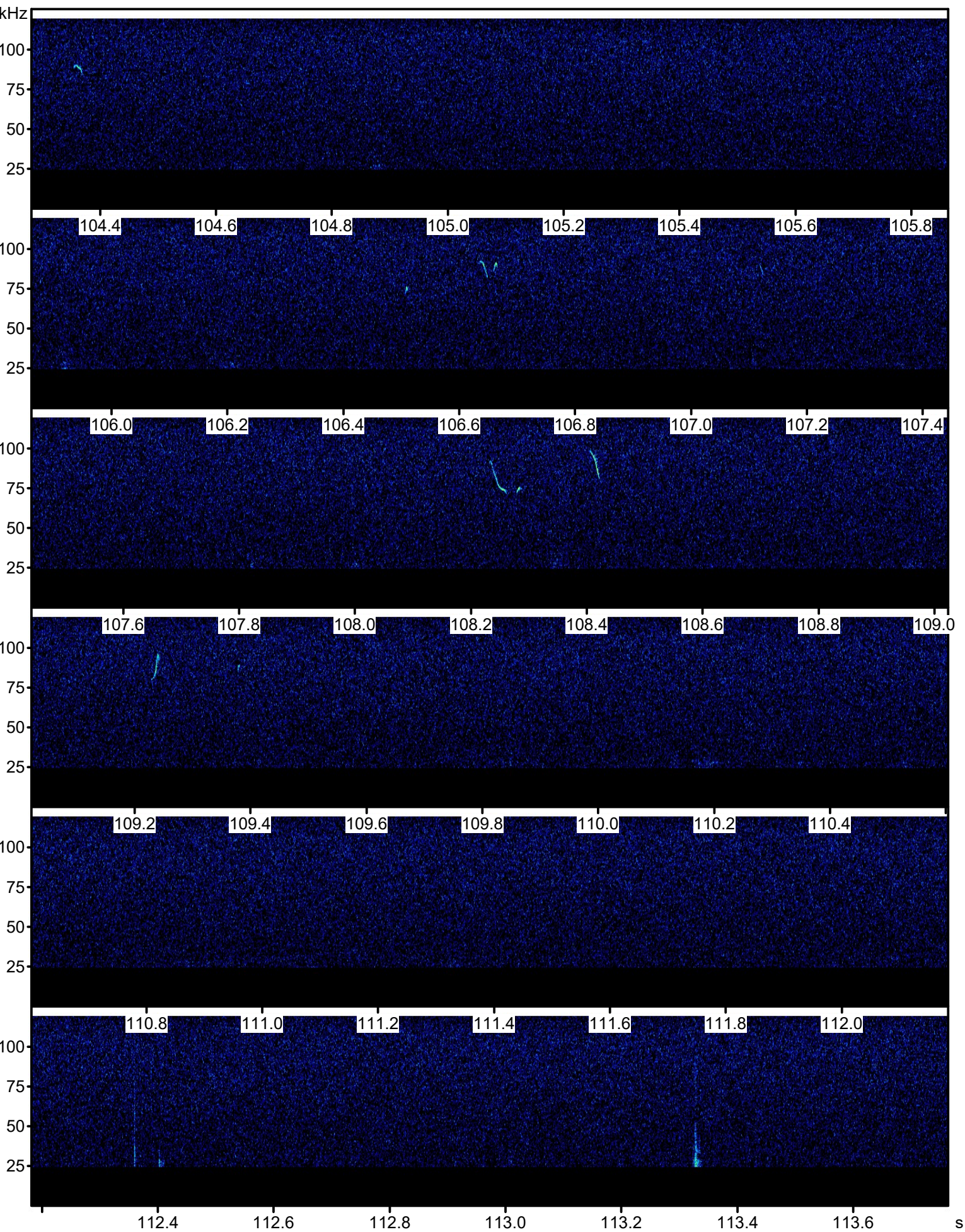

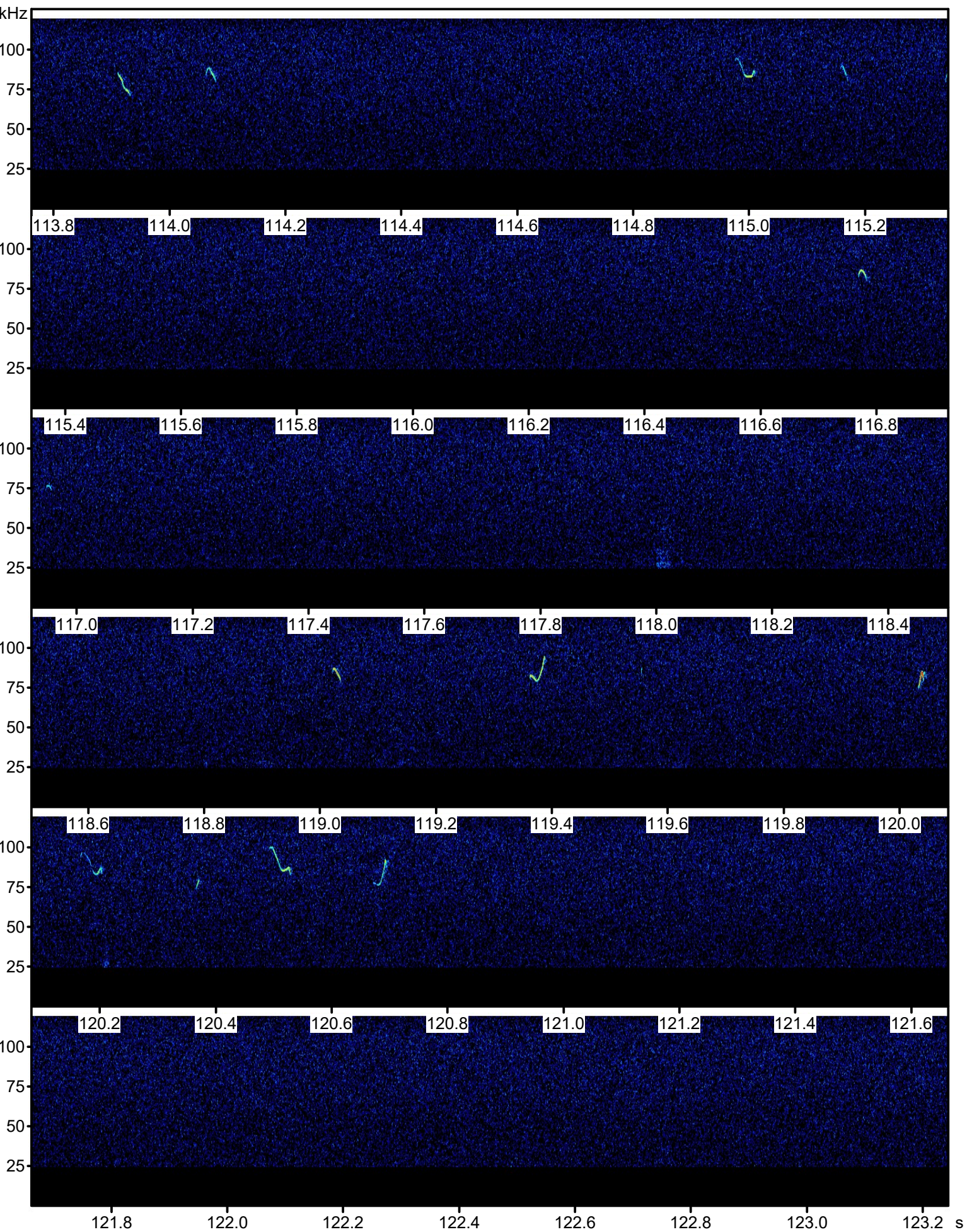

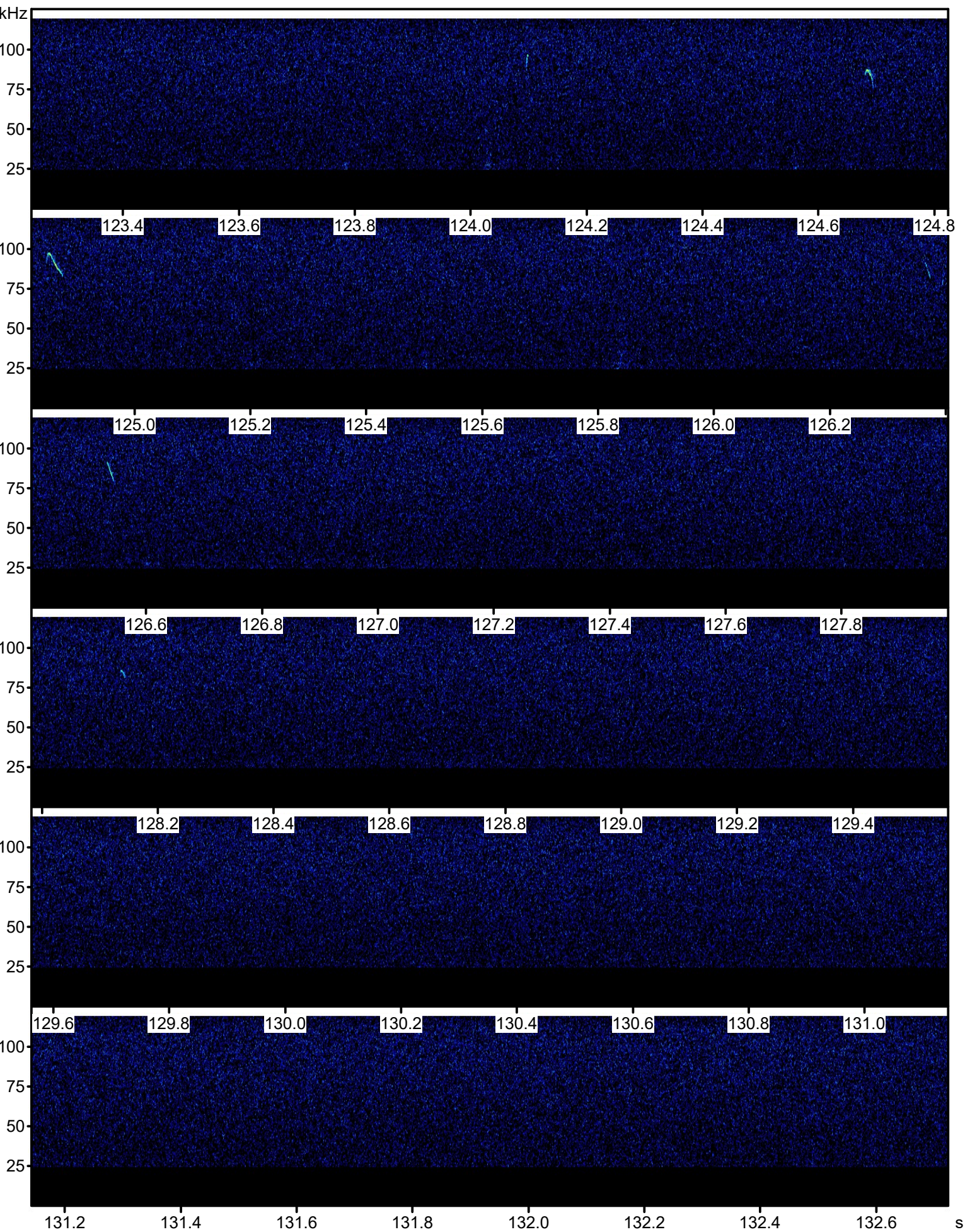

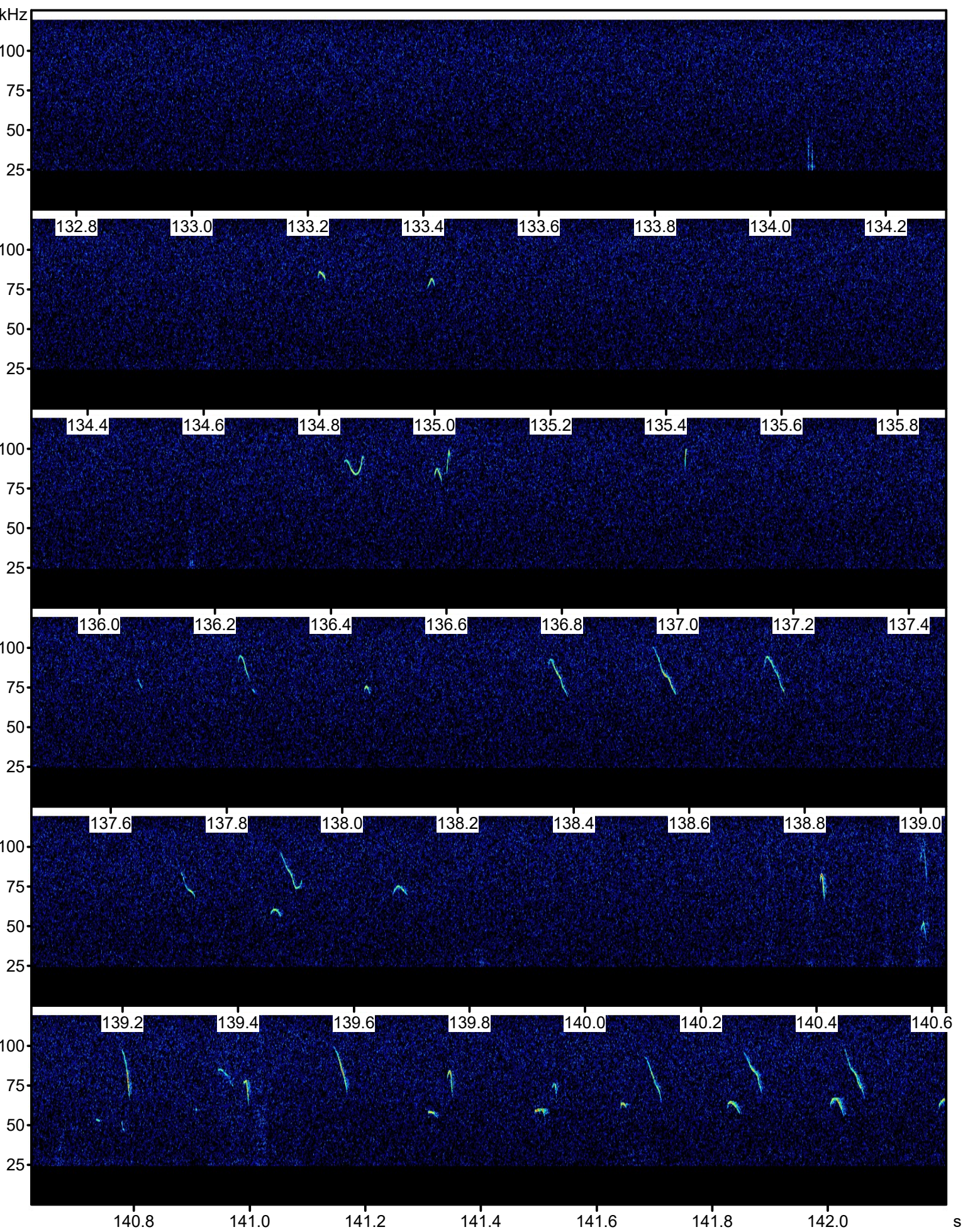

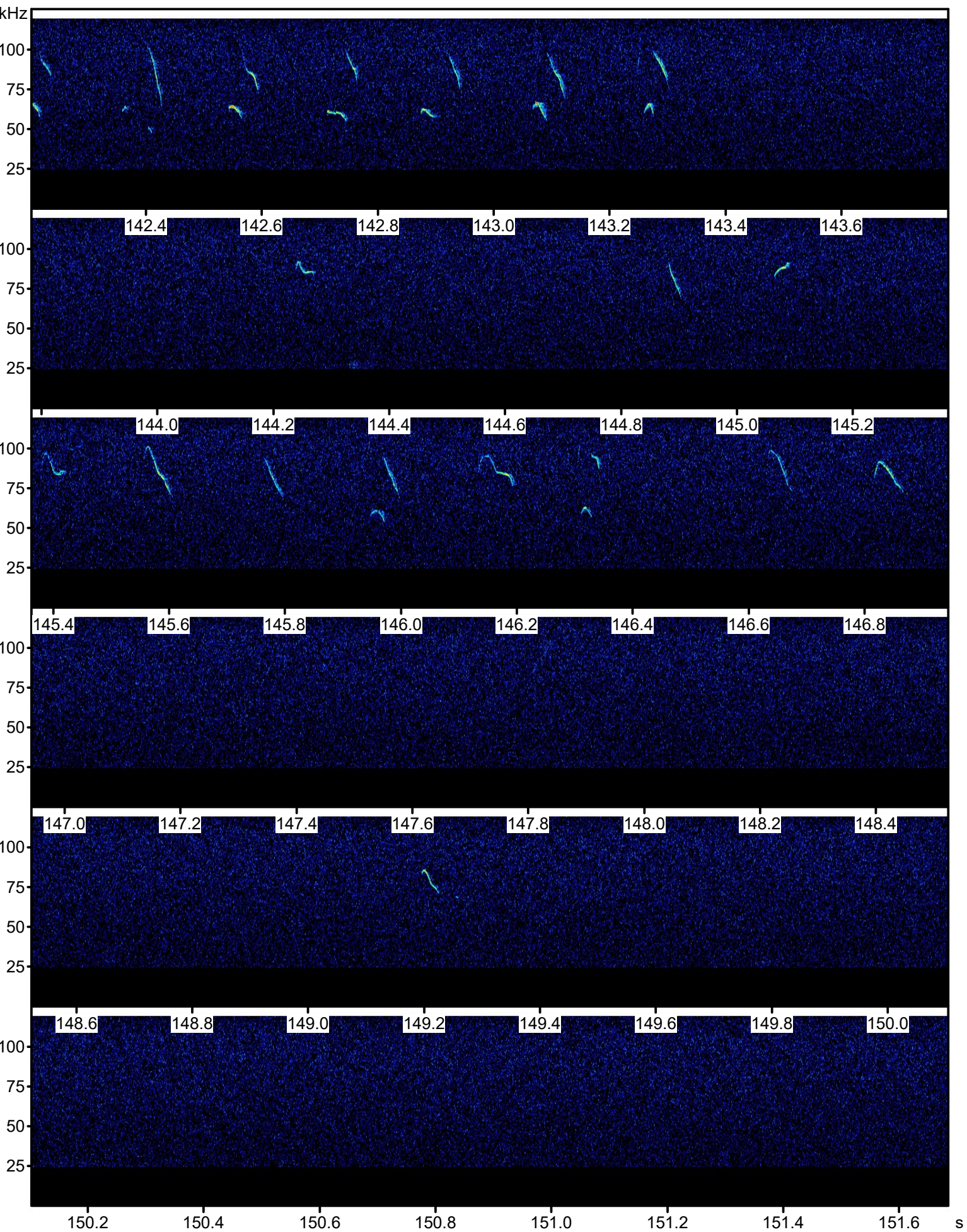

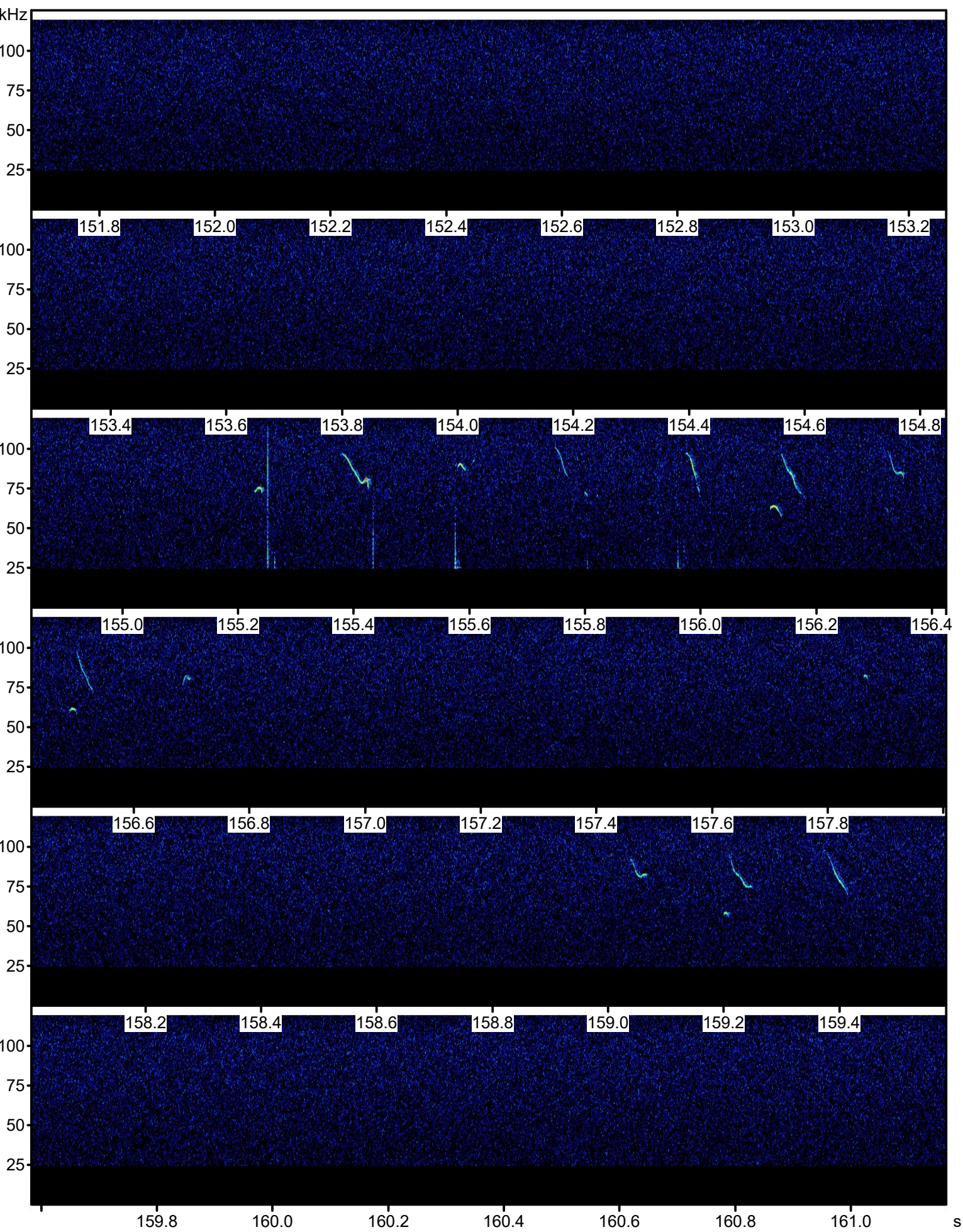

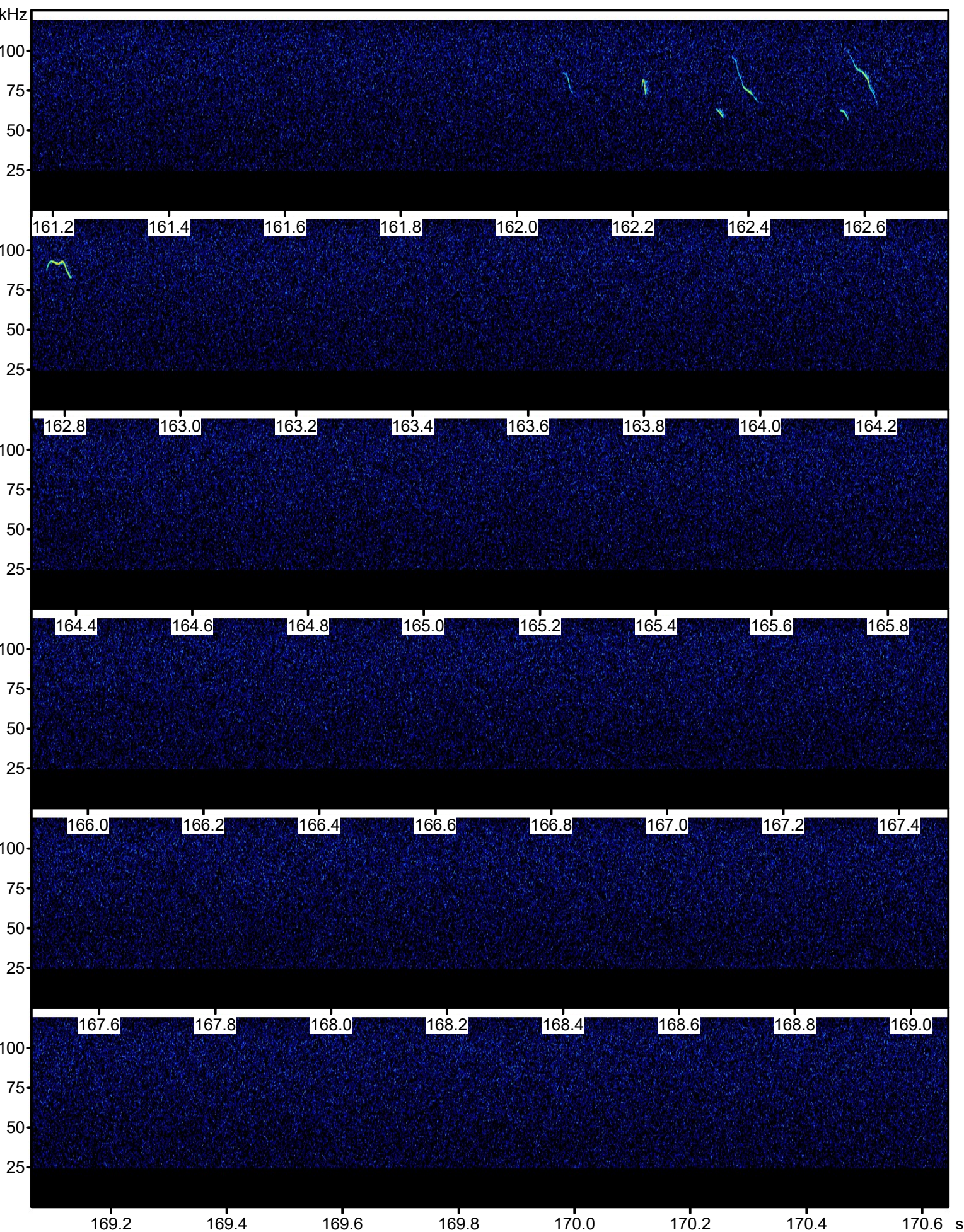

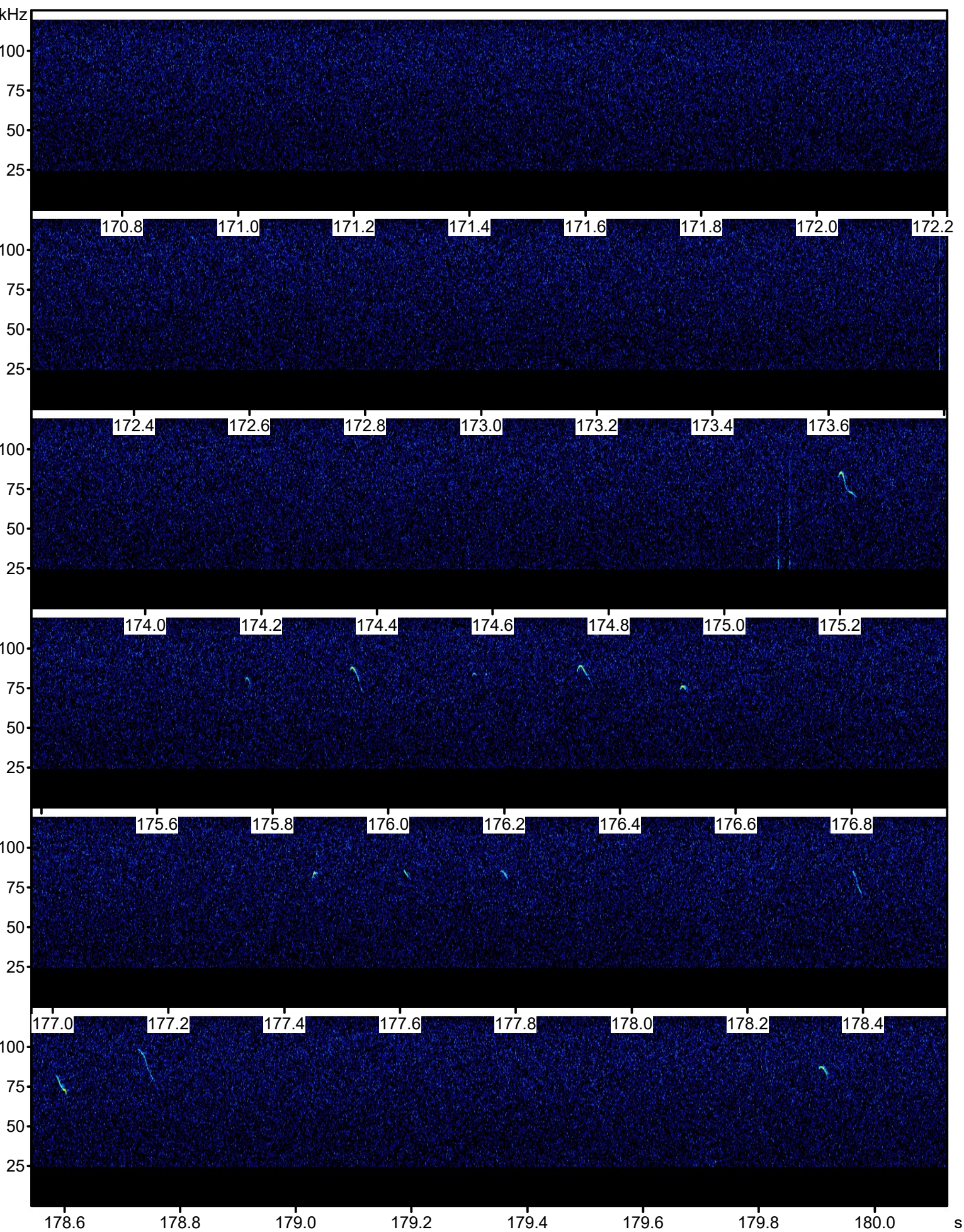

Supplement: Supplementary file 2 — Additional file 2. Full spectrogram of ultrasonic vocalization recording for a representative control mouse. [file 11689_2024_9534_MOESM2_ESM.pdf]
